# Supplementary material for: Tracking single particles for hours via continuous DNA-mediated fluorophore exchange
Source: Nat Commun. 2021 Jul 21;12:4432. doi: 10.1038/s41467-021-24223-4 (PMC8295357; doi:10.1038/s41467-021-24223-4)
Supplement: Supplementary file 1 — Supplementary Information [file 41467_2021_24223_MOESM1_ESM.pdf]

# Supporting Information

## Tracking Single Particles for Hours via continuous DNA-mediated Fluorophore Exchange

Florian Stehr[1,*], Johannes Stein[1,*], Julian Bauer[1], Christian Niederauer[3], Ralf Jungmann[1,2], Kristina Ganzinger[3] and Petra Schwille[2]

**Supplementary Figures**



| | |
|---|---|
| **Supplementary Figure 1** | **Size comparison between SPT labels** |
| **Supplementary Figure 2** | **Sequence design** |
| **Supplementary Figure 3** | **Data analysis workflow - immobilized** |
| **Supplementary Figure 4** | **Filter - immobilized** |
| **Supplementary Figure 5** | **Photon counts & localization precision - immobilized** |
| **Supplementary Figure 6** | **Error estimation for $T_{1/2}$ results** |
| **Supplementary Figure 7** | **Data analysis workflow - mobile** |
| **Supplementary Figure 8** | **Longest trajectories at varying irradiances** |
| **Supplementary Figure 9** | **Tracks, TPP and diffusion constant for varying irradiances** |
| **Supplementary Figure 10** | **Linking and particle density** |
| **Supplementary Figure 11** | **Photons counts - mobile** |
| **Supplementary Figure 12** | **$D$ vs. $D_{sub}$ for varying subtrajectory durations** |
| **Supplementary Figure 13** | **RMSD of $D_{sub}$ for varying subtrajectory durations** |
| **Supplementary Figure 14** | **2-fold slowdown of every fourth subtrajectory** |

**Supplementary Note 1**: Tracking handle key parameters

| | |
|---|---|
| Supplementary Figure 15 | Tracking handle key parameters overview |
| Supplementary Figure 16 | Extended 3-hour measurement using POCT |
| Supplementary Figure 17 | $k_{photodamage}$ vs. irradiance |
| Supplementary Figure 18 | Temperature effect on DNA hybridization rates |
| Supplementary Figure 19 | Ion concentration effect on DNA hybridization rates |

**Supplementary Note 2**: TPP calculus for mobile particles

**Supplementary Note 3**: Statistical treatment of diffusion coefficients

| | |
|---|---|
| Supplementary Figure 20 | Mobile analysis control with immobilized data set |

**Supplementary Note 4:** Potential limitations and workarounds

| | |
|---|---|
| Supplementary Figure 21 | Performance of tracking handle at varying TIRF angles |
| Supplementary Figure 22 | Performance of tracking handle at varying imager concentrations |
| Supplementary Figure 23 | 2×TH vs. 1×TH labeling |

**Supplementary Tables**

| | |
|---|---|
| Supplementary Table 1 | Imaging parameters |
| Supplementary Table 2 | Used DNA oligonucleotide sequences as labels |

**Supplementary References**

## Supplementary Figures

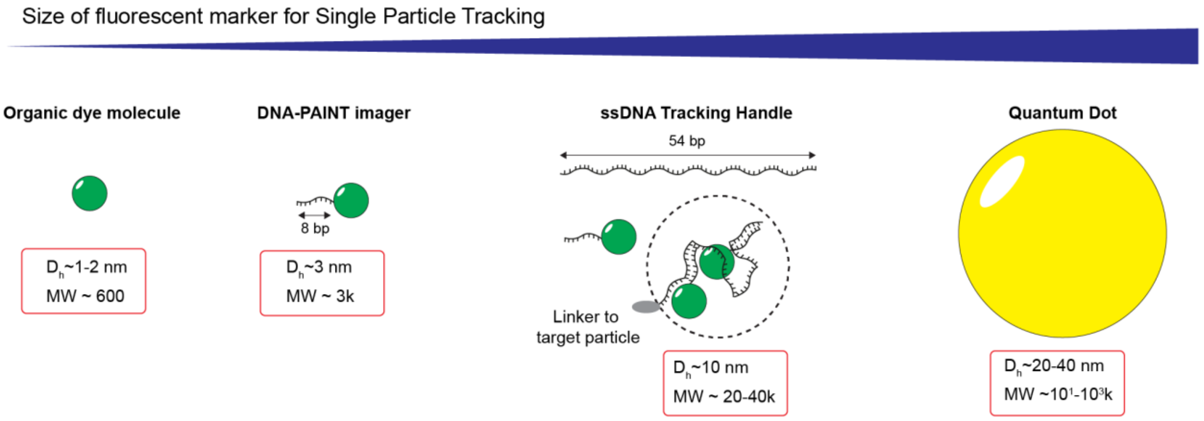


**Supplementary Figure 1. Size comparison between SPT labels.** True to scale estimation of the size (assuming a spherical shape with hydrodynamic radius $D_h$) and the molecular weight (MW) of an organic dye, a DNA-PAINT imager, the TH and a quantum dot. The $D_h$ estimation of the TH takes into account the flexible nature of ssDNA assuming coiling due to unbound regions. The MW estimation of the TH assumes on average 3x bound imagers.

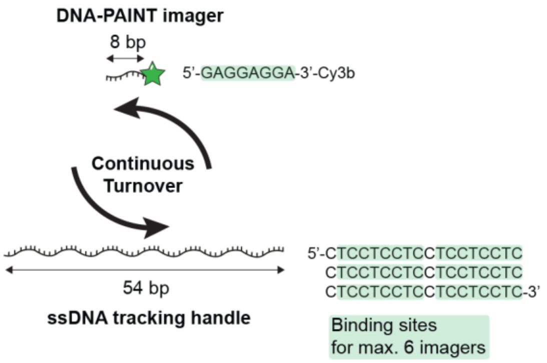


**Supplementary Figure 2. Sequence design.** Sequence design of the 54 base-pair ssDNA tracking handle (TH) and DNA-PAINT imagers.

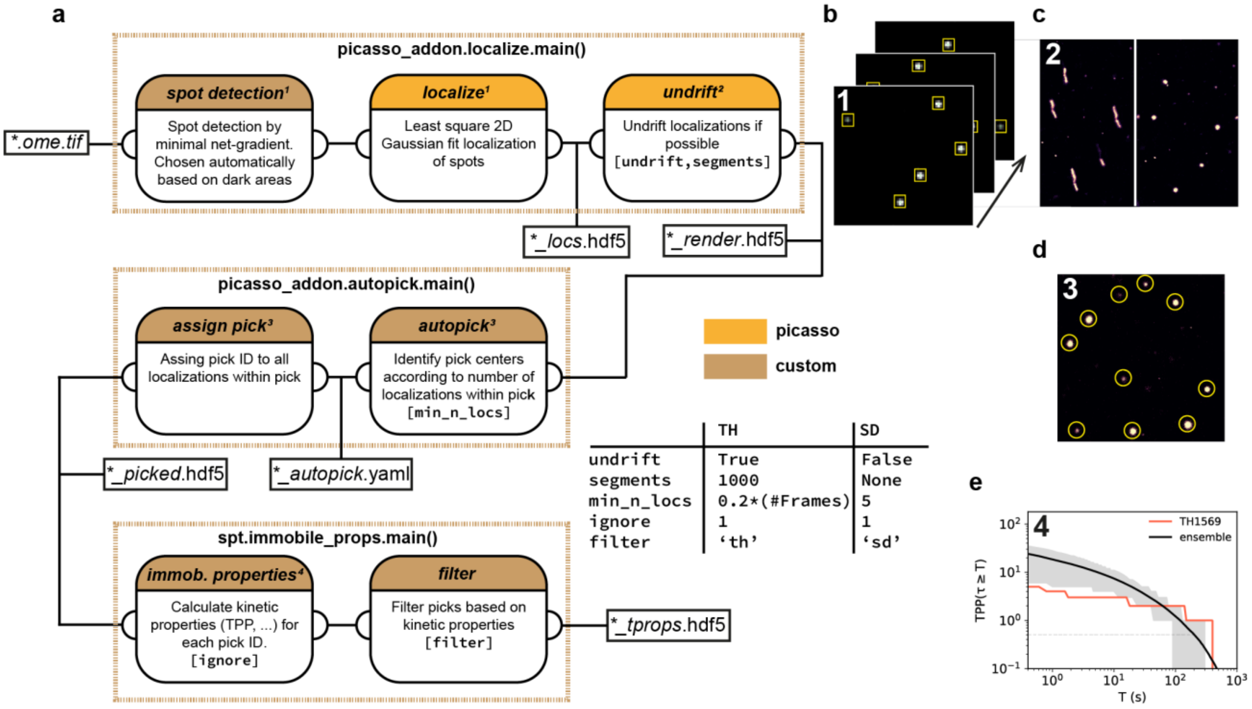


**Supplementary Figure 3. Data analysis workflow - immobilized.** (**a**) Data analysis workflow from raw movies (*.ome.tif) to final result (*_tprops.hdf5). Dashed boxes indicate employed main functions - e.g. picasso_addon.localize.main() - of the *picasso_addon* and *spt* python package. Rounded boxes illustrate provided functionalities of each main function. The header color code indicates from which python package the functionality was adapted (e.g. *picasso* or *custom* for extended functionalities within *picasso_addon* or *spt*). The text within the rounded boxes gives a short description of the provided functionality and parameters (brackets) for execution of the main function. The table summarizes all parameters used for evaluation of immobilized SD or TH experiments. The small boxes branching of the main flow represent which files are saved during execution. Please visit the links provided in the section "**Image processing & single particle tracking analysis**" for further information. (**b**) Illustration of spot detection (boxes) and localization of individual emitters in raw images. (**c**) Illustration of image correlation based undrifting of the rendered localization lists (parameters: undrift, segments). (**d**) Illustration of localization cluster detection based on number of localizations within the localization cluster (parameter: min_n_locs). We follow the Picasso nomenclature referring to a detected localization cluster as 'pick' with a unique pick ID. (**e**) Final result is obtained by calculating kinetic properties for each pick (e.g. TPP, number of localizations, photon counts etc.) by employing spt.immobile_props.main() (parameters: ignore, filter). For a detailed description of our final filtering procedure for each pick please refer to **Supplementary Fig. 4.**

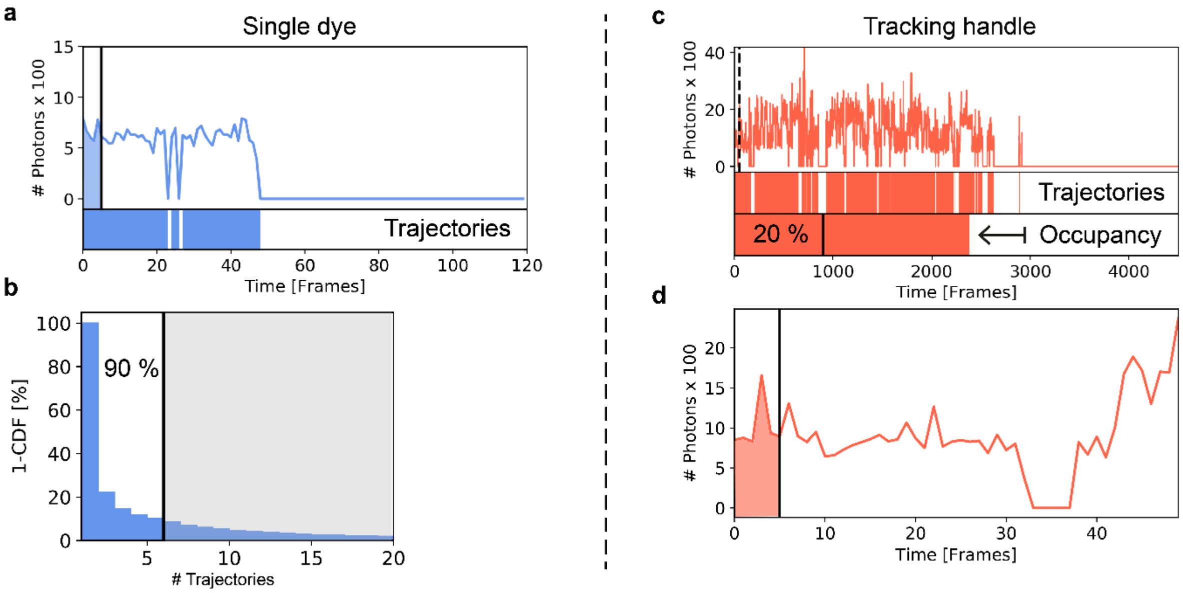


**Supplementary Figure 4. Filter - immobilized.** (**a**) The filtering procedure (see **Supplementary Fig. 3**) for immobilized SD origami is illustrated for an exemplary fluorescence trace (blue line). Valid picks (i.e. picks passing the filter criteria) yielded fluorescence traces with at least one localization within the first 5 frames (black line) of the measurement (blue area below trace). The bar below indicates the number of trajectories, i.e., continuous and uninterrupted fluorescence signal (here 3 trajectories). (**b**) Cumulative distribution function of number of trajectories per SD origami for an exemplary data set. As expected, the majority of SD origami yielded only a single trajectory before undergoing photobleaching. However, a small fraction of SD origami exhibited blinking behavior, causing interruptions in the fluorescence trace and hence an increased number of registered trajectories (as in the example in a). To remove potentially damaged/imperfect dye molecules, we discarded all picks exceeding the 90%-percentile (black line) of the distribution of number of trajectories of all picks, in this case picks exhibiting more than 5 trajectories. (**c**) For immobilized TH origami we calculated the ratio between the total time of a TH in the fluorescent state (i.e. the sum over all trajectory durations) and the total measurement time, which we define as the occupancy. In other words, the occupancy indicates the total time in which a TH is occupied with a fluorescing imager as a percentage of measurement time. Only picks with an occupancy of more than 20 % (black line) were used for further analysis. The black dashed line indicates a zoom into the fluorescence signal shown in (**d**). Analogous to (a), we additionally only considered TH origami picks yielding at least one localization within the first 5 frames (black line) of the measurement (red area below trace).

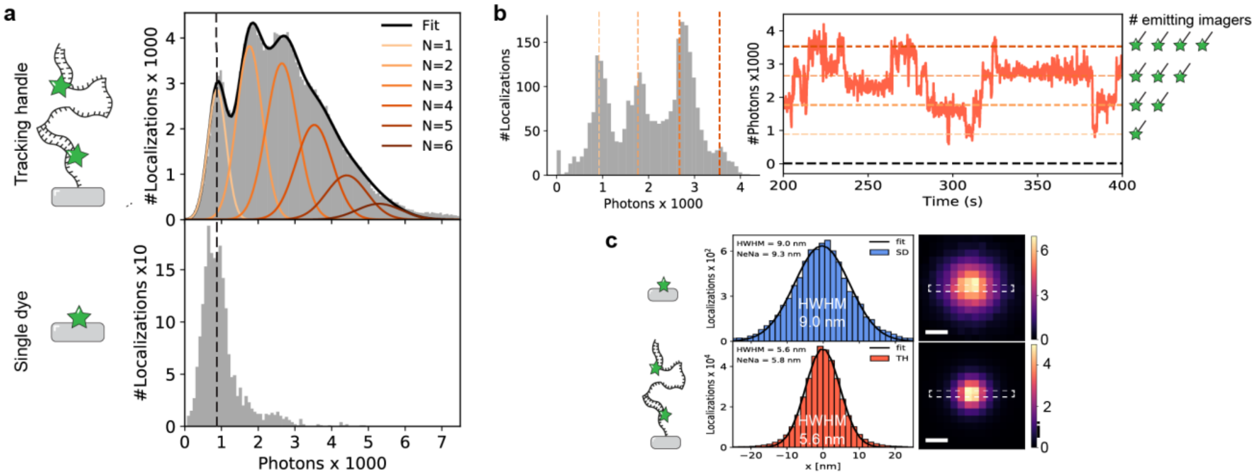


**Supplementary Figure 5. Photon counts & localization precision - immobilized.** (**a**) Ensemble photon count distributions as obtained from localizations corresponding to immobilized TH origami (top) and SD origami (bottom). The TH origami showed a multimodal distribution with distinct peaks in an equidistant spacing and located at multiples of the lowest peak's center value. We hence fitted the data with the sum (solid black) of 6 Gaussian functions $g_i$ (colored) of the form $g_i(x; A_i, x_0, \sigma) = A_i \exp\left[\frac{(x-ix_0)^2}{i\sigma^2}\right]$ with freely floating amplitudes $A_i$ for each $g_i$ but global parameters $x_0$ and $\sigma$ for the sum, corresponding to the center and width of the lowest order peak. The amplitudes $A_i$ can thus directly be translated into a probability of $i$ emitting imager strands being bound to the TH. Notice that the fit result for the center of lowest peak $x_0$ coincides with the single peak obtained for the photon distributions of SD origami localizations (dashed black line). In order to account for imaging artifacts causing variations in the number of detected photons[5], we only used origami lying within the central circular region of the FOV (diameter = 200 px). (**b**) Photon count histogram (left) and zoom into the fluorescence trace (right) of the individual TH origami TH1569 displayed in **Fig. 1**g. The dashed colored lines indicate the photon levels $ix_0$ as obtained from the fit of the ensemble distribution (a, top). For individual origami, the equidistant peaks in the photons count histogram (left) revealing the number of currently bound and emitting imagers (here 1-4x imagers) are more clearly separable than in the ensemble histogram from all TH origami (a, top). The zoom-in (right) illustrates that the fluorescence trace indeed follows a step-like behavior dependent on the number of emitting imagers bound to the TH at every time point. (**c**) Analysis of the effect of the increased brightness of the TH compared to SD origami with respect to the localization precision. The left panel depicts cross-sectional histograms through the aligned and averaged images of several hundreds of both SD origami (top image and blue histogram) and TH origami (bottom image and orange histogram). The visual impression of sharper localization distribution for THs compared to SDs is confirmed by comparing the half width half maximum (HWHM) of the Gaussian fits to the two histograms (5.6 nm and 9.0 nm, respectively), which are in good agreement with the localization precision results based on Nearest-Neighbor Analysis[7] (NeNA; 5.8 nm and 9.3 nm, respectively). Particle averaging was repeated over at least $n$~2,000 origami. Scale bars, 10 nm in (c).

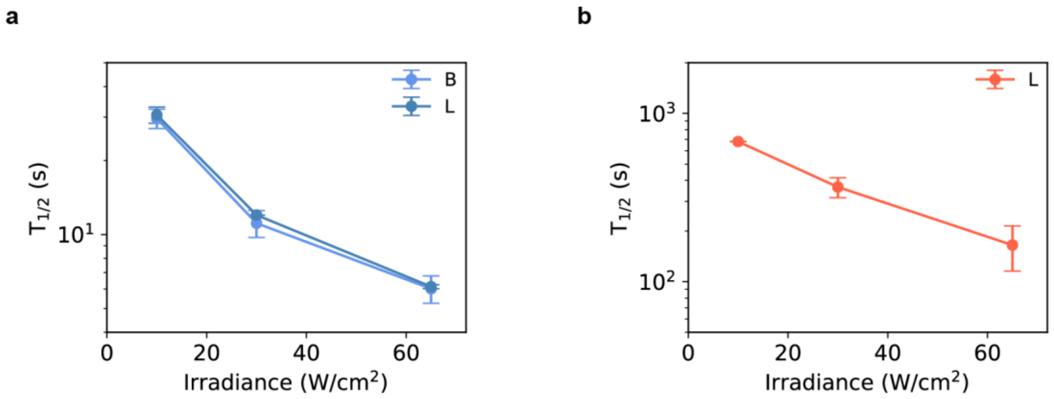


**Supplementary Figure 6. Error estimation for $T_{1/2}$ results.** (**a**) Repeated irradiance series for fresh samples of SD origami in buffer B (light blue; 4x samples; each sample imaged at 3x field of views (FOVs)) and buffer L (dark blue; 1x samples, 3x FOVs). The plots display the mean $T_{1/2}$ values averaged over all repeats per buffer condition. Error bars correspond to the standard deviation (std). The largest relative standard deviation (i.e. std/mean) was ~8 %, which was used to display error bars of the SD origami results shown in **Fig. 1**k and **Supplementary Fig. 15**. It should also be noted that the buffer ion composition did not influence the photobleaching behavior of Cy3B, since both irradiance series yielded almost the same results. (**b**) Repeated irradiance series for fresh samples of TH origami under the conditions buffer L, T=21°C and [imager]=40 nM (2x samples). The plots display the mean $T_{1/2}$ values averaged over the two repeats. Error bars correspond to the standard deviation (std). The largest relative standard deviation was ~30 %, which was used to display error bars to the TH origami results shown in **Fig. 1**k, **Supplementary Fig. 15**b and **Supplementary Fig. 23**b. After filtering, SD data sets contained at least $n$~3,000 origami and TH data sets at least $n$~700 origami.

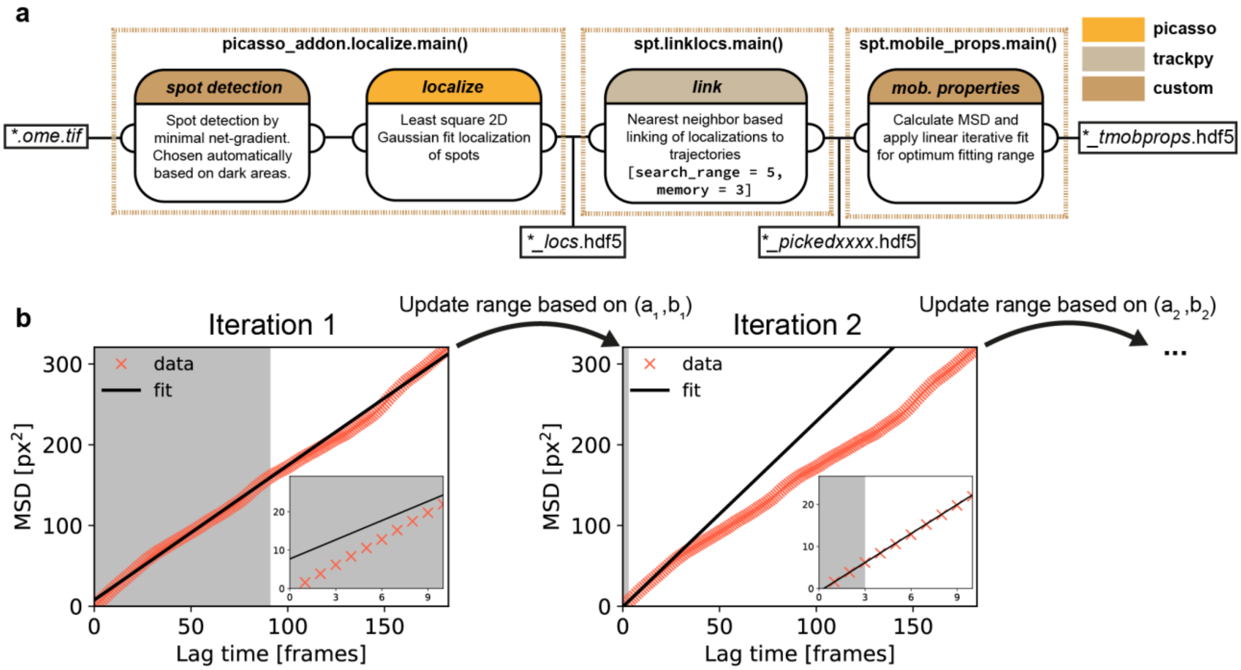


**Supplementary Figure 7. Data analysis workflow - mobile.** (**a**) Data analysis workflow from raw movies (*.ome.tif) to final result (*_tmobprops.hdf5). Dashed boxes indicate employed main functions - e.g. picasso_addon.localize.main() - of the *picasso_addon* and *spt* python package. Rounded boxes illustrate provided functionalities of each main function. The header color code indicates from which python package the functionality was adapted (e.g. *picasso, trackpy* or *custom* for extended functionalities within *picasso_addon* or *spt*). The text within the rounded boxes gives a short description of the provided functionality and parameters (brackets) for execution of the main function. For all evaluations of mobile origami a search_range value of 5 and a memory value of 3 was used (please refer to http://soft-matter.github.io/trackpy/v0.4.2/generated/trackpy.link.html#trackpy.link). The small boxes branching of the main flow represent which files are saved during execution. Please visit the links provided in the section "**Image processing & single particle tracking analysis**" for further information. Note that for all the mobile origami data presented only trajectories having more than 20 localizations were included in the analysis (**b**) We followed a linear iterative fitting procedure of the individual MSD curves as proposed by Michalet *et al.*[8] to find the optimum fitting range. For the following description we define the total trajectory length as $N$ and the maximum lag time $l$ up to which the MSD curve is fitted as $N_p$. In every step we fit the MSD with the linear fit model MSD($l$) $= a \cdot l + b$ up to $N_p$. For the first iteration we set $N_{p,1} = 0.125 \cdot N$ (nearest integer) and perform an unweighted least square fit giving $(a_1, b_1)$. The left panel shows the fitting result of the first iteration. The grey area indicates the fitting range as given by $N_{p,1}$. The zoom-in illustrates poor fitting of the MSD values for short lag times $l$ which constitute the MSD values of lowest (statistical) uncertainty[9]. For the next iteration we hence update the fitting range as given by $N_{p,2}$ using the rule[8] $N_{p,2} = 2 + 2.3(b_1/a_1)^{0.52}$ (rounded integer). The right panel shows the fitting result of the second iteration with an updated $N_{p,2}$ of 3. The zoom-in indicates that now the (low uncertainty) MSD values for short lag times $l$ are fitted well hence leading to a more precise determination of the diffusion constant. We usually observed a fast convergence in $N_p$ after 2 or 3 iterations and we only allowed up to five iterations.

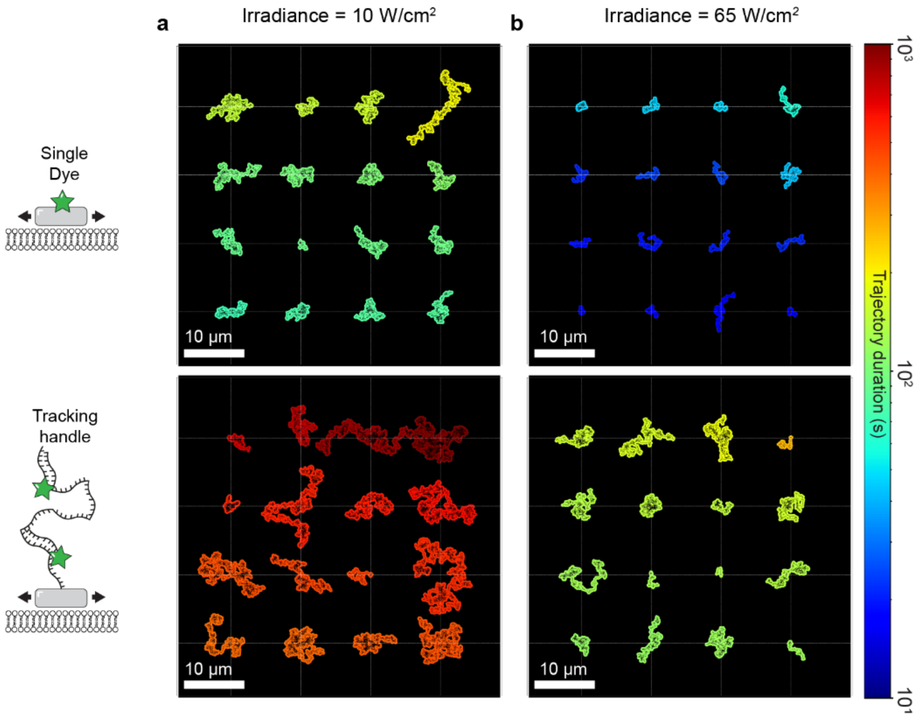


**Supplementary Figure 8. Longest trajectories at varying irradiances.** The 16 particle trajectories of longest durations of SD origami (top) and TH origami (bottom) floating on SLBs analogous to **Fig. 2**a but measured with (**a**) lower irradiance and (**b**) higher irradiance. Longest observed TH trajectory duration (top right trajectory) for an irradiance of 10 W/cm$^2$ was ~ 30 min and ~ 4 min for 65 W/cm$^2$, respectively.

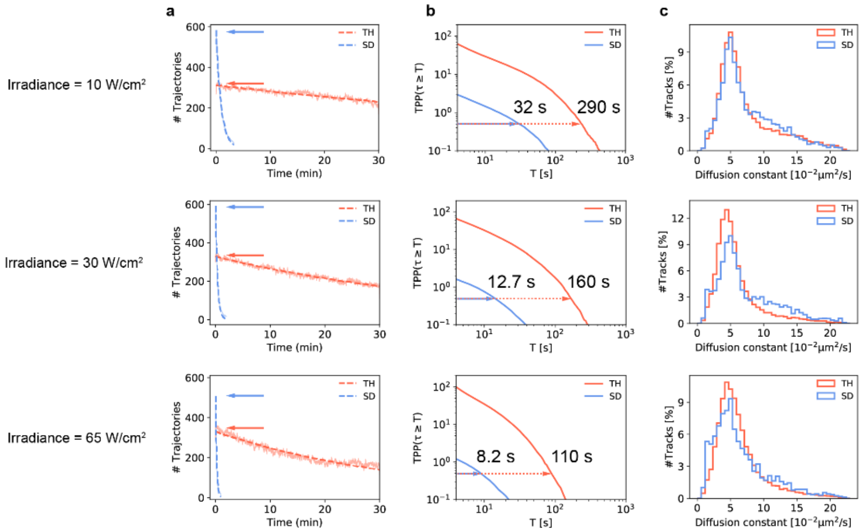


**Supplementary Figure 9. Tracks, TPP and diffusion constant at varying irradiances.** (**a**) Number of trajectories per frame analogous to **Fig. 2**b. (**b**) Average number of tracks per origami $TPP(\tau_n \geq T)$ analogous to **Fig. 2**c. (**c**) Diffusion constants as obtained by linear iterative fitting of the individual MSD curves analogous to **Fig. 2**d. Floating TH origami results are indicated by orange color, floating SD origami results are indicated by blue color. Rows represent results as obtained for varying irradiances.

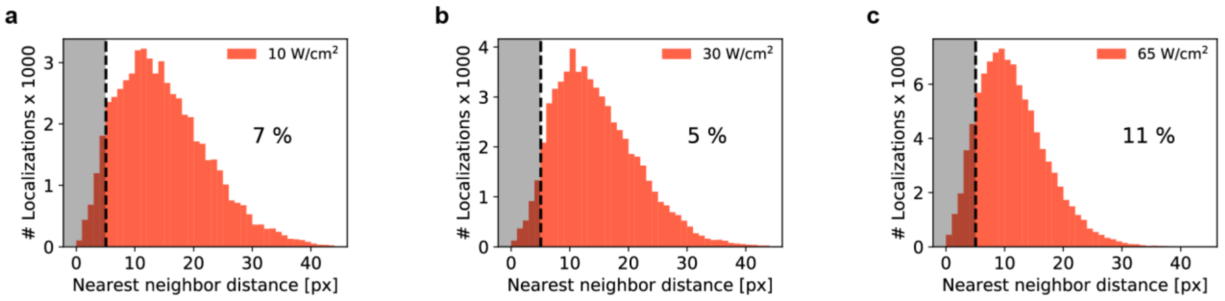


**Supplementary Figure 10. Linking and particle density.** (**a**) The data shown refers to TH origami measurements at an irradiance of 10 W/cm$^2$. It shows the nearest neighbor distance distribution between all localizations corresponding to the same frame of the recorded movie. The histogram represents the total distribution as calculated for each of the first 100 frames of the movie. The black dashed line indicates the range used as parameter for the nearest neighbor based linking algorithm ('search_range', see **Supplementary Fig. 7**). 7 % of all nearest neighbor distances between localizations of one frame lie within the search range of the linking algorithm, potentially impairing its linking ability and thus leading to trajectories of shorter duration when compared to the immobilized samples. (**b,c**) Same as (a) but at an irradiance of 30 W/cm$^2$ and 65 W/cm$^2$, respectively.

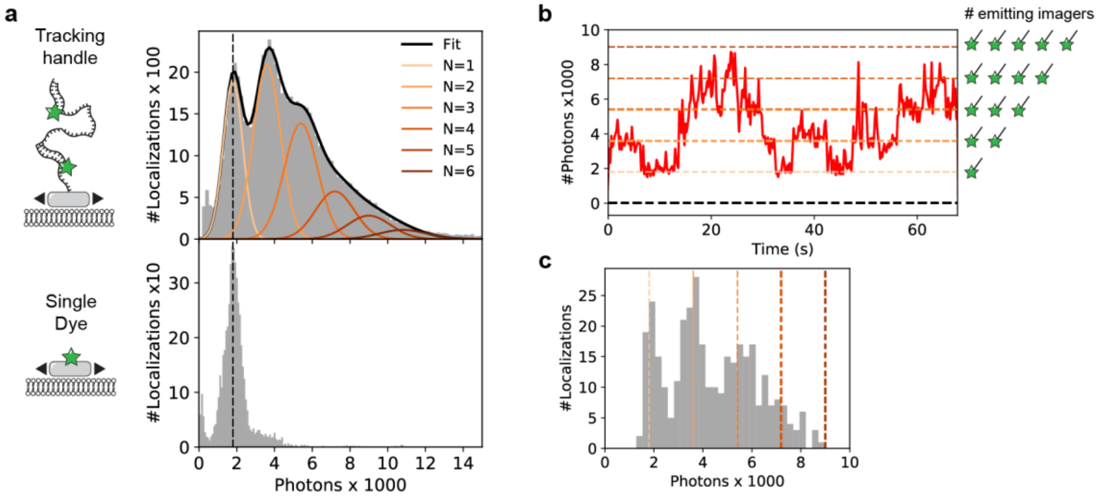


**Supplementary Figure 11. Photons counts - mobile.** (**a**) Ensemble photon count distributions as obtained from localizations corresponding to mobile TH origami (top) and SD origami (bottom) measured at irradiance = 65 W/cm$^2$. Both histograms refer to localizations from the central circular region of the FOV (diameter = 200 px) of the first 40 frames, 1000 frames of the measurement for SD origami and TH origami, respectively. Analogous to **Supplementary Fig. 5** the TH histogram was fitted with sum (solid black) of 6 Gaussian functions (colored). The dashed black line - corresponding to the center of the first peak of the TH distribution (top) - coincides with the peak of the unimodal SD origami distribution (bottom). (**b**) Fluorescence trace of an exemplary TH trajectory illustrating step-like behavior dependent on the number of emitting imagers bound to the TH at every time point. The dashed colored lines indicate the photon levels $ix_0$ as obtained from the fit of the ensemble distribution (a, top). (**c**) Photon count histogram of the trajectory shown in (b).

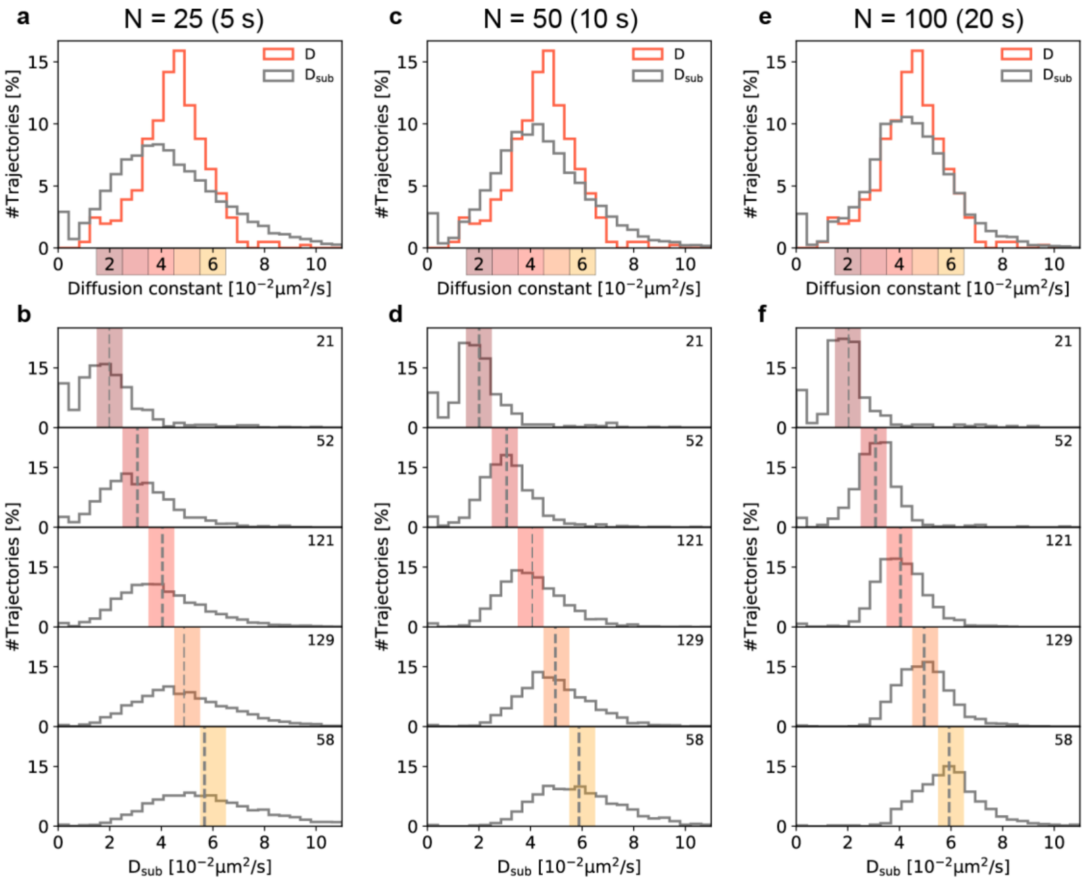


**Supplementary Figure 12. *D* vs. *D*$_{\text{sub}}$ for varying subtrajectory durations.** (**a**) Total distribution of $D$ and the corresponding subtrajectory diffusion constants $D_{\text{sub}}$ for subtrajectories of duration 5 s. We define the broadening factor $B$ of the $D_{\text{sub}}$ distribution with respect to the $D$ distribution as $B = \sigma_{\text{rel}}(D_{\text{sub}})/\sigma_{rel}(D)$ with $\sigma_{rel}$ being the relative standard deviation divided by the respective mean of the distribution and obtained a broadening factor $B$ = 1.7. (**b**) We selected five subsets of trajectories yielding a value of $D$ within the ranges 1.5 - 2.5, …, 5.5 – 6.5 $10^{-2}$ µm$^2$/s (colored boxes in a) and plotted the corresponding $D_{sub}$ distribution. The mean value of $D_{sub}$ (grey dashed) agrees well with the selected central $D$. The top right number indicates how many (full) trajectories were part of the selected subsets in $D$. The same calculus as in (a) for every subset yielded an average broadening factor $B$ = 6.1 (theoretical: 6.7, see **Supplementary Note 3**). (**c**) Same as (a) but with a subtrajectory duration of 10 s yielding broadening factor $B$ = 1.5. (**d**) Same as (b) but with a subtrajectory duration of 10 s yielding subset average broadening factor of $B$ = 4.9 (theoretical: 4.9). (**e**) Same as (a) but with a subtrajectory duration of 20 s yielding broadening factor $B$ = 1.4. (**f**) Same as (b) but with a subtrajectory duration of 20 s yielding subset average broadening factor of $B$ = 4.0 (theoretical: 3.4).

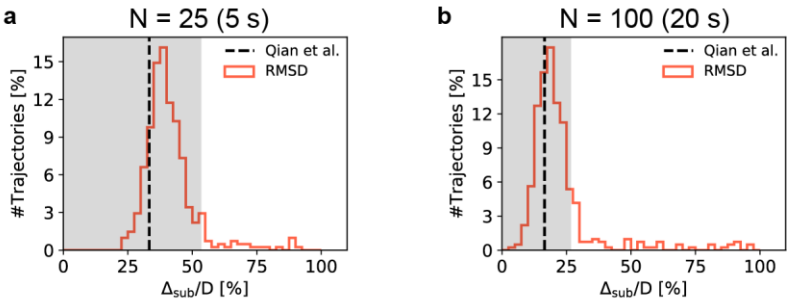


**Supplementary Figure 13. RMSD of $D_{sub}$ for varying subtrajectory durations.** (**a**) RMSD distribution of $D_{\mathrm{sub}}$ to $D$ of all trajectories exceeding 120 s split into subtrajectories of 5 s (red). RMSD was normalized to $D$ and should hence be close to the theoretical limit[9] (black dashed line) if the TH origami are subject to a time-invariant Brownian motion (see **Supplementary Note 3**). Grey area indicates deviation of less than 60 % to the theoretical limit. (**b**) Same as (a) but with a subtrajectory duration of 20 s.

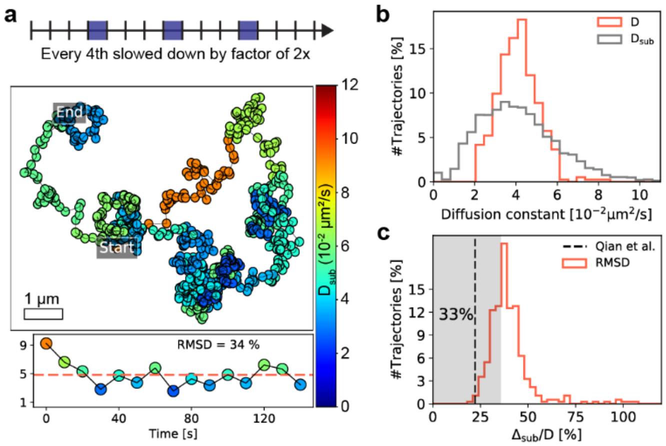


**Supplementary Figure 14. Two-fold slowdown of every fourth subtrajectory.** (**a,b,c**) same as **Fig.3**a,b,c but with sub-trajectories computationally slowed down as indicated in (**a**).

## Supplementary Note 1: Tracking handle key parameters

We investigated the various factors determining the function and performance of the TH under ideal surface-immobilized conditions. The four main kinetic rates that control continuous imager exchange are: i) the rate of photobleaching $k_{\text{photobleaching}}$, ii) the rate of photo-induced damage $k_{\text{photodamage}}$, iii) the effective imager association rate $k_{\text{on}}$ and iv) the dissociation rate $k_{\text{off}}$ (**Supplementary Fig. 15**a). Depending on the experimental conditions, such as the temperature or the imaging buffer composition as well as the excitation laser power (irradiance) at which imaging is performed, either of these rates can play a more or less dominant role for the TH. We optimized the experimental conditions for SPT experiments in a live-cell compatible buffer L (see **Supplementary Methods**), at temperature T=21 °C and at an imager concentration of 40 nM (if data was acquired at deviating conditions this is explicitly stated).

First, we examined to what extent photobleaching as a function of irradiance is a limiting factor to the TH performance. To do so, we first had to assay the average bleaching rate of single Cy3B dye molecules (remember that each imager carries a single Cy3B molecule, i.e. the average time it takes a dye molecule to photobleach should be larger than the average binding time or $k_{\text{off}} > k_{\text{photobleach}}$) by imaging immobilized SD origami at increasing irradiances ($E$=10 W/cm$^2$, 30 W/cm$^2$ and 65 W/cm$^2$). Faster photobleaching of Cy3B molecules was observed with increasing irradiance, as one would expect ($T_{1/2}$ = 30 s, 12 s and 6 s, respectively blue decaying curve in the left panel in **Supplementary Fig. 15**b). Next, we imaged TH origami at the same irradiances. Despite a similar decay with increasing irradiance, we on average obtained a 26-fold increase in $T_{1/2}$ compared to SD origami (orange curve in **Supplementary Fig. 15**b, left panel). For instance, in 30 minutes imaging of TH origami at 30 W/cm$^2$, we obtained $T_{1/2}$ of 365 s (> 6 minutes) compared to only 12 s for SD origami.

In order to suppress fast photobleaching, we repeated the irradiance series in the presence of the oxygen scavenging system POC (pyranose oxidase, catalase and glucose) and the triplet state quencher trolox (imaging buffer POCT, acquisition length: 30 min for SD origami and at least 60 min for TH origami). To our surprise, even for SD origami we obtained $T_{1/2}$ values in the range of hundreds of seconds (blue curve in **Supplementary Fig. 15**b, right panel). However, for TH origami we even obtained another 5-fold increase in comparison to SD origami (orange curve). With POCT, in 60 minutes imaging at $E$=30 W/cm$^2$, we obtained a $T_{1/2}$ of $\sim$26 minutes. Repeating the measurement at $E$=10 W/cm$^2$ with an extended measurement time of 3 hours we even obtained a $T_{1/2}$ value of more than 1 hour (see also **Supplementary Fig. 16**).

We also investigated the effect of photo-induced damage to the survival time of the TH. Analyzing the number of trajectories per frame for the TH data sets of the irradiance series clearly indicates that $k_{\text{photodamage}}$ increases with higher irradiances (**Supplementary Fig. 15**c, left panel). In **Supplementary Fig. 17** we show that $k_{\text{photodamage}}$ follows a linear dependence on the irradiance. However, despite this damage occurring over time, even at $E$=65 W/cm$^2$ (where SD origami had a $T_{1/2}$ of 6 s) on average more than 60 % of all THs were detected in every frame over 30 minutes. The source of the damage lies in reactive oxygen species, confirming previous results[1,3,10] (**Supplementary Fig. 15**c, right panel). These can be efficiently

removed using POCT maintaining a constant level of detected THs independent of the applied irradiance over the same time interval. As previously mentioned, we optimized the TH performance in a buffer compatible with live cell conditions (buffer L, T=21 °C, 40 nM imager). For future specific SPT problems, one or more of these conditions might have to be adapted. In the following, we therefore analyzed the effect of changes to imager concentration, temperature and buffer composition (salt), keeping the other parameters fixed, with respect to TH function.

First, we varied the imager concentration for samples with immobilized TH origami ([imager] = 5 nM, 10 nM, 20 nM and 40 nM, at T=21°C, buffer L). As one would expect, increasing the imager concentration resulted in an increase in trajectory durations due to a higher probability of binding to an unoccupied site on the TH (**Supplementary Fig. 15**d, top left panel). However, increasing from 20 to 40 nM imager concentration, the increase in $T_{1/2}$ is close to saturation, particularly at high irradiances. Looking at the number of photons detected per localization (**Supplementary Fig. 15**d, bottom left panel), we similarly observed an increase with imager concentration because multiple emitting imagers simultaneously bind the TH. The corresponding distributions of photon counts illustrates how the probability of higher order occupancies of bound imagers increases with concentration (**Supplementary Fig. 15**d, right panel).

Third, we highlight the influence of temperature on TH performance. Increasing the temperature from 21 °C to 23 °C (fix: buffer L, 40 nM imager concentration) had a large effect on the average trajectory duration despite this small temperature difference, as $T_{1/2}$ dropped on average by $\sim$30 % (**Supplementary Fig. 15**e). The drop is due to an increased $k_{\mathrm{off}}$, which results in faster imager dissociation and thus more frequent interruptions in the fluorescence signal (**Supplementary Fig. 18**). Lastly, we emphasize the effect of ion composition (salt) onto the TH. We performed the same irradiance series for TH origami samples using two buffers with different ion compositions: buffer L (3 mM $MgCl_2$ + 140 mM NaCl) and buffer B (10 mM $MgCl_2$) at T=21 °C and 5 nM imager concentration. The buffer with the higher amount of $Mg^{2+}$ ions, buffer B, resulted in longer trajectories by $\sim$40 % compared to buffer L due to the beneficial effect of higher $MgCl_2$ concentrations to both $k_{\mathrm{on}}$ and $k_{\mathrm{off}}$ (**Supplementary Fig. 15**f, **Supplementary Fig. 19**).

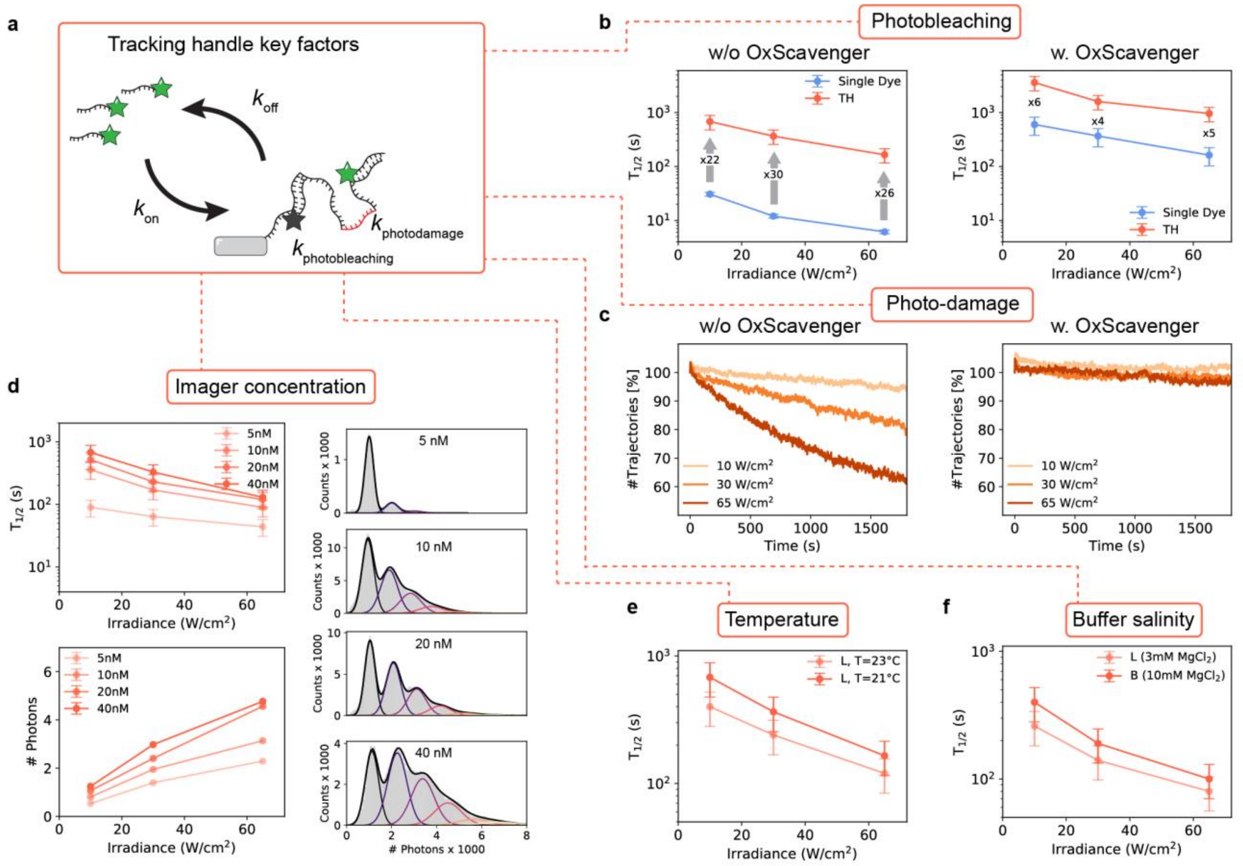


**Supplementary Figure 15. Tracking handle key parameters overview.** (**a**) Main rates determining the TH function (**b**) Mean $T_{1/2}$ vs. irradiance plots for SD origami (blue) and TH origami (orange) imaged at varying irradiances. Left and right panels show the case without and with using an oxygen scavenging system, respectively. Arrows indicates factors of increase of TH vs SD. SD data sets contained at least $n$~3,000 origami and TH data sets at least $n$~700 origami after filtering. (**c**) Number of trajectories per frame (i.e. emitting labels) vs. measurement time normalized to initial trajectory number. Left and right panels show the case without (a total of at least $n$~109.000 trajectories per data set) and with using an oxygen scavenging system (a total of at least $n$~241.000 trajectories per data set), respectively. (**d**) Top left panel: Mean $T_{1/2}$ vs. irradiance plot for TH origami samples imaged at varying imager concentrations. Bottom left panel: Plot of mean number of detected photons per localization. Right panels: Distributions of number of photons detected per localization corresponding to concentration series imaged at *E*=30 W/cm$^2$. All data sets contained at least $n$~400 origami after filtering. (**e**) Mean $T_{1/2}$ vs. irradiance plots for TH origami imaged at varying temperature (All data sets contained at least $n$~2,300 origami after filtering). (**f**) Mean $T_{1/2}$ vs. irradiance plots for TH origami imaged at varying buffer ion compositions (All data sets contained at least $n$~1,400 origami after filtering). Error bars in b,c,d,e,f correspond to relative standard deviation (see **Supplementary Fig. 6)**.

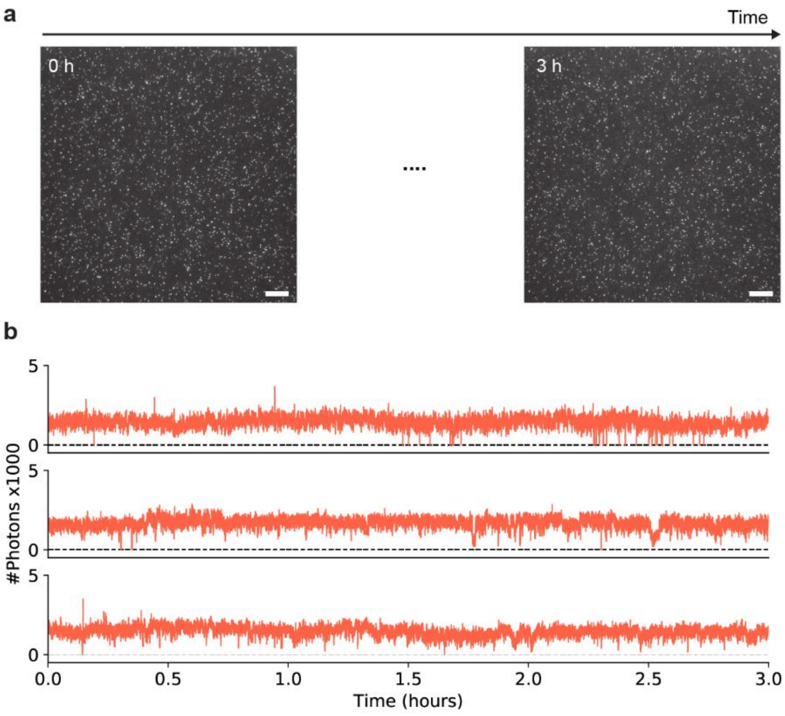


**Supplementary Figure 16. Extended 3-hour measurement using POCT.** (**a**) First and last frame of the 3h TIRFM acquisition of static TH origami still emitting after the course of the measurement. Parameters: buffer POCT, [imager]=40 nM, T=21 °C, $E$=10 W/cm$^2$. (**b**) Three exemplary fluorescence traces exhibiting continuous intensity fluctuations over 3 h. The combination POC and trolox suppresses photobleaching and photodamage to the TH. However, still short interruptions due to the stochastic nature of DNA association and dissociation occur. Scale bars, 10 $\mu$m in (a).

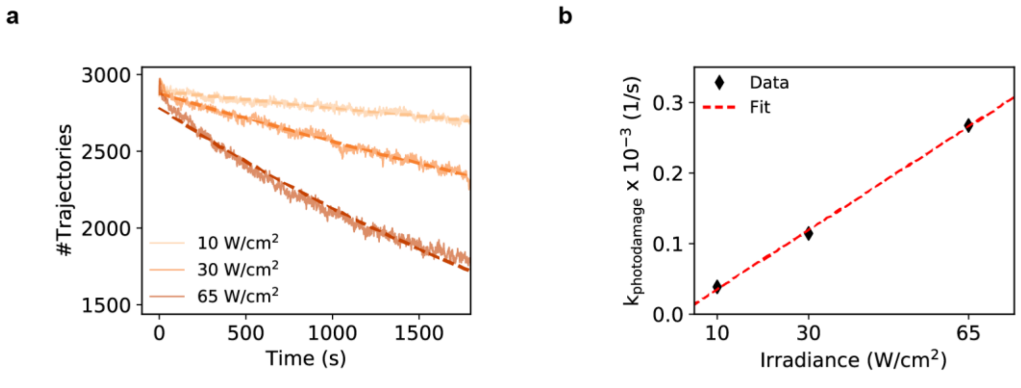


**Supplementary Figure 17. $k_{\text{photodamage}}$ vs irradiance.** (**a**) Number of trajectories per frame vs. measurement time normalized to initial trajectory number (transparent fluctuating curves) from DNA origami samples imaged at varying irradiances, as shown in left panel in **Supplementary Fig. 15**c. An exponential model $f(x) = ae^{-x/\tau_{\text{photodamage}}}$ was fitted to the data (dashed curves), where $\tau_{\text{photodamage}}$ denotes the characteristic decay constant over which photodamage occurs and $a$ the initial number of trajectories. (**b**) The inverse of $\tau_{\text{photodamage}}$ yielded the rate $k_{\text{photodamage}} = 1/\tau_{\text{photodamage}}$ for each irradiance. Plotting $k_{\text{photodamage}}$ vs. irradiance was well described by a line fit (red dashed line) confirming a linear relation of the two quantities over the here applied irradiance range.

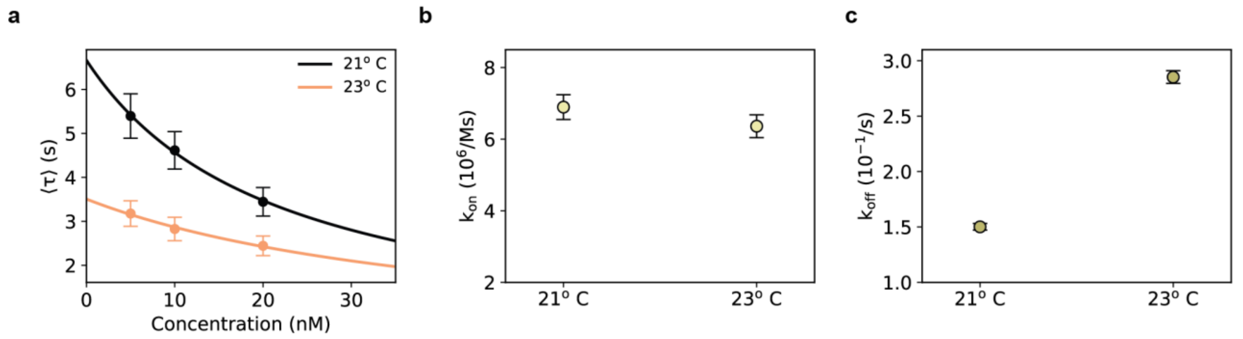


**Supplementary Figure 18. Temperature effect on DNA hybridization rates.** (**a**) As previously described[3], we performed localization-based Fluorescence Correlation Spectroscopy (lbFCS) measurements on DNA origami labeled with just a single docking strand fully complementary to the imager sequence (see **Supplementary Table 2**). By performing an imager concentration series ([imager]=5 nM, 10 nM and 20 nM) lbFCS allows to precisely measure the DNA hybridization rates $k_{on}$ and $k_{off}$[3]. Here, we conducted the imager concentration series in buffer B at T=21 °C and T=23 °C. Fitting eq. 2 in ref. 3 to the mean characteristic decay constant ⟨τ⟩ plotted vs. the imager concentration yielded $k_{on}$ and $k_{off}$. (**b**) $k_{on}$ results from the two fits in (a) indicating only minor changes due to the temperature variation. (**c**) $k_{off}$ results from the two fits in (a) highlighting the drastic temperature effect on the dissociation rate. $k_{off}$ increased almost by a factor of x2 when increasing the temperature from 21 °C to 23 °C. Error bars correspond to standard deviation in (a) and to the lbFCS uncertainty range[3] of 5 % and 2 % in $k_{on}$ and $k_{off}$, respectively, in (b). All data sets contained at least $n \sim 5{,}500$ origami after filtering.

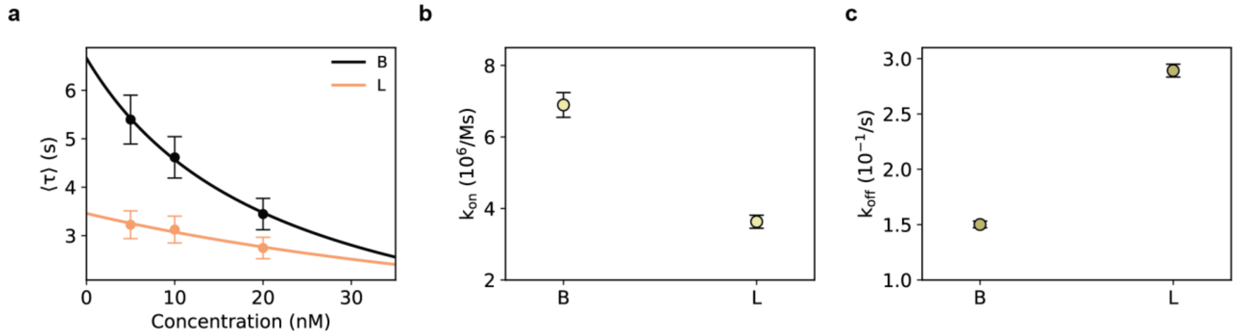


**Supplementary Figure 19. Ion concentration effect on DNA hybridization rates.** (**a**) Analogously to **Supplementary Fig. 18**, we repeated the same lbFCS imager concentration series on single-docking-strand origami samples using buffer B and buffer L (at T=21 °C). (**b**) $k_{on}$ results from the two fits in (a) exhibiting a decreased association rate due to a lower $MgCl_2$ concentration in buffer L. (**c**) $k_{off}$ results from the two fits in (a). In contrast to $k_{on}$, $k_{off}$ increased nearly by a factor of 2x using buffer L compared to buffer B. Error bars correspond to standard deviation in (a) and to the lbFCS uncertainty range[3] of 5 % and 2 % in $k_{on}$ and $k_{off}$, respectively, in (b). All data sets contained at least $n \sim 535$ origami after filtering.

## Supplementary Note 2: TPP calculus for mobile particles

Since in SPT, it is generally not possible to unambiguously identify multiple appearances of the same mobile particle in a data set, we calculated $TPP(\tau_n \geq T) = \frac{N_{\text{tot}}(\tau_n \geq T)}{M}$ for trajectories with duration $\tau_n$ longer or equal to the time $T$ by normalizing the total number of trajectories $N_{\text{tot}}(\tau_n \geq T)$ to the initial trajectory number $M$ (arrows in **Fig. 2**b) in order to directly compare trajectory durations of both immobile and mobile origami. Note that $M$ should give a good estimate for the number of particles within the FOV for sufficiently low particle densities. $TPP(\tau_n \geq T)$ was $< 1.5$ for the SD origami, confirming the validity of our approach since we ideally would expect only one trajectory per single dye before it photobleaches (see **Fig. 2**c). To test the applicability of this approach we reanalyzed the immobilized TH origami using this procedure and obtained almost identical results as for the immobilized origami analysis workflow (**Supplementary Fig. 20**).

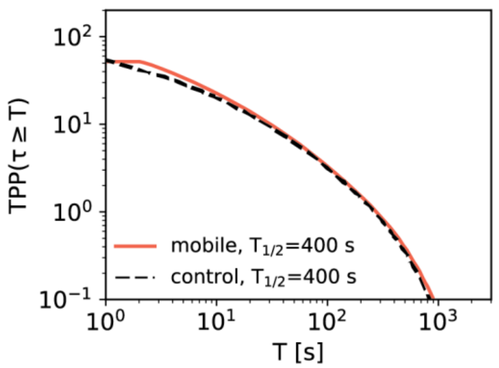


**Supplementary Figure 20. Mobile analysis control with immobilized data set.** In order to verify our approach of TPP calculation for the mobile case (see **Fig. 2**b,c) we reanalyzed an immobilized TH origami sample using the mobile analysis workflow described in **Supplementary Fig. 7**. The orange curve displays the corresponding TPP results. The black dashed line displays the TPP results as obtained via the immobile analysis workflow described in **Supplementary Fig. 3** and serves as a control**.** Both curves evolve almost identically and yield the same average TPP of ~50 and a $T_{1/2}$ value of 400 s. This confirms our approach for the mobile case to normalize the total number of detected trajectories to the number of trajectories (i.e. particles) in the first frame of the data set.

## Supplementary Note 3: Statistical treatment of diffusion coefficients

A theoretical expression for the relative standard deviation of the diffusion coefficient as obtained by fitting of the MSD curve was derived by Qian et al.[9]:

$$\frac{\sigma_D}{D} = \sqrt{\frac{2N_p}{3(N - N_p)}} \qquad \text{Eq. (1)}$$

with $N$ being the total duration of the trajectory in frames (e.g. $N$ = 600 for full trajectories, $N$ = 50 for subtrajectories in **Fig. 3**) and $N_p$ being the maximum lag time (in frames) up to which the MSD curve was fitted (see **Supplementary Fig. 7**).

In our case $N_p$ was chosen automatically during the iterative fitting process[8] resulting in a median value of $N_p = 3$ on average. To test whether the scatter of $D_{\text{sub}}$ is only governed by the random nature of the motion according to Eq. 1, we calculated the root-mean-square deviation $\text{RMSD} = \sqrt{\langle (D_{\text{sub}} - D)^2 \rangle}$ of $D_{\text{sub}}$ to $D$ for each trajectory[11]. **Fig. 3**c shows the distribution of the thus obtained RMSD normalized to the diffusion coefficient $D$ of the full trajectory for $N$ = 50. If the movement of the TH origami is indeed governed by the same 2D Brownian motion with a diffusion coefficient $D$ at any time the RMSD should correspond to the standard deviation $\sigma_D$ (for $N$ = 50) and should hence yield a value close to theoretically achievable limit given by Eq. 1 (black dashed line, in **Fig. 3**c). **Fig. 3**c shows that around 86 % of the analyzed trajectories do not deviate from the expected statistical uncertainty by more than 60 % (grey area) suggesting that the TH origami are indeed subject to a time-invariant Brownian motion on the SLB. This statement is further supported by division into longer ($N$ = 100) or shorter ($N$ = 25) subtrajectories (see **Supplementary Fig. 13**).

## Supplementary Note 4: Potential limitations and workarounds

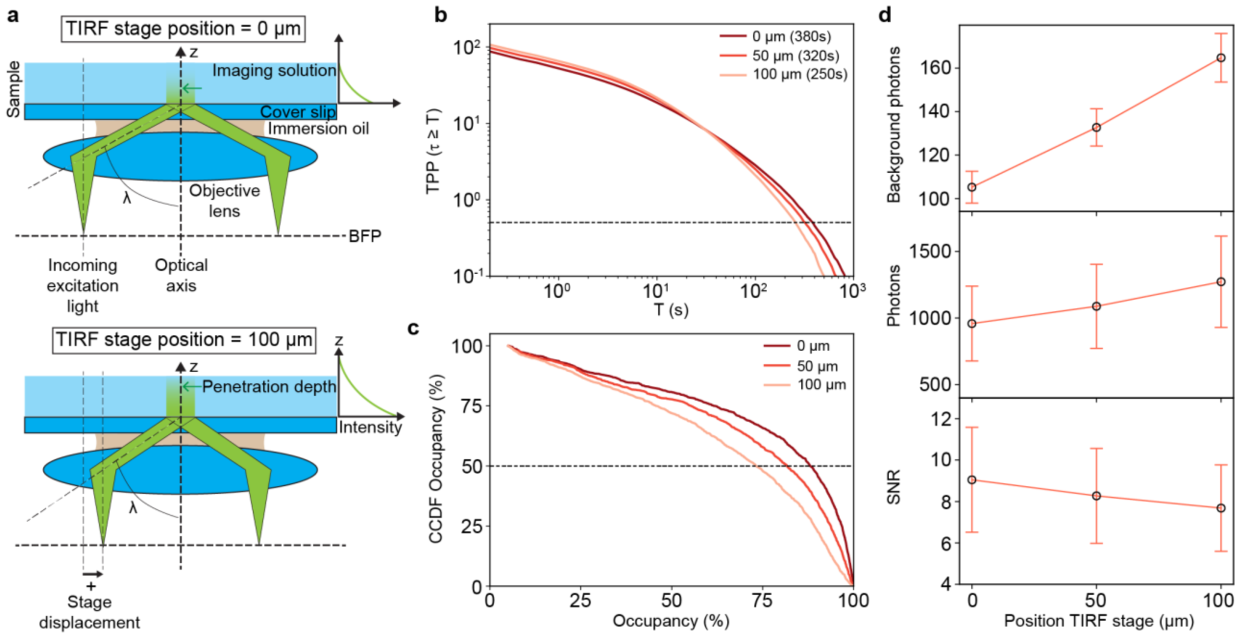


**Supplementary Figure 21. Performance of tracking handle at varying TIRF angles**. (**a**) Sketch to illustrate the variation of the TIRF angle λ defined as the angle of incidence of the excitation light with respect to the optical axis. In order to vary λ, a linear stage bearing the coupling lens focusing the laser into the back focal plane (BFP) of the objective was moved to laterally decrease the offset from the optical axis within the BFP. In our case, a positive displacement of the TIRF stage (bottom) with respect to the zero position (top) resulted in a decrease of λ. According to literature[12], a decrease in λ leads to an increasing penetration depth of the resulting evanescent field and hence a larger excitation volume (indicated by green arrows). Additionally, a decrease in λ results in higher excitation intensities (relative to the incoming light) of the resulting evanescent field (indicated by intensity vs. z plots to the right). (**b**) Plot of $TPP(\tau \geq T)$ vs. $T$ for immobilized TH origami acquired at different stage displacements (i.e., varying λ). We observed shorter $T_{1/2}$-values (given in brackets in the legend) with increased displacements (i.e., decreased λ) indicating a higher irradiance (compare **Supplementary Fig. 15** and see **Supplementary Table 1** for imaging conditions). (**c**) As explained in **Supplementary Fig. 4**, we calculated the occupancy for immobilized TH origami. The plot shows the complementary cumulative distribution function (CCDF=1-CDF) of the calculated occupancies for the three data sets in (a). As expected from (a), the occupancy decreases with increasing TIRF angle. (**d**) Top: Plot of mean registered background photons per localization vs. TIRF stage position for the three data sets in (b-c). A larger displacement (i.e., a smaller λ) results in higher background due to the increased excitation volume within the non-fluorogenic imaging solution. Middle: Plot of mean photons detected per localization vs. TIRF stage position also showing an increase with larger displacements (i.e., smaller λ), indicating an increased intensity of the evanescent (excitation) field. Bottom: Plot of signal-to-noise ratio (SNR) vs. TIRF stage position. Overall, the SNR (detected TH photons/background photons) decreases due to the more pronounced background with larger displacements (i.e., smaller λ). All data sets contained at least $n$~1,300 origami after filtering. Error bars in d correspond to standard deviation.

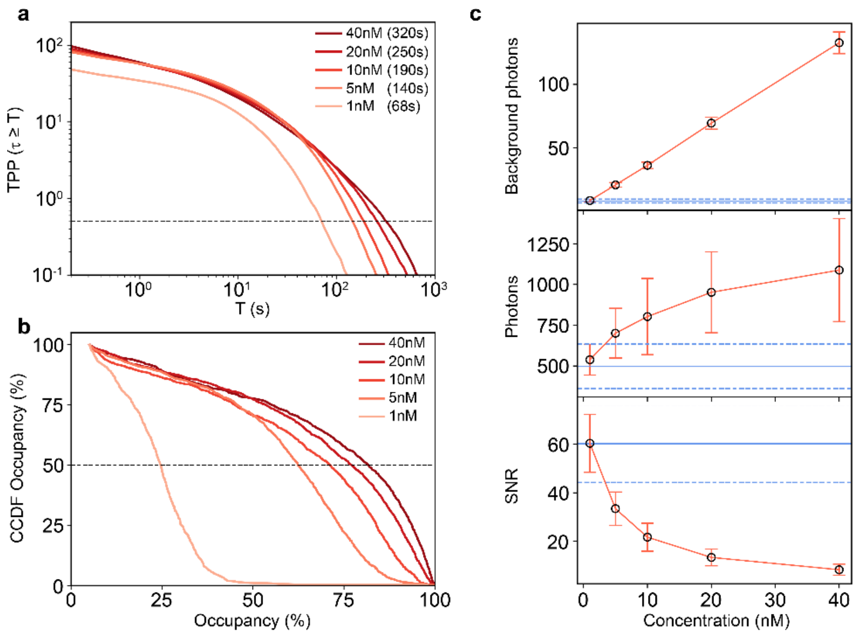


**Supplementary Figure 22. Performance of tracking handle at varying imager concentrations**. (**a**) Plot of $TPP(\tau \geq T)$ vs. $T$ for immobilized TH origami acquired at different imager concentrations. As expected we observed shorter $T_{1/2}$-values (given in brackets in the legend) for decreasing imager concentration. (**b**) Complementary cumulative distribution function (CCDF) of occupancies for immobilized TH origami acquired at different imager concentrations (equivalent to **Supplementary Fig. 21**, b). (**c**) Top: Plot of mean registered background photons per localization vs. imager concentration for the data shown in (a-b) indicating linear response of the background fluorescence vs. imager concentration. The blue line indicates the measured value for SD origami for the same imaging conditions. Middle: Plot of mean photons detected per localization vs. imager concentration indicating saturation behavior for higher imager concentrations. Bottom: Plot of signal-to-noise ratio (SNR) vs. imager concentration. Overall, the SNR (detected TH photons/background photons) decreases due to the more pronounced background with higher imager concentrations. All data sets contained at least $n$~460 origami after filtering. Error bars and dashed blue lines in c correspond to relative standard deviation.

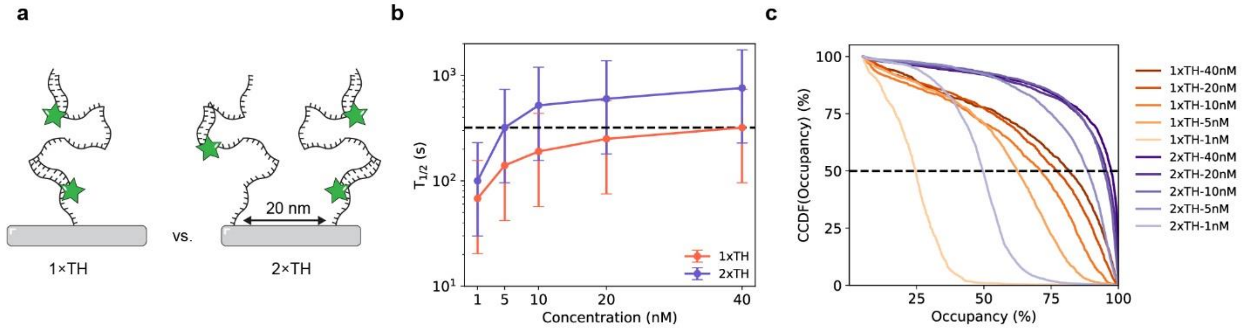


**Supplementary Figure 23**. **2×TH vs. 1×TH labeling.** (**a**) Standard TH origami design with a single TH (1×TH) vs. origami featuring two TH labels at a 20 nm spacing (2×TH). (**b**) Mean $T_{1/2}$ vs. imager concentration for immobilized 1×TH origami (standard, orange) and 2×TH origami (purple). Labeling with two THs dramatically increases observation times. The black dashed line highlights that using an imager concentration of 5 nM with 2×TH origami yields a similar $T_{1/2}$ compared to 1×TH origami at an imager concentration of 40 nM, hence, allowing an 8-fold reduction in imager concentration. (**c**) Complementary cumulative distribution functions (CCDF) of the occupancy for 1×TH origami (orange shades) and 2×TH (purple shades) for the same data sets as in (b). All data sets contained at least $n$~430 origami after filtering. Error bars in b correspond to relative standard deviation (see **Supplementary Fig. 6**).

# Supplementary Tables

## Supplementary Table 1 | Imaging parameters

| Figure | Sample | Imager concentration (nM) | Imaging Buffer | Temperature (°C) | Irradiance (W/cm$^2$) | Frames (wrt. Irradiance) |
|---|---|---|---|---|---|---|
| 1c,f,h,j,k<br>SI_Fig. 5 | SD origami, static | - | L | 21 | 30 | 600 |
| 1d,g,i,j,k<br>SI_Figs. 5,20 | TH origami, static | 40 | L | 21 | 30 | 9,000 |
| 2<br>SI_Figs. 9 | SD origami, diffusing on SLB | - | L | 21 | 30 | 600 (3x FOVs) |
| 2<br>3<br>SI_Figs. 9,10,12,13,14 | TH origami, diffusing on SLB | 40 | L | 21 | 30 | 9,000 |
| SI_Fig 6a | SD origami, static | - | B (4x samples), L (1x sample) | 21 | 10<br>30<br>65 | 2,000 (3x FOVs)<br>600 (3x FOVs)<br>300 (3x FOVs) |
| SI_Fig 6b | TH origami, static | 40 | L (2x samples) | 21 | 10<br>30<br>65 | 9,000<br>9,000<br>9,000 |
| SI_Figs. 8,9,11 | SD origami, diffusing on SLB | - | L | 21 | 10<br>65 | 1,000 (3x FOVs)<br>300 (3x FOVs) |
| SI_Figs. 8,9,10,11 | TH origami, diffusing on SLB | 40 | L | 21 | 10<br>65 | 9,000<br>9,000 |
| SI_Fig 15b, left panel | SD origami, static | - | L | 21 | 10<br>30<br>65 | 2,000 (3x FOVs)<br>600 (3x FOVs)<br>300 (3x FOVs) |
| SI_Fig 15b, left panel<br>SI_Fig 15c, left panel<br>SI_Fig 17 | TH origami, static | 40 | L | 21 | 10<br>30<br>65 | 9,000<br>9,000<br>9,000 |

| Figure | Sample | Imager concentration (nM) | Imaging Buffer | Temperature (°C) | Irradiance ($W/cm^2$) | Frames (wrt Irradiance) |
|---|---|---|---|---|---|---|
| SI_Fig 15b, right panel | SD origami, static | - | POCT | 21 | 10<br>30<br>65 | 9,000<br>9,000<br>9,000 |
| SI_Fig 15b, right panel<br>SI_Fig 15c, right panel | TH origami, static | 40 | POCT | 21 | 10<br>30<br>65 | 18,000<br>18,000<br>18,000 |
| SI_Fig. 15d | TH origami, static | 5, 10, 20, 40<br>(4 samples) | L | 21 | 10<br>30<br>65 | 9,000<br>9,000<br>9,000 |
| SI_Fig. 15e | TH origami, static | 40 nM | L | 21, 23 | 10<br>30<br>65 | 9,000<br>9,000<br>9,000 |
| SI_Fig. 15f | TH origami, static | 5 nM | L, B<br>(2 samples) | 21 | 10<br>30<br>65 | 9,000<br>9,000<br>9,000 |
| SI_Fig 16 | TH origami, static | 40 | POCT | 21 | 10 | 54,000 |
| SI_Fig. 18 | 1DS origami | 5, 10, 20<br>(3 samples) | B | 21, 23 | 10 | 9,000<br>(6x) |
| SI_Fig. 19 | 1DS origami | 5, 10, 20 | B, L<br>(2x3=6 samples) | 21 | 10 | 9,000<br>(6x) |
| SI_Fig. 21 | TH origami, static | 40 | L | 21 | 30<br>(varying TIRF angles) | 9000 (3x) |
| SI_Fig. 22 | TH origami, static | 1,5,10,20,40 | L | 21 | 30 | 9000 (5x) |
| SI_Fig. 23 | TH & 2xTH origami, static | 1,5,10,20,40<br>(TH & 2xTH) | L | 21 | 30 | 9000 (10x) |

**Supplementary Table 2 | Used DNA oligonucleotide sequences as labels**

| **Name (oligo length)** | **Docking strand sequence (5' – 3')** | **Imager sequence (5' – 3')** | **Experiment** |
|---|---|---|---|
| TH (54 bp) | TT-CTCCTCCTCCTCCTCCTCCTCCTCCTCCTCCTCCTCCTCCTCCTCCTCCTCCTC | GAGGAGGA-Cy3B | All TH experiments |
| SD (5 bp) | TT TTT-Cy3B | - | All SD experiments |
| 1DS (8 nt) | TT TCCTCCTC | GAGGAGGA-Cy3B | lbFCS series in SI_Fig. 18 & 19 |

## Supplementary References


1. Blumhardt, P. *et al.* Photo-Induced Depletion of Binding Sites in DNA-PAINT Microscopy. *Molecules* **23**, 3165 (2018).
2. Schnitzbauer, J., Strauss, M. T., Schlichthaerle, T., Schueder, F. & Jungmann, R. Super-resolution microscopy with DNA-PAINT. *Nat. Protoc.* **12**, 1198 (2017).
3. Stein, J. *et al.* Toward Absolute Molecular Numbers in DNA-PAINT. *Nano Lett.* **19**, 8182–8190 (2019).
4. Stahl, E., Martin, T. G., Praetorius, F. & Dietz, H. Facile and Scalable Preparation of Pure and Dense DNA Origami Solutions. *Angew. Chemie Int. Ed.* **53**, 12735–12740 (2014).
5. Stehr, F., Stein, J., Schueder, F., Schwille, P. & Jungmann, R. Flat-top TIRF illumination boosts DNA-PAINT imaging and quantification. *Nat. Commun.* **10**, 1268 (2019).
6. Edelstein, A. D. *et al.* Advanced methods of microscope control using µManager software. *J. Biol. Methods; Vol 1, No 2* (2014).
7. Endesfelder, U., Malkusch, S., Fricke, F. & Heilemann, M. A simple method to estimate the average localization precision of a single-molecule localization microscopy experiment. *Histochem. Cell Biol.* **141**, 629–638 (2014).
8. Michalet, X. Mean square displacement analysis of single-particle trajectories with localization error: Brownian motion in an isotropic medium. *Phys. Rev. E* **82**, 41914 (2010).
9. Qian, H., Sheetz, M. P. & Elson, E. L. Single particle tracking. Analysis of diffusion and flow in two-dimensional systems. *Biophys. J.* **60**, 910–921 (1991).
10. Clowsley, A. H. *et al.* Repeat DNA-PAINT suppresses background and non-specific signals in optical nanoscopy. *Nat. Commun.* **12**, 501 (2021).

11. Block, S., Zhdanov, V. P. & Höök, F. Quantification of Multivalent Interactions by Tracking Single Biological Nanoparticle Mobility on a Lipid Membrane. *Nano Lett.* **16**, 4382–4390 (2016).

12. MARTIN-FERNANDEZ, M. L., TYNAN, C. J. & WEBB, S. E. D. A ‘pocket guide’ to total internal reflection fluorescence. *J. Microsc.* **252**, 16–22 (2013).
