# Supplementary material for: Tracking single particles for hours via continuous DNA-mediated fluorophore exchange
Source: Nat Commun. 2021 Jul 21;12:4432. doi: 10.1038/s41467-021-24223-4 (PMC8295357; doi:10.1038/s41467-021-24223-4)
Supplement: Supplementary file 4 — Description of Additional Supplementary Files [file 41467_2021_24223_MOESM4_ESM.pdf]

Title: Supplementary Movie 1:

Description: Movie showing 120 seconds of raw data acquisition of SD origami undergoing 2D diffusion on SLB (one of the SD origami data sets evaluated in Fig. 2). The playback speed was increased by a factor 4x.

Title: Supplementary Movie 2:

Description: Movie showing 10 minutes of raw data acquisition of TH origami undergoing 2D diffusion on SLB (a subset of the TH origami data set evaluated in Fig. 2). The playback speed was increased by a factor 20x.
