# Supplementary figures and images for: Tracking single particles for hours via continuous DNA-mediated fluorophore exchange (part 2 of 2)
Source: Nat Commun. 2021 Jul 21;12:4432. doi: 10.1038/s41467-021-24223-4 (PMC8295357; doi:10.1038/s41467-021-24223-4)

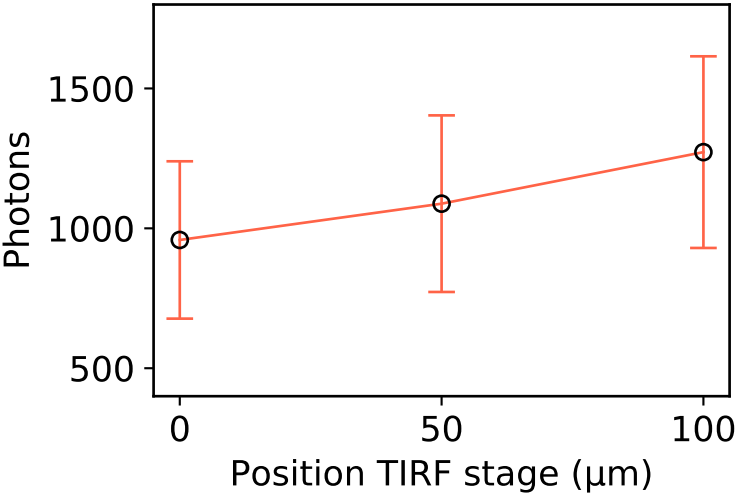

Supplement: Supplementary file 7 — Source Data [file 41467_2021_24223_MOESM7_ESM.zip › z.source-data/si/si_fig21_JS/d/plots/si_fig21d_photons.pdf]

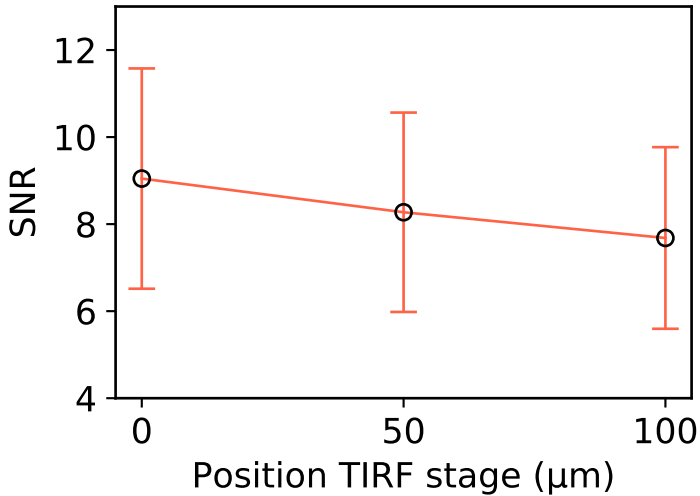

Supplement: Supplementary file 7 — Source Data [file 41467_2021_24223_MOESM7_ESM.zip › z.source-data/si/si_fig21_JS/d/plots/si_fig21d_snr.pdf]

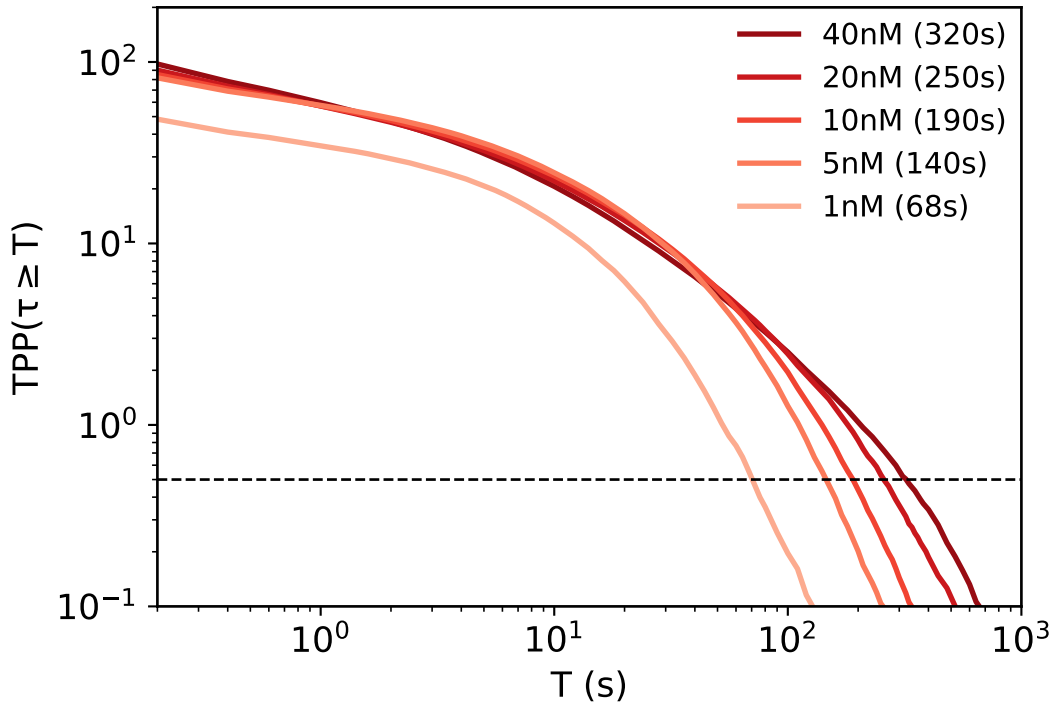

Supplement: Supplementary file 7 — Source Data [file 41467_2021_24223_MOESM7_ESM.zip › z.source-data/si/si_fig22_FS/a/plots/fig22a.pdf]

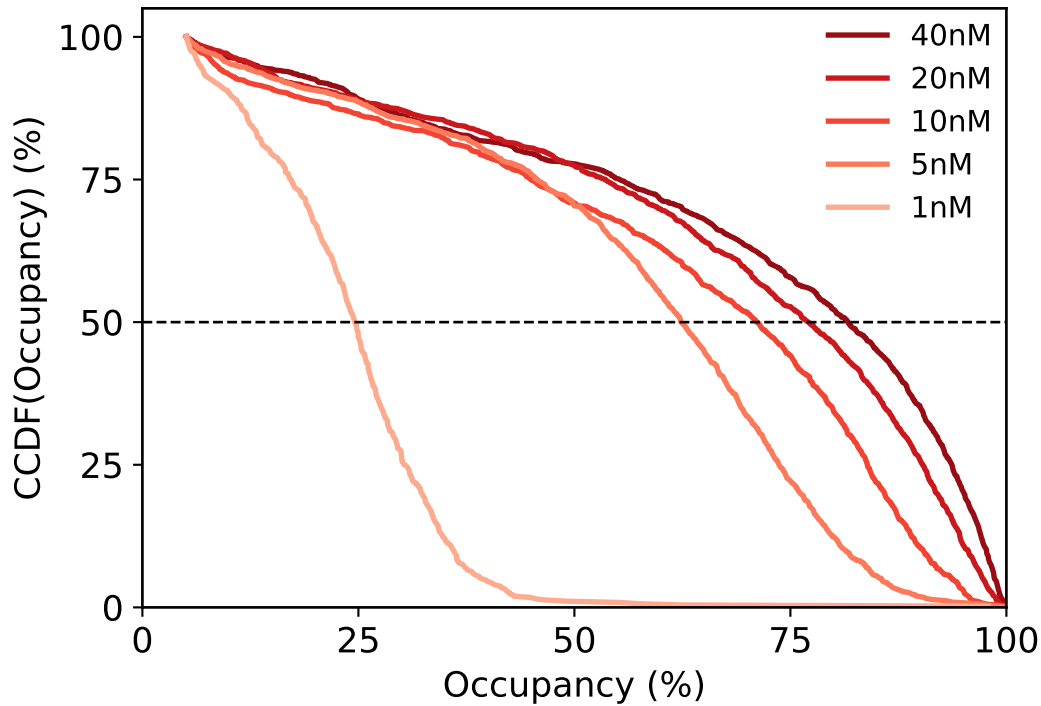

Supplement: Supplementary file 7 — Source Data [file 41467_2021_24223_MOESM7_ESM.zip › z.source-data/si/si_fig22_FS/b/plots/fig22b.pdf]

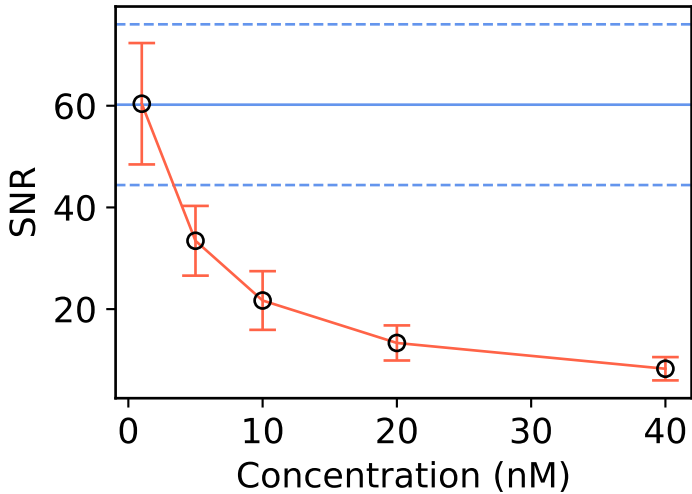

Supplement: Supplementary file 7 — Source Data [file 41467_2021_24223_MOESM7_ESM.zip › z.source-data/si/si_fig22_FS/c/plots/fig22c_lower.pdf]

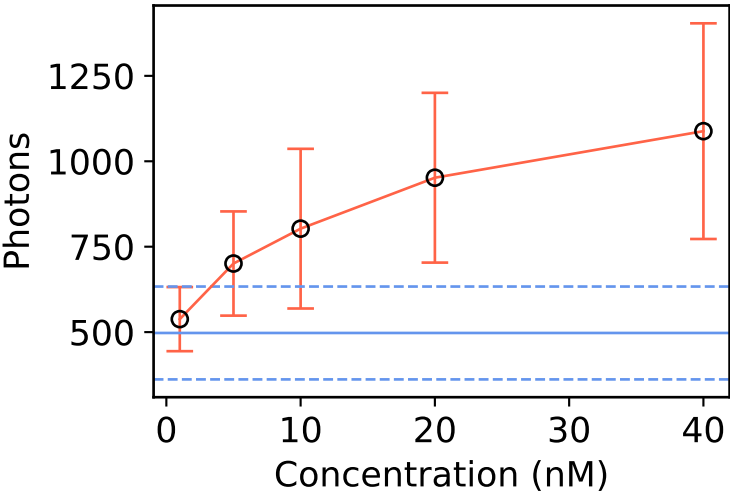

Supplement: Supplementary file 7 — Source Data [file 41467_2021_24223_MOESM7_ESM.zip › z.source-data/si/si_fig22_FS/c/plots/fig22c_middle.pdf]

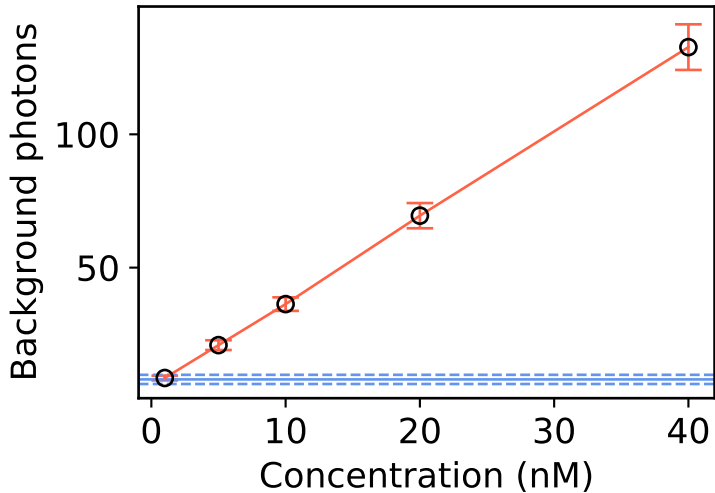

Supplement: Supplementary file 7 — Source Data [file 41467_2021_24223_MOESM7_ESM.zip › z.source-data/si/si_fig22_FS/c/plots/fig22c_upper.pdf]

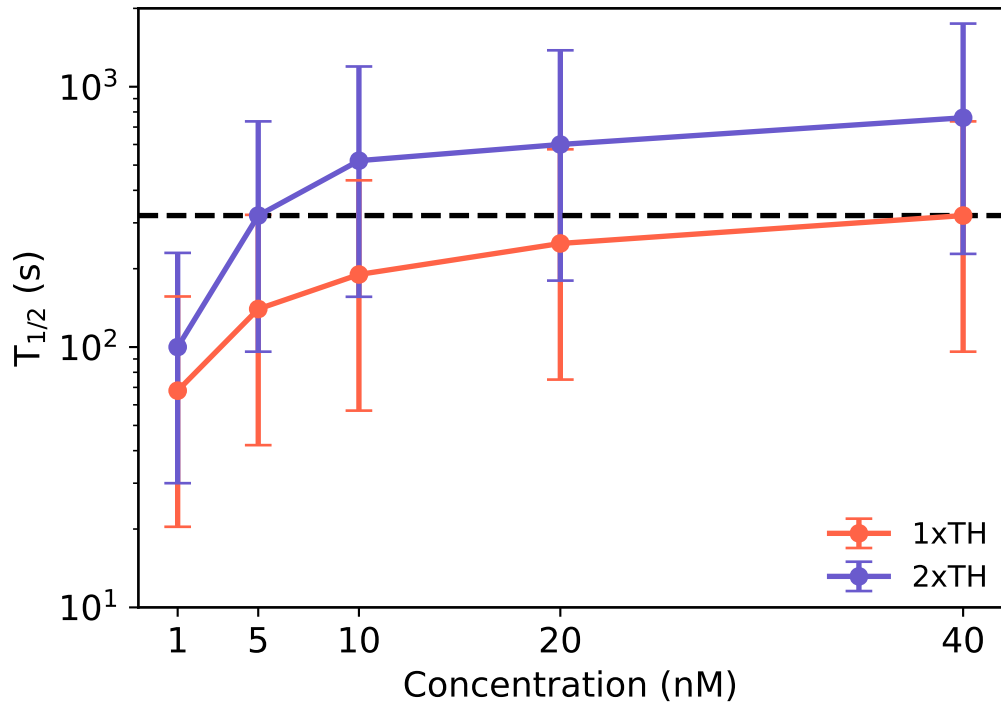

Supplement: Supplementary file 7 — Source Data [file 41467_2021_24223_MOESM7_ESM.zip › z.source-data/si/si_fig23_JS/plots/si_fig23b_TPP.pdf]

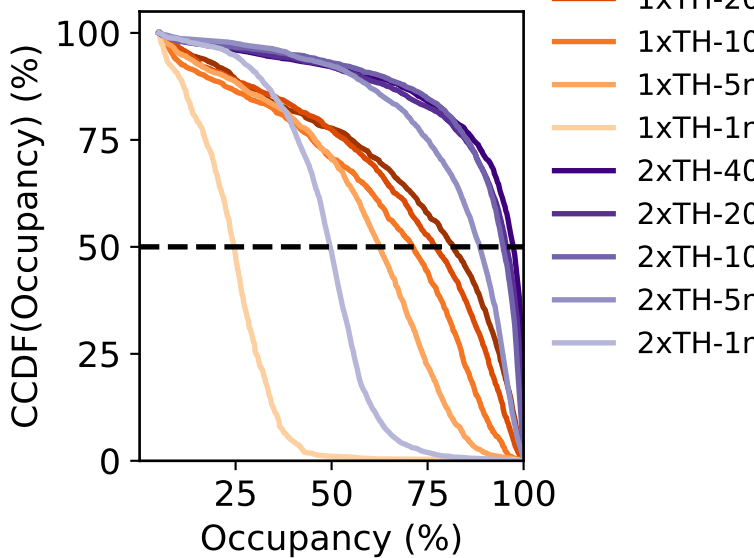

Supplement: Supplementary file 7 — Source Data [file 41467_2021_24223_MOESM7_ESM.zip › z.source-data/si/si_fig23_JS/plots/si_fig23c_occ.pdf]
