# Supplementary figures and images for: Tracking single particles for hours via continuous DNA-mediated fluorophore exchange (part 1 of 2)
Source: Nat Commun. 2021 Jul 21;12:4432. doi: 10.1038/s41467-021-24223-4 (PMC8295357; doi:10.1038/s41467-021-24223-4)

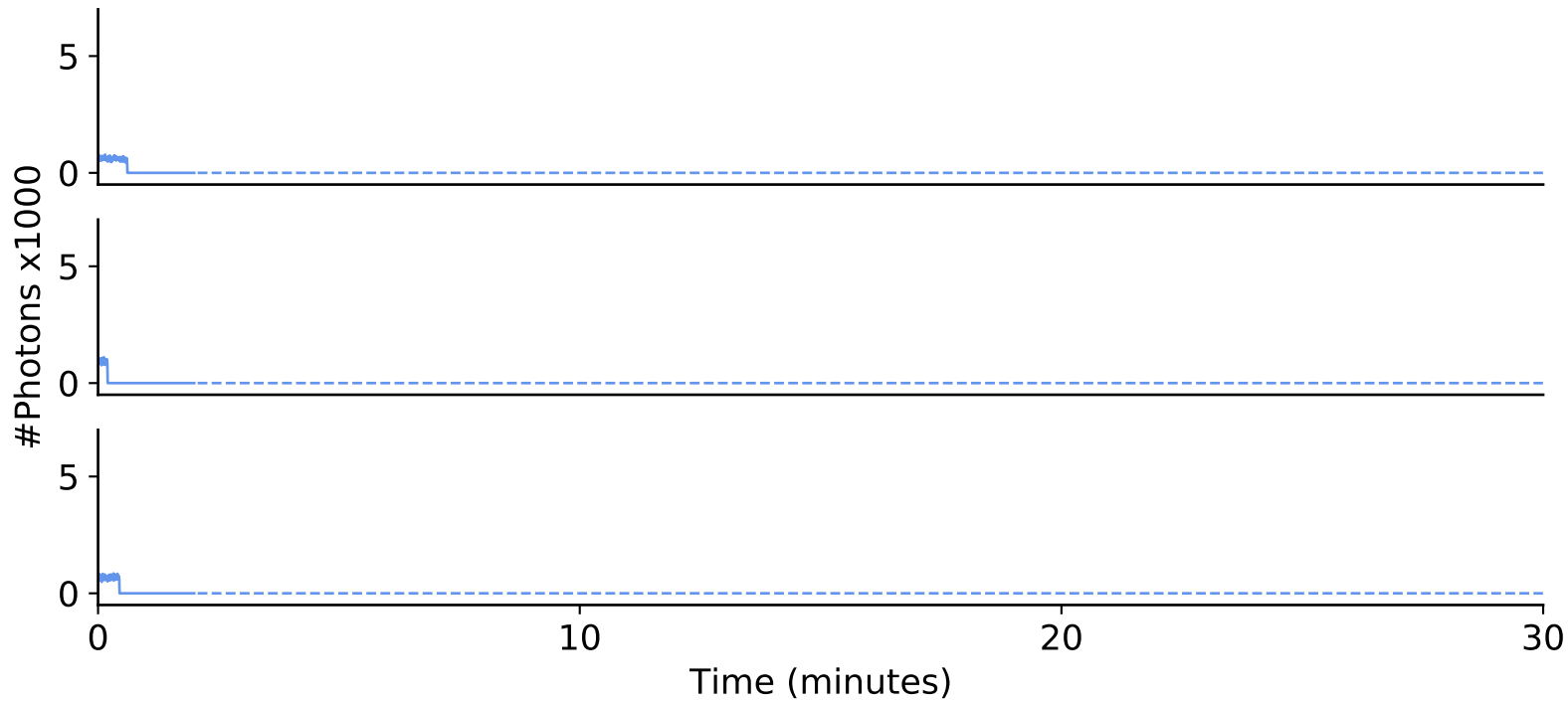

Supplement: Supplementary file 7 — Source Data [file 41467_2021_24223_MOESM7_ESM.zip › z.source-data/main/fig01_JS/c/plots/fig01c_sd_trace.pdf]

#Photons x1000

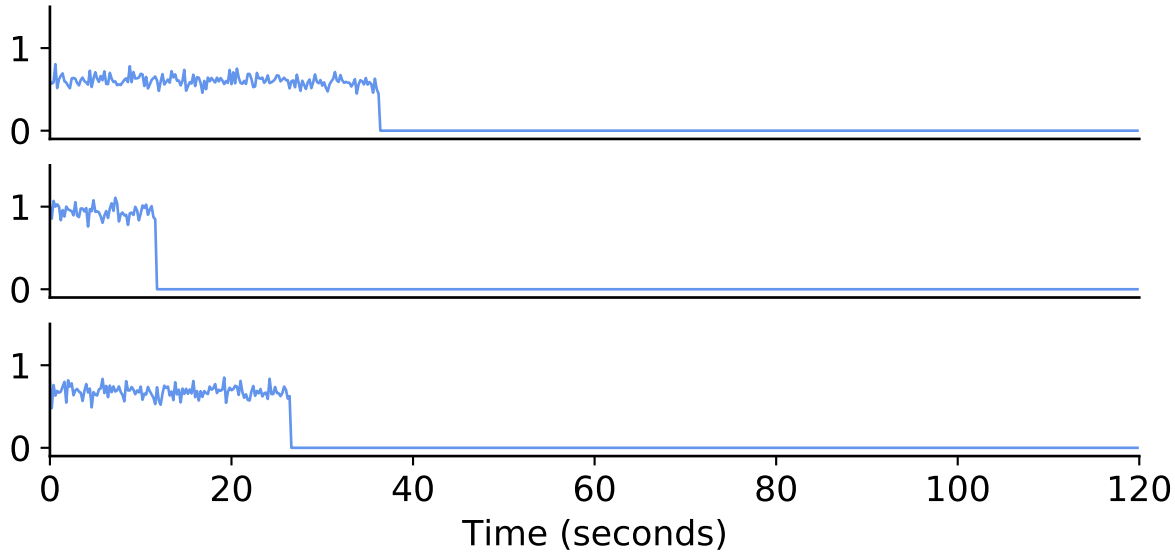

Supplement: Supplementary file 7 — Source Data [file 41467_2021_24223_MOESM7_ESM.zip › z.source-data/main/fig01_JS/c/plots/fig01c_sd_trace_zoom.pdf]

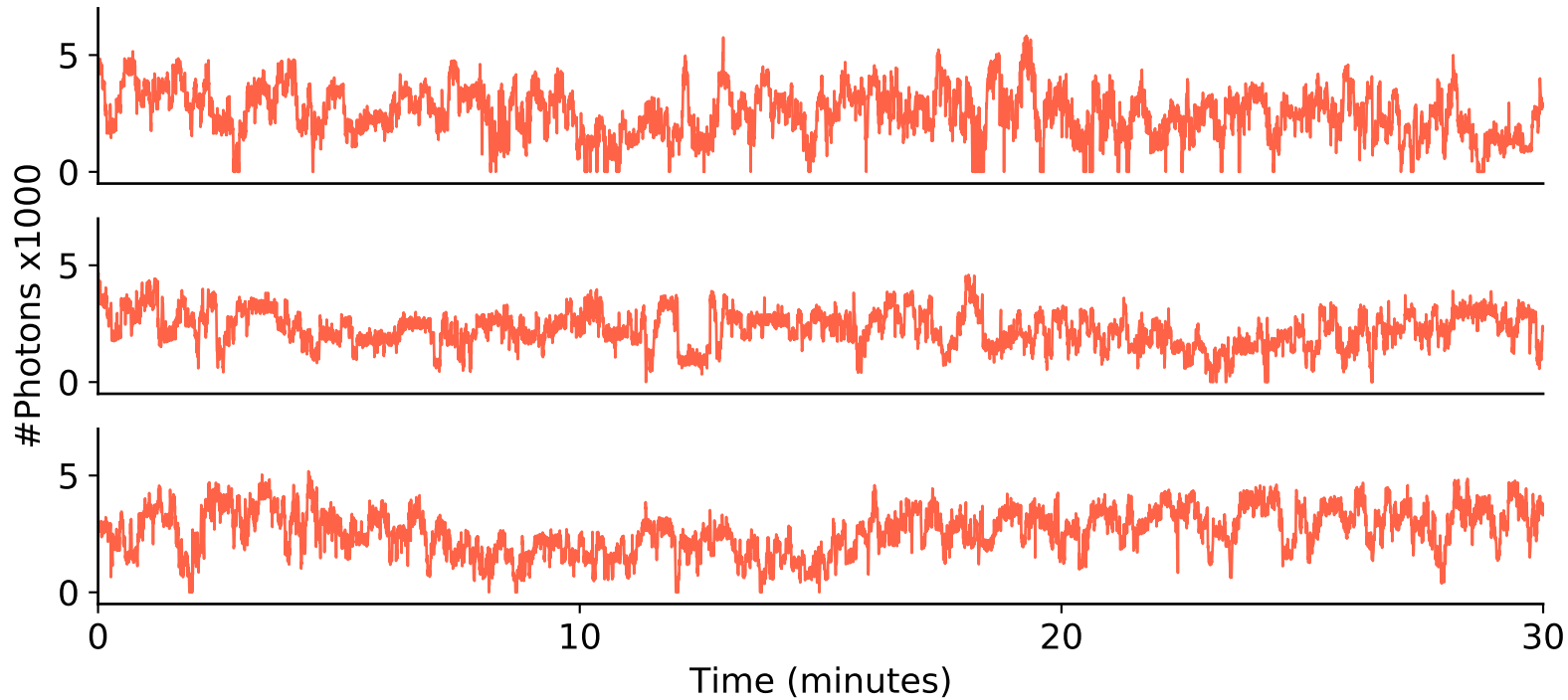

Supplement: Supplementary file 7 — Source Data [file 41467_2021_24223_MOESM7_ESM.zip › z.source-data/main/fig01_JS/d/plots/fig01d_th_trace.pdf]

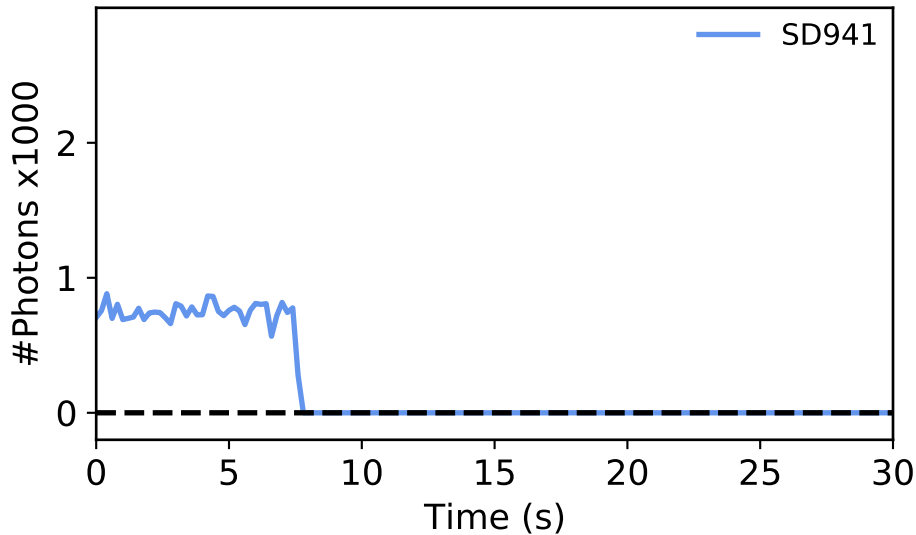

Supplement: Supplementary file 7 — Source Data [file 41467_2021_24223_MOESM7_ESM.zip › z.source-data/main/fig01_JS/f+h/plots/fig01f_trace_zoom.pdf]

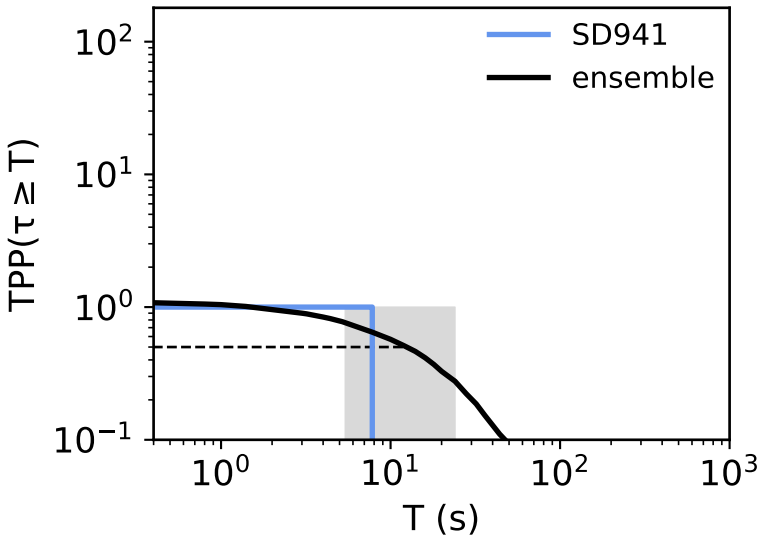

Supplement: Supplementary file 7 — Source Data [file 41467_2021_24223_MOESM7_ESM.zip › z.source-data/main/fig01_JS/f+h/plots/fig01h_sd_NgT.pdf]

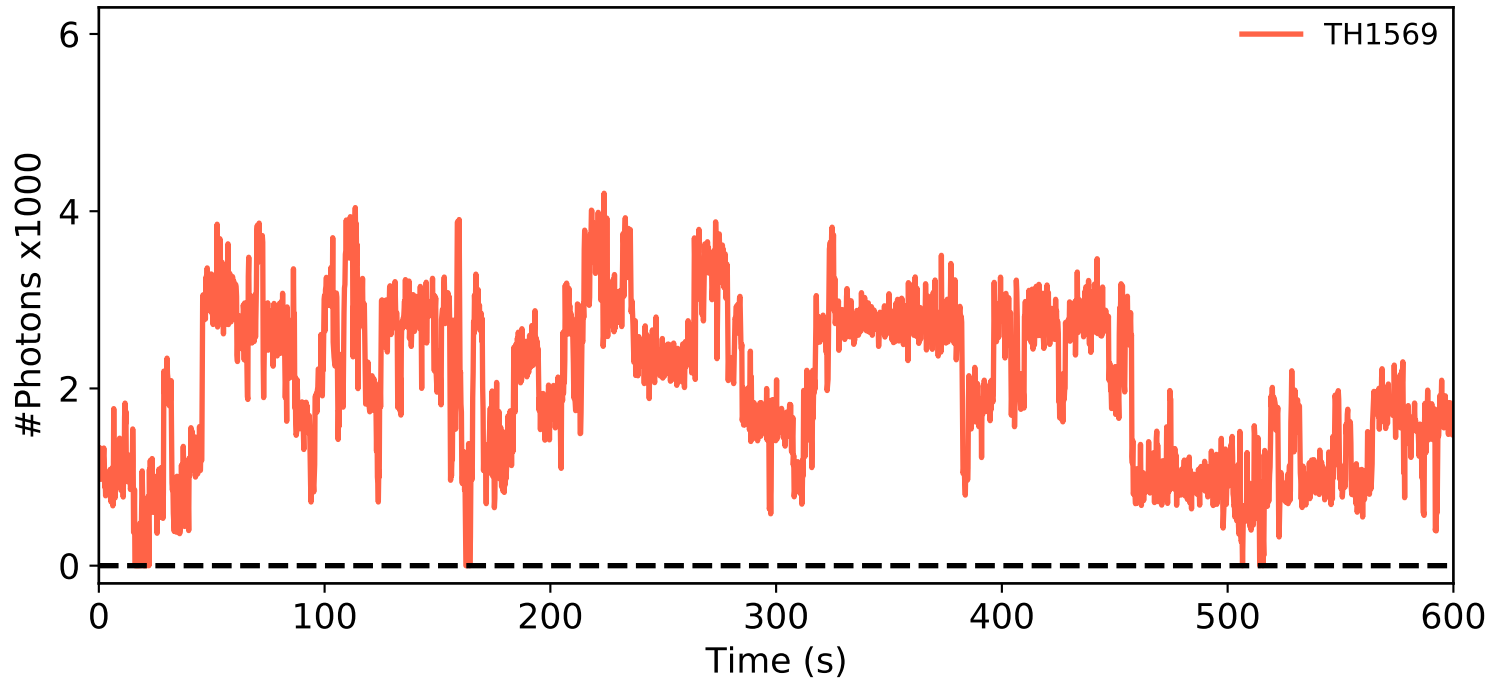

Supplement: Supplementary file 7 — Source Data [file 41467_2021_24223_MOESM7_ESM.zip › z.source-data/main/fig01_JS/g+i/plots/fig01g_th_trace.pdf]

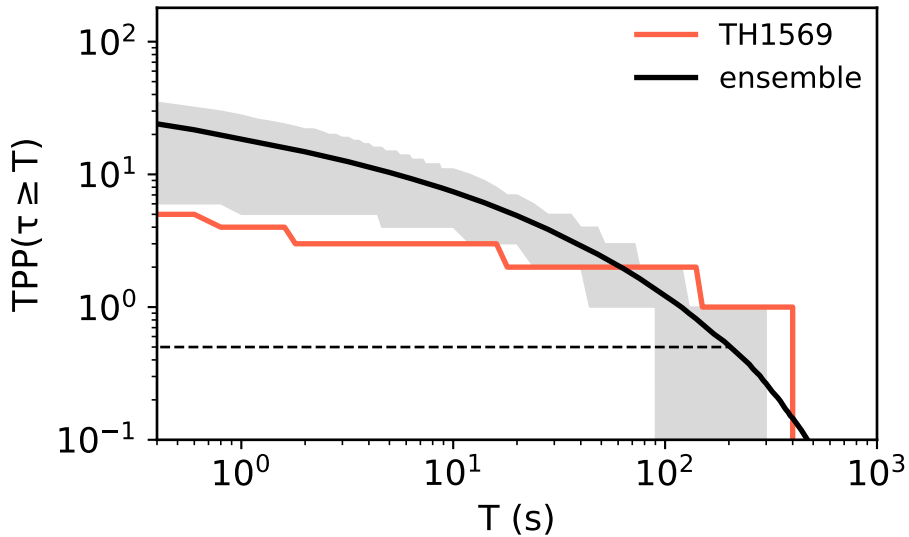

Supplement: Supplementary file 7 — Source Data [file 41467_2021_24223_MOESM7_ESM.zip › z.source-data/main/fig01_JS/g+i/plots/fig01i_th_NgT.pdf]

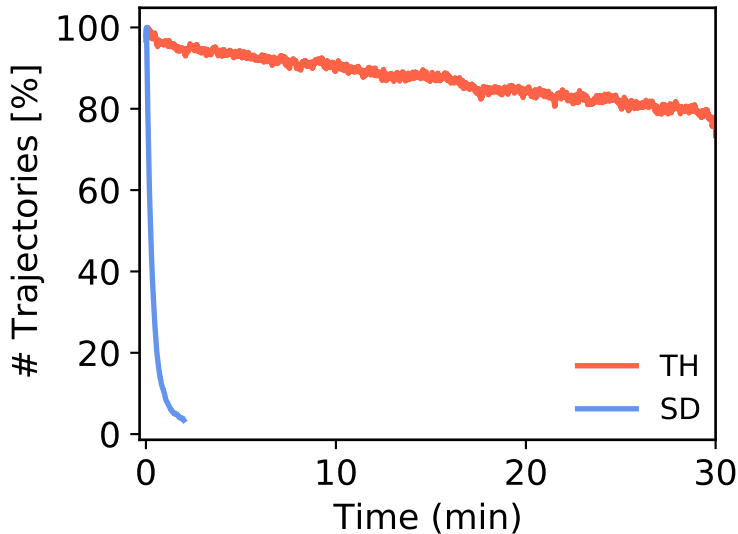

Supplement: Supplementary file 7 — Source Data [file 41467_2021_24223_MOESM7_ESM.zip › z.source-data/main/fig01_JS/j/plots/fig01j_tracks.pdf]

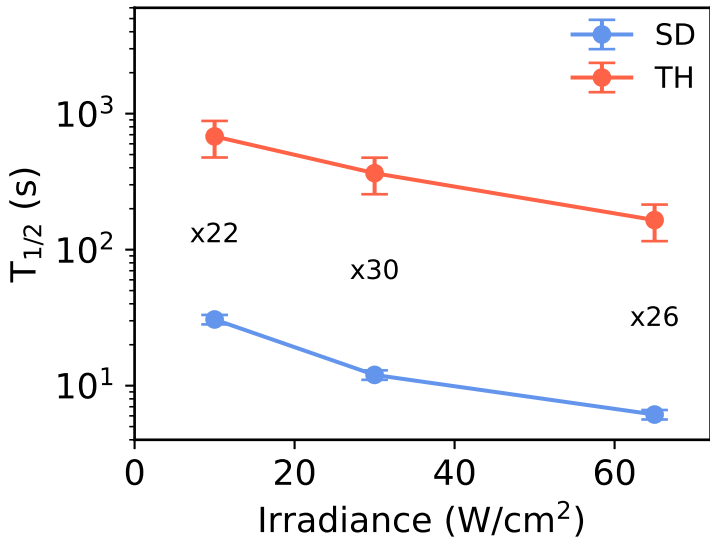

Supplement: Supplementary file 7 — Source Data [file 41467_2021_24223_MOESM7_ESM.zip › z.source-data/main/fig01_JS/k/plots/fig01k_Thalf_TH-vs-SD.pdf]

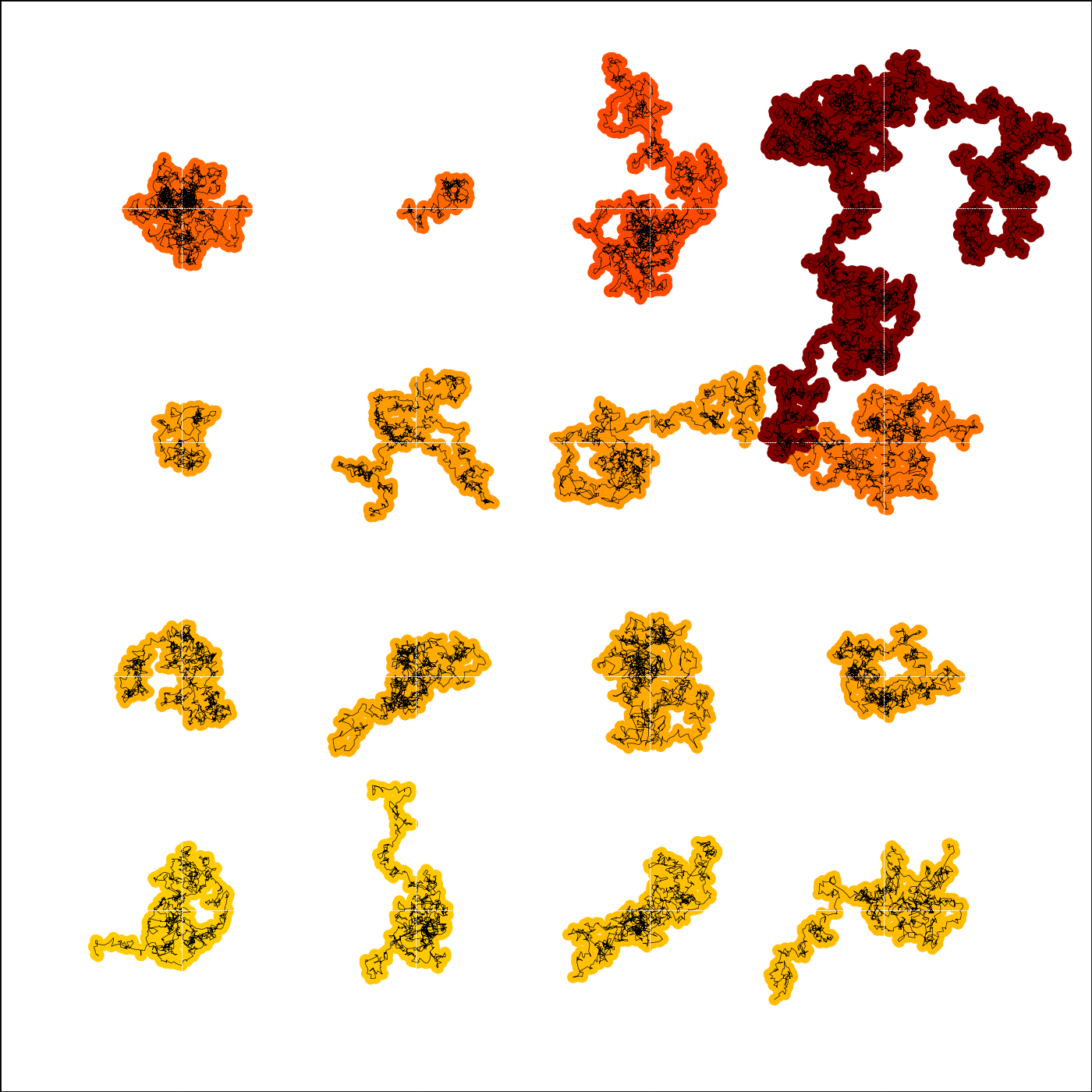

Supplement: Supplementary file 7 — Source Data [file 41467_2021_24223_MOESM7_ESM.zip › z.source-data/main/fig02_FS/a/plots/fig02a_lower_16-trajectories.png]

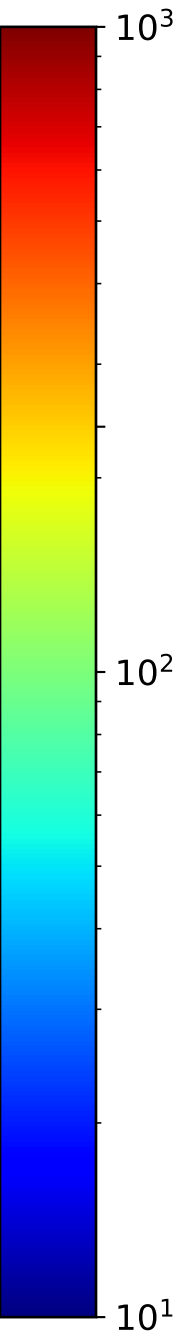

Supplement: Supplementary file 7 — Source Data [file 41467_2021_24223_MOESM7_ESM.zip › z.source-data/main/fig02_FS/a/plots/fig02a_lower_16-trajectories_colorbar.pdf]

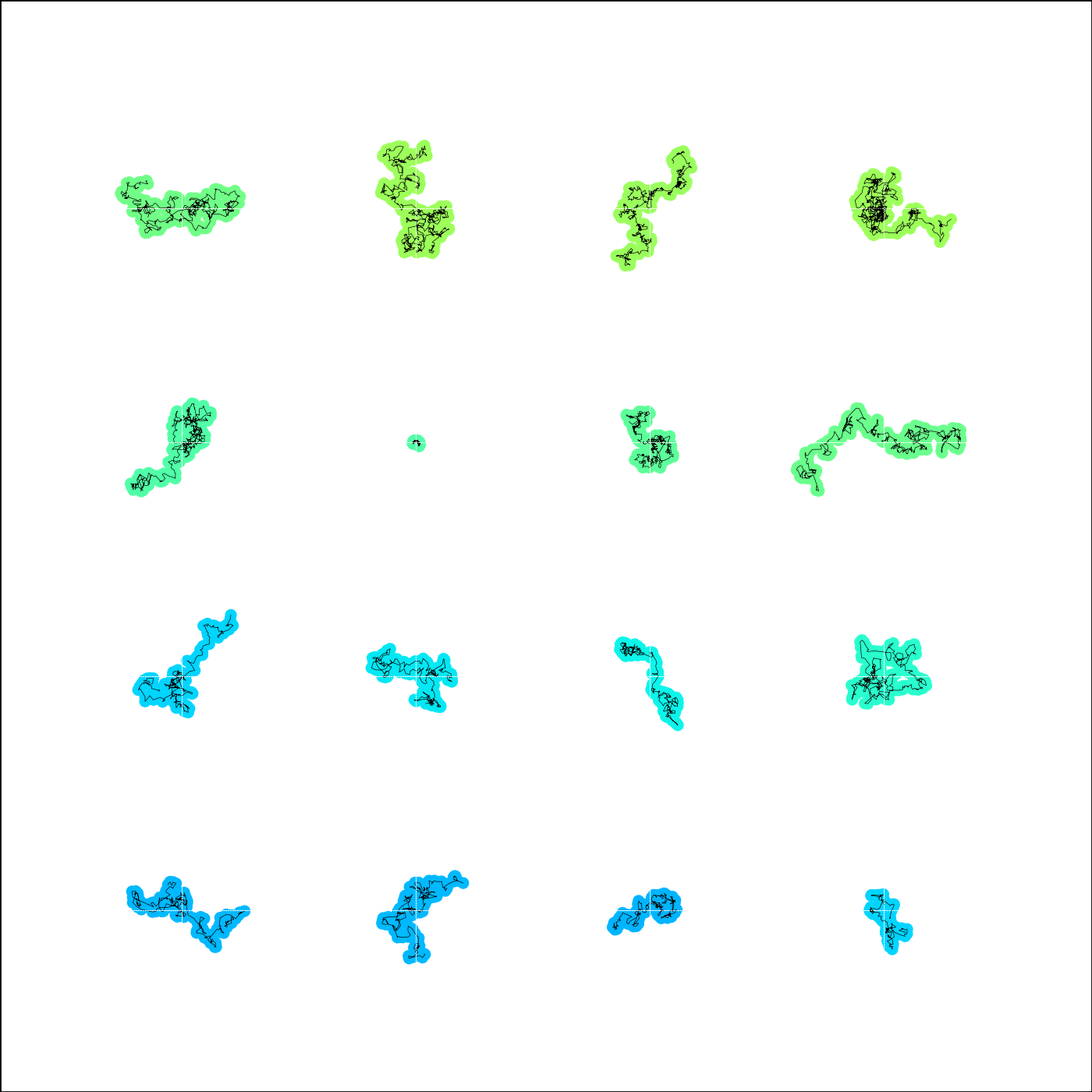

Supplement: Supplementary file 7 — Source Data [file 41467_2021_24223_MOESM7_ESM.zip › z.source-data/main/fig02_FS/a/plots/fig02a_upper_16-trajectories.png]

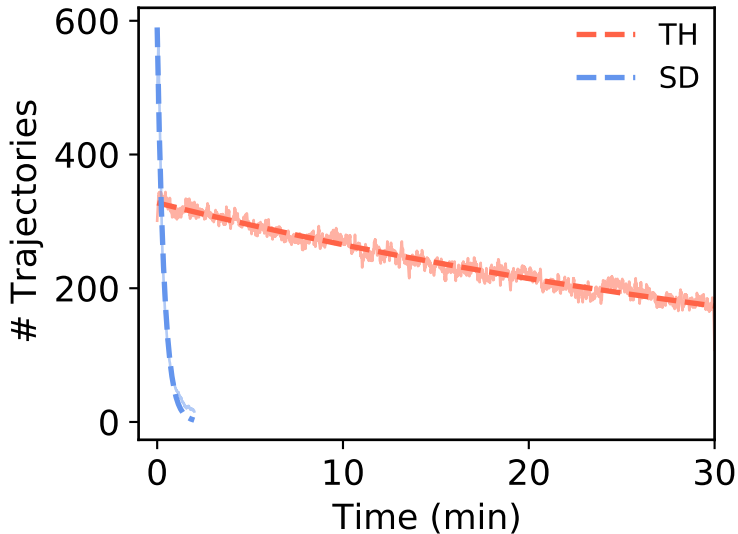

Supplement: Supplementary file 7 — Source Data [file 41467_2021_24223_MOESM7_ESM.zip › z.source-data/main/fig02_FS/b,c,d/plots/fig02b.pdf]

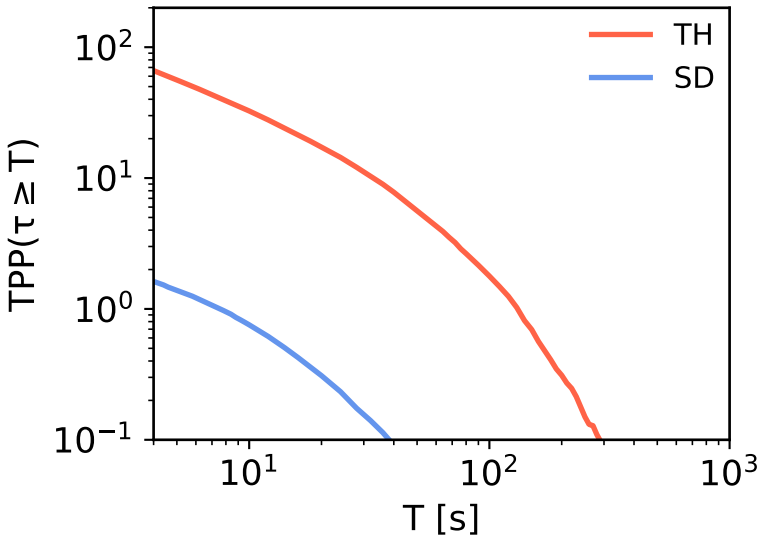

Supplement: Supplementary file 7 — Source Data [file 41467_2021_24223_MOESM7_ESM.zip › z.source-data/main/fig02_FS/b,c,d/plots/fig02c.pdf]

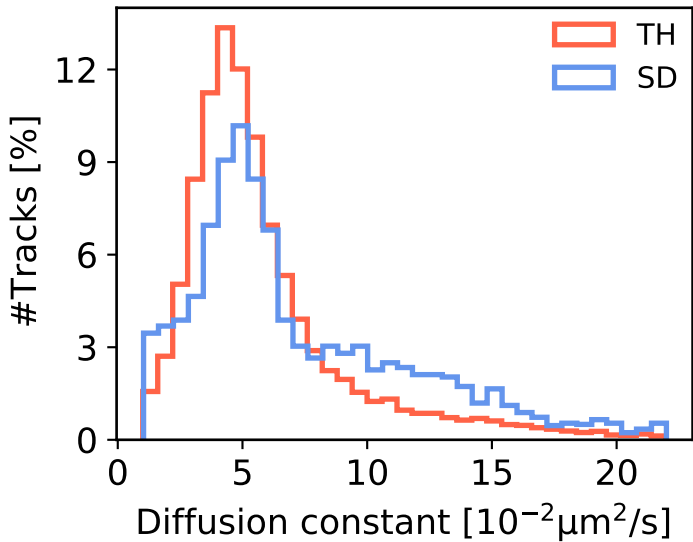

Supplement: Supplementary file 7 — Source Data [file 41467_2021_24223_MOESM7_ESM.zip › z.source-data/main/fig02_FS/b,c,d/plots/fig02d.pdf]

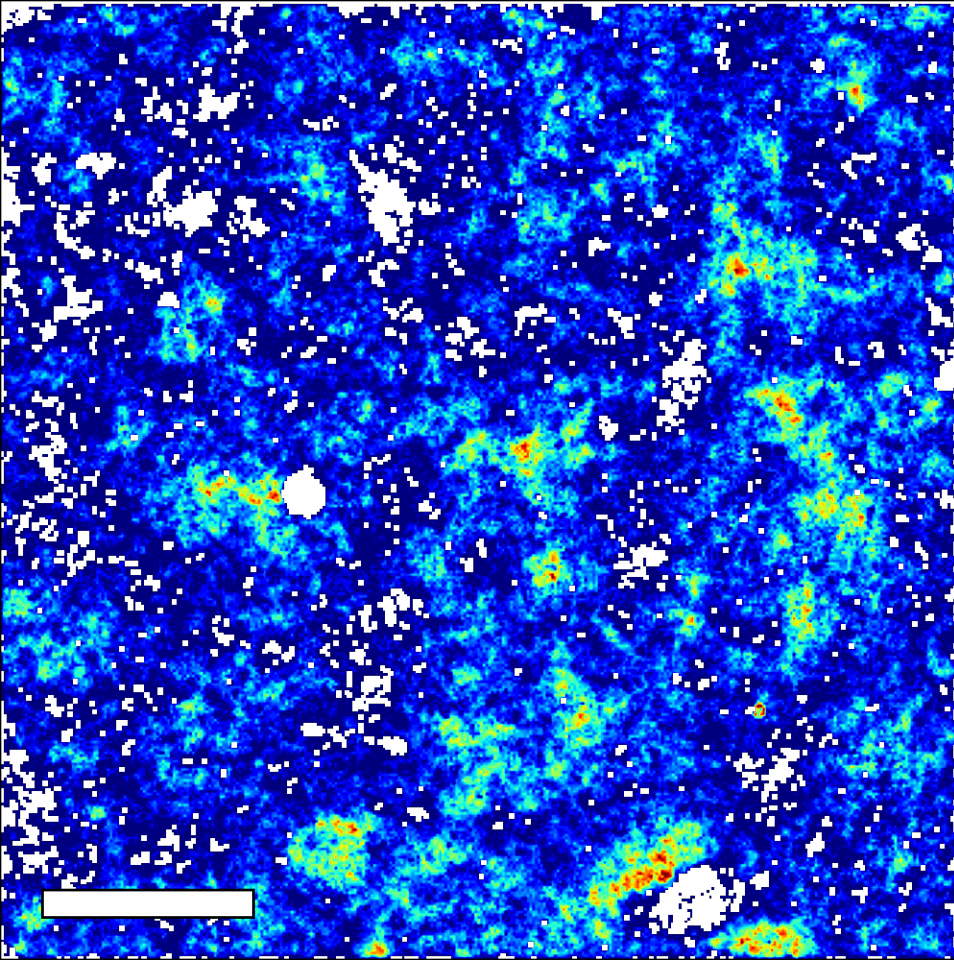

Supplement: Supplementary file 7 — Source Data [file 41467_2021_24223_MOESM7_ESM.zip › z.source-data/main/fig02_FS/e/plots/fig02e_lower_track-map.pdf]

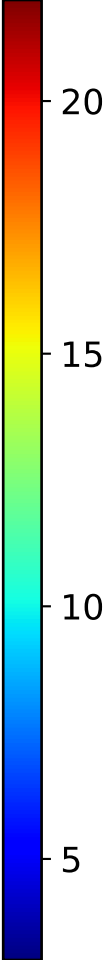

Supplement: Supplementary file 7 — Source Data [file 41467_2021_24223_MOESM7_ESM.zip › z.source-data/main/fig02_FS/e/plots/fig02e_lower_track-map_colorbar.pdf]

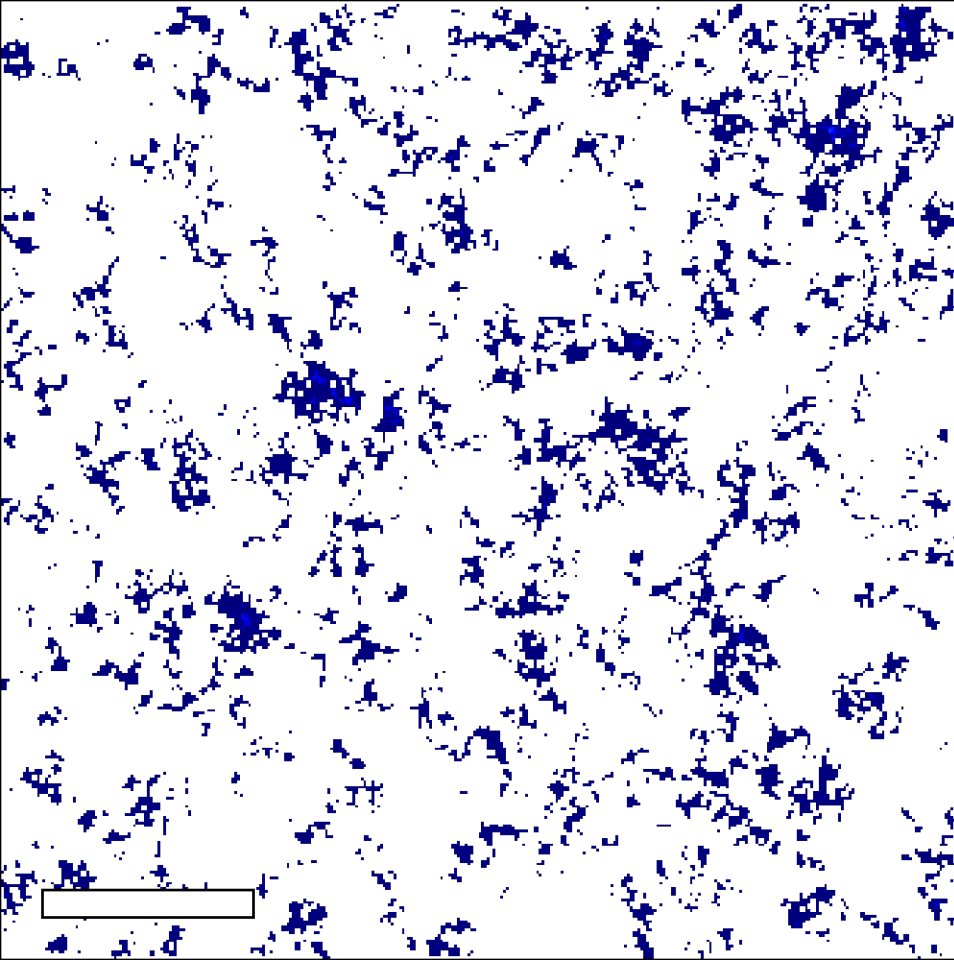

Supplement: Supplementary file 7 — Source Data [file 41467_2021_24223_MOESM7_ESM.zip › z.source-data/main/fig02_FS/e/plots/fig02e_upper_track-map.pdf]

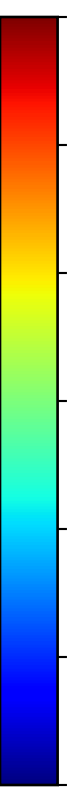

0

2

4

6

8

10

12

Supplement: Supplementary file 7 — Source Data [file 41467_2021_24223_MOESM7_ESM.zip › z.source-data/main/fig03_FS/a/plots/fig03a_bar.pdf]

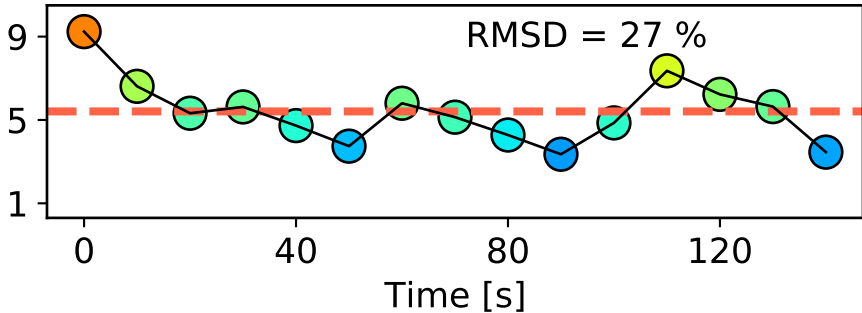

Supplement: Supplementary file 7 — Source Data [file 41467_2021_24223_MOESM7_ESM.zip › z.source-data/main/fig03_FS/a/plots/fig03a_group26359_scatter.pdf]

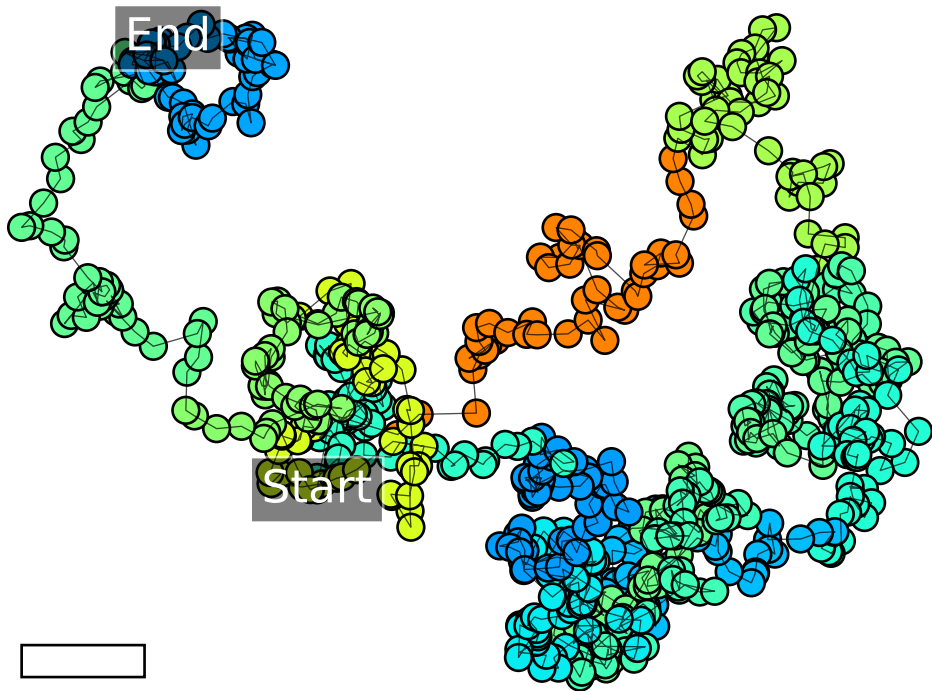

Supplement: Supplementary file 7 — Source Data [file 41467_2021_24223_MOESM7_ESM.zip › z.source-data/main/fig03_FS/a/plots/fig03a_group26359_track.pdf]

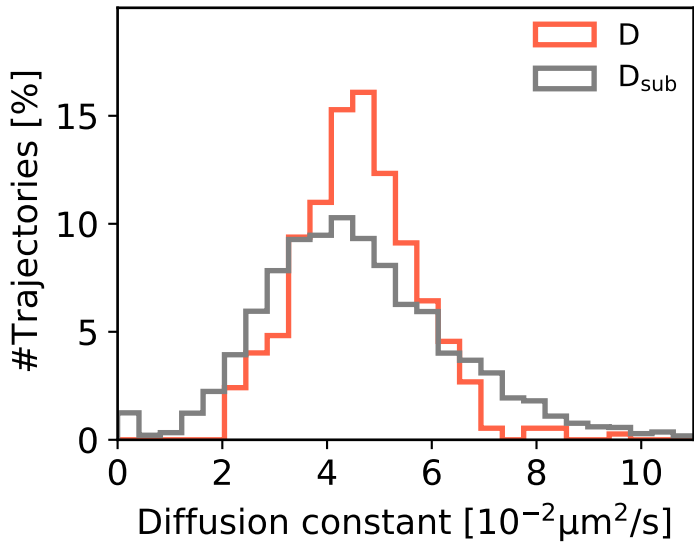

Supplement: Supplementary file 7 — Source Data [file 41467_2021_24223_MOESM7_ESM.zip › z.source-data/main/fig03_FS/b/plots/fig03b_Ds.pdf]

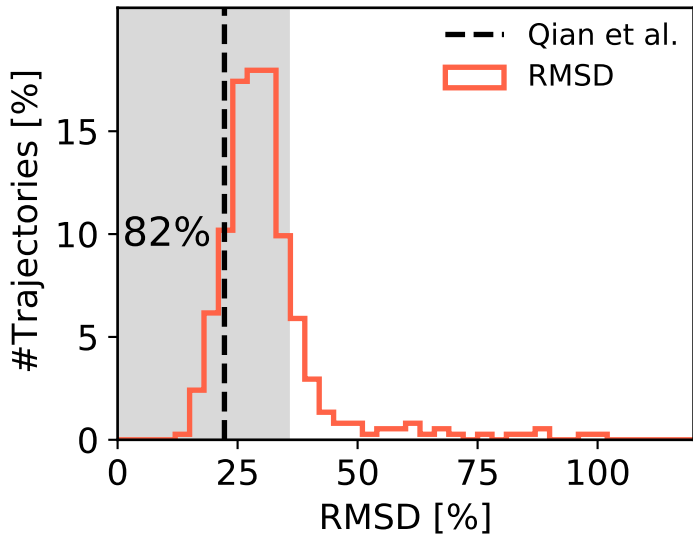

Supplement: Supplementary file 7 — Source Data [file 41467_2021_24223_MOESM7_ESM.zip › z.source-data/main/fig03_FS/c/plots/fig03c_RMSD.pdf]

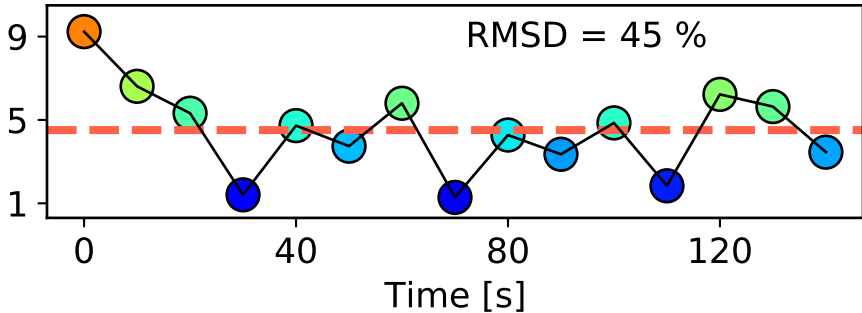

Supplement: Supplementary file 7 — Source Data [file 41467_2021_24223_MOESM7_ESM.zip › z.source-data/main/fig03_FS/d/plots/fig03d_group26359_scatter.pdf]

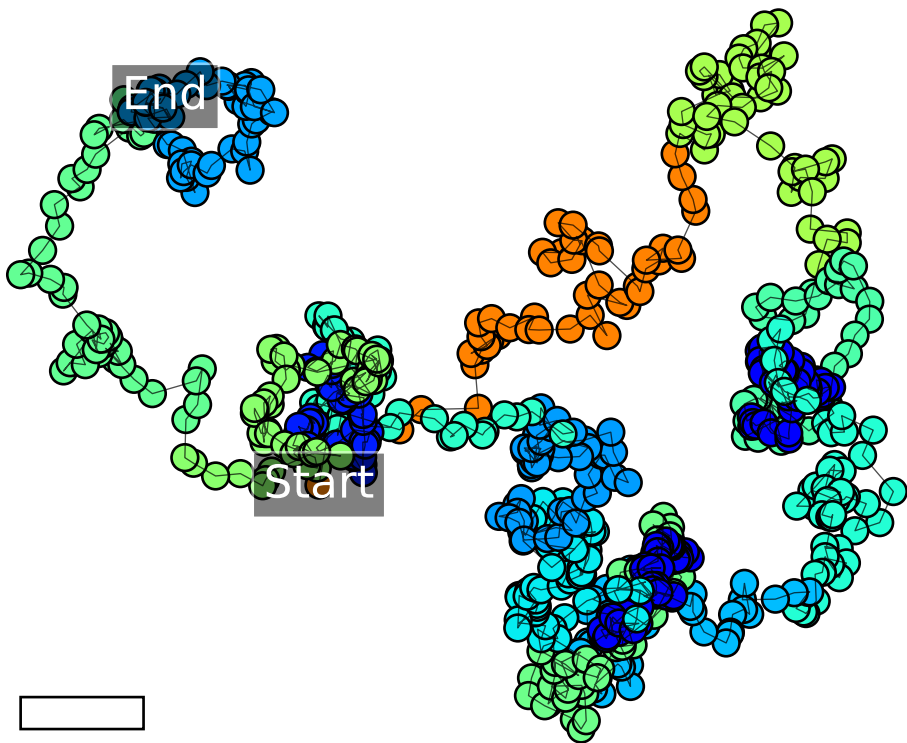

Supplement: Supplementary file 7 — Source Data [file 41467_2021_24223_MOESM7_ESM.zip › z.source-data/main/fig03_FS/d/plots/fig03d_group26359_track.pdf]

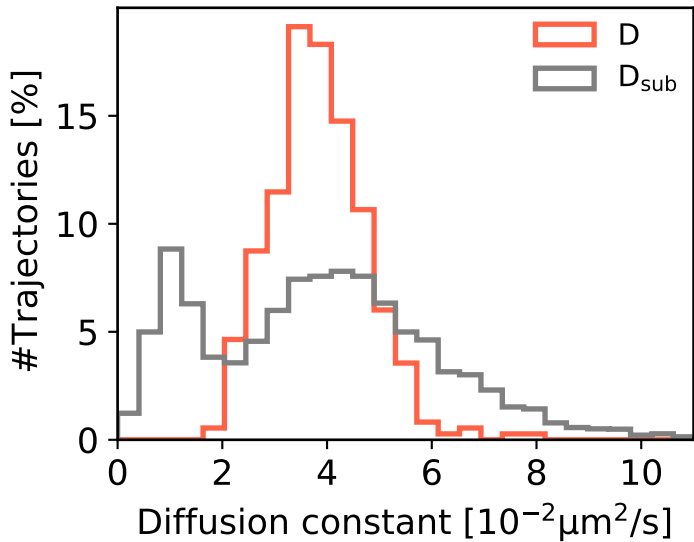

Supplement: Supplementary file 7 — Source Data [file 41467_2021_24223_MOESM7_ESM.zip › z.source-data/main/fig03_FS/e/plots/fig03e_Ds.pdf]

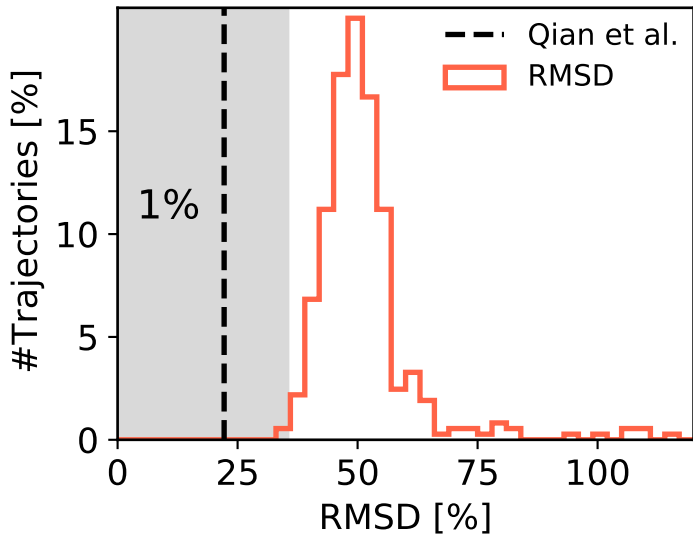

Supplement: Supplementary file 7 — Source Data [file 41467_2021_24223_MOESM7_ESM.zip › z.source-data/main/fig03_FS/f/plots/fig03f_RMSD.pdf]

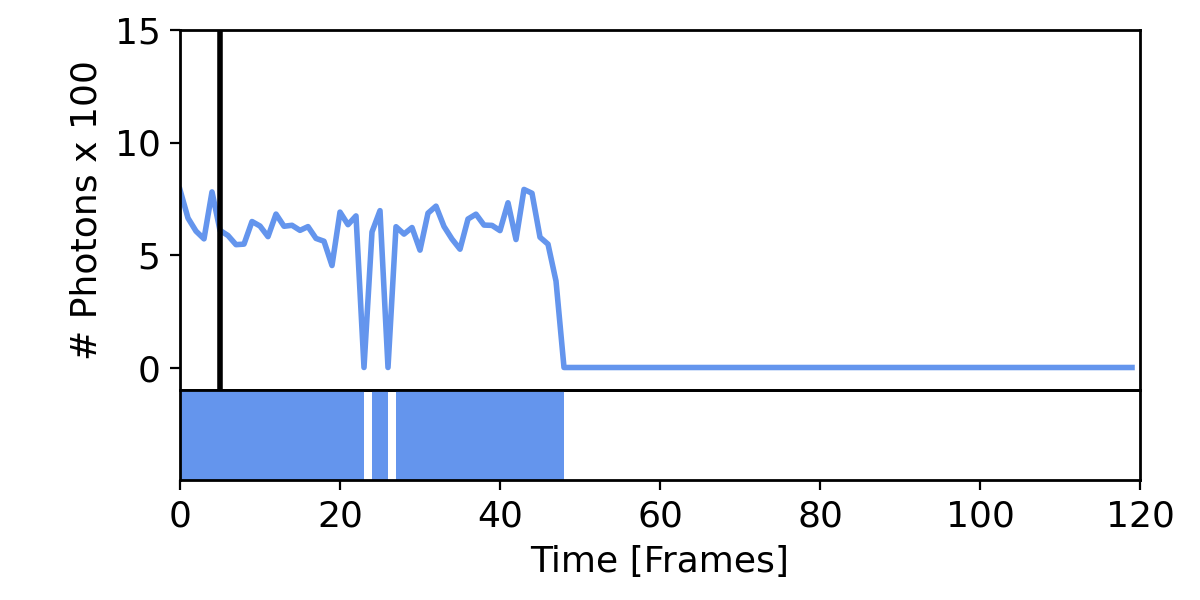

Supplement: Supplementary file 7 — Source Data [file 41467_2021_24223_MOESM7_ESM.zip › z.source-data/si/si_fig04_FS/a-b/plots/fig04a.png]

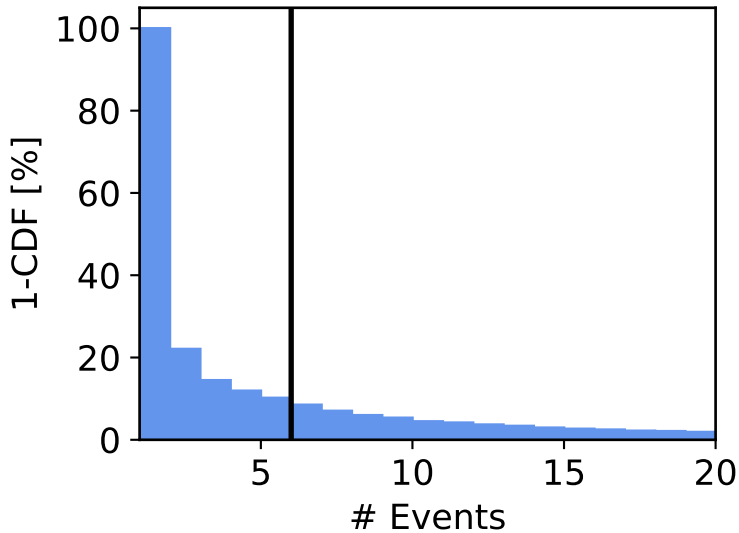

Supplement: Supplementary file 7 — Source Data [file 41467_2021_24223_MOESM7_ESM.zip › z.source-data/si/si_fig04_FS/a-b/plots/fig04b.pdf]

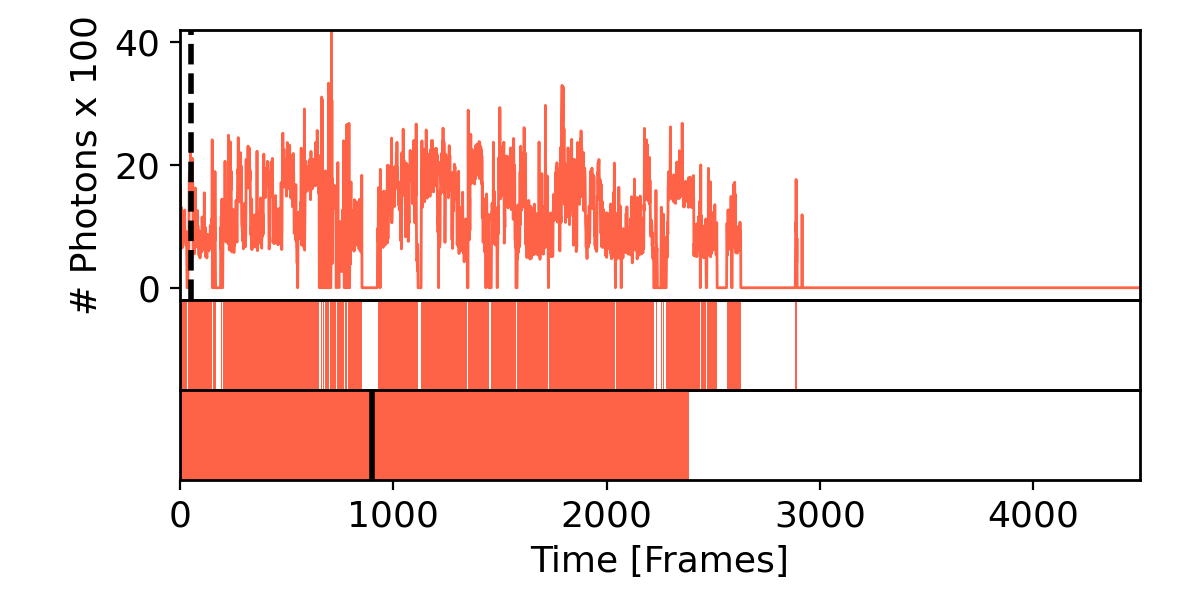

Supplement: Supplementary file 7 — Source Data [file 41467_2021_24223_MOESM7_ESM.zip › z.source-data/si/si_fig04_FS/c-d/plots/fig04c.png]

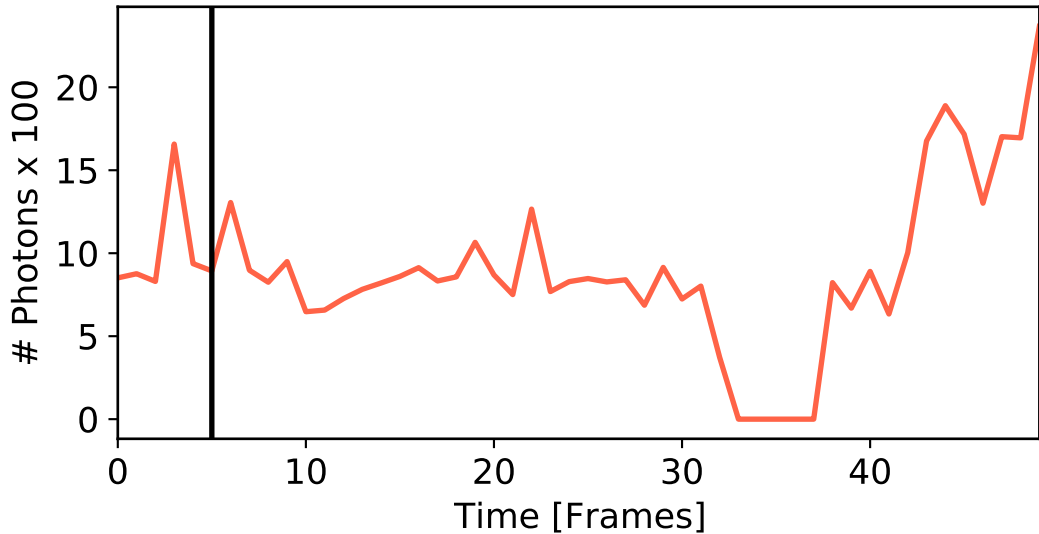

Supplement: Supplementary file 7 — Source Data [file 41467_2021_24223_MOESM7_ESM.zip › z.source-data/si/si_fig04_FS/c-d/plots/fig04d.pdf]

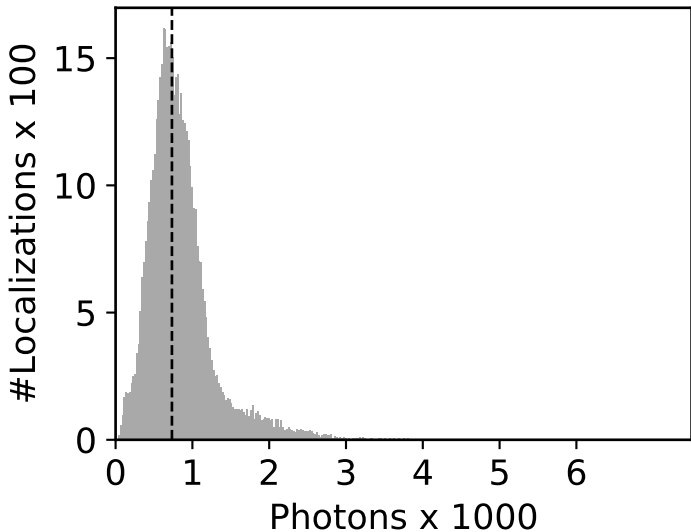

Supplement: Supplementary file 7 — Source Data [file 41467_2021_24223_MOESM7_ESM.zip › z.source-data/si/si_fig05_FS/a-c/plots/fig05a_lower.pdf]

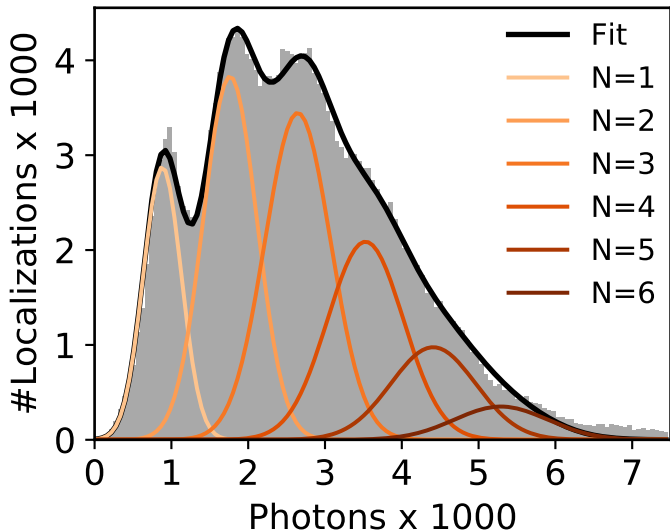

Supplement: Supplementary file 7 — Source Data [file 41467_2021_24223_MOESM7_ESM.zip › z.source-data/si/si_fig05_FS/a-c/plots/fig05a_upper.pdf]

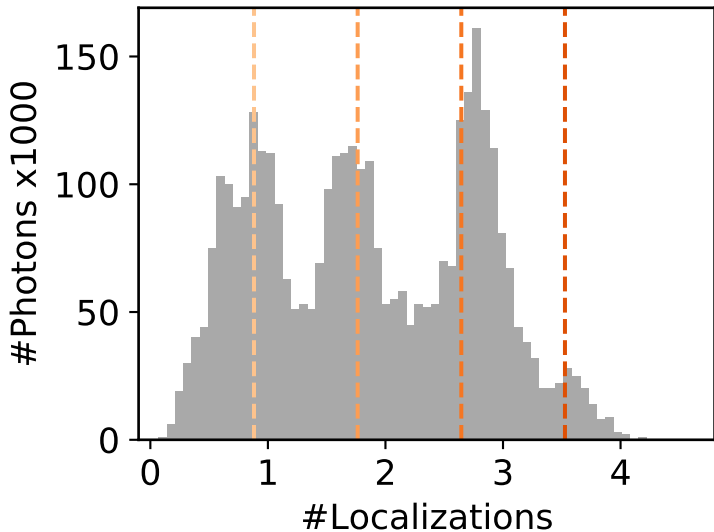

Supplement: Supplementary file 7 — Source Data [file 41467_2021_24223_MOESM7_ESM.zip › z.source-data/si/si_fig05_FS/a-c/plots/fig05b_left.pdf]

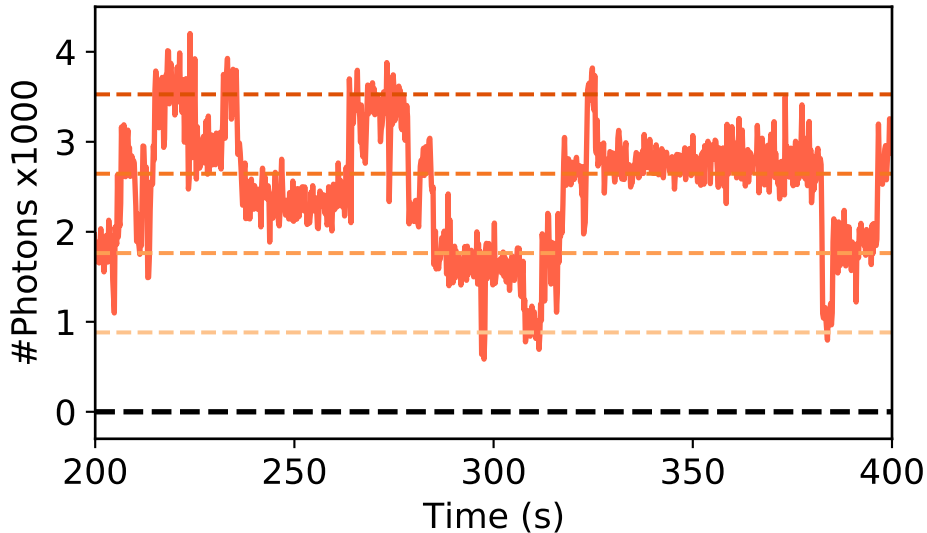

Supplement: Supplementary file 7 — Source Data [file 41467_2021_24223_MOESM7_ESM.zip › z.source-data/si/si_fig05_FS/a-c/plots/fig05b_right.pdf]

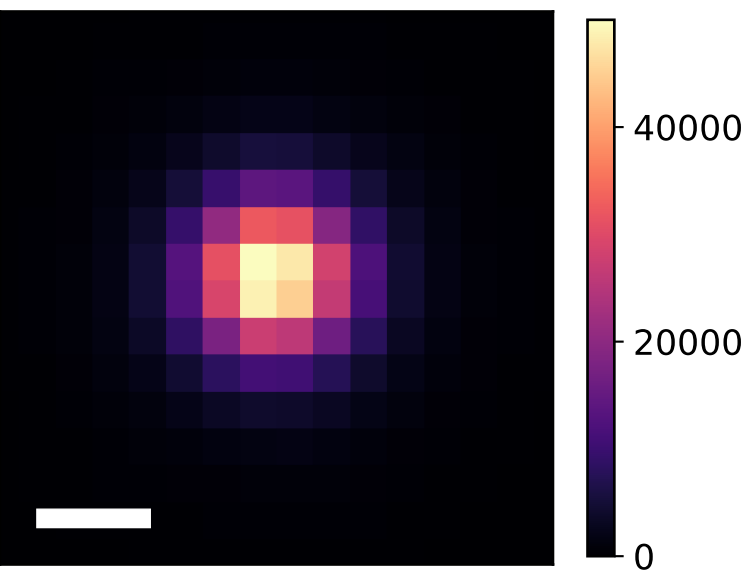

Supplement: Supplementary file 7 — Source Data [file 41467_2021_24223_MOESM7_ESM.zip › z.source-data/si/si_fig05_FS/a-c/plots/fig05c_lower_avg.pdf]

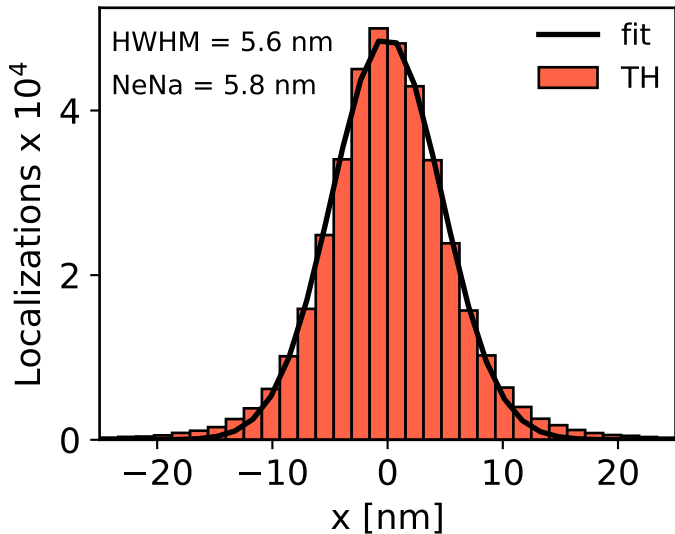

Supplement: Supplementary file 7 — Source Data [file 41467_2021_24223_MOESM7_ESM.zip › z.source-data/si/si_fig05_FS/a-c/plots/fig05c_lower_avg-line.pdf]

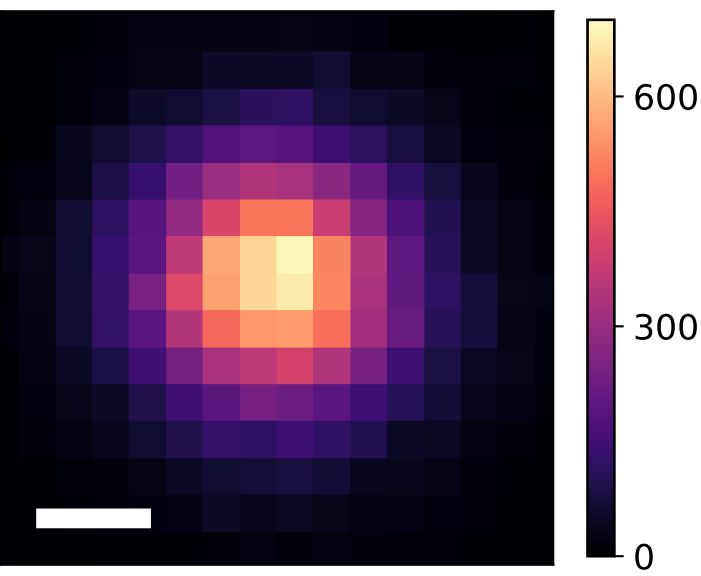

Supplement: Supplementary file 7 — Source Data [file 41467_2021_24223_MOESM7_ESM.zip › z.source-data/si/si_fig05_FS/a-c/plots/fig05c_upper_avg.pdf]

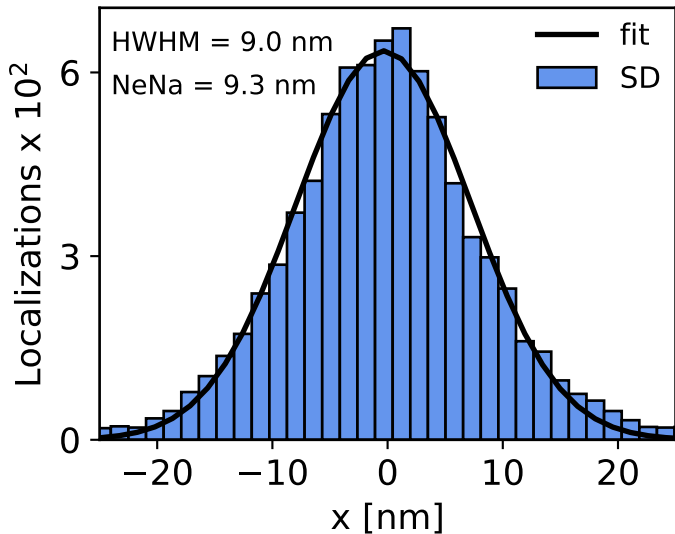

Supplement: Supplementary file 7 — Source Data [file 41467_2021_24223_MOESM7_ESM.zip › z.source-data/si/si_fig05_FS/a-c/plots/fig05c_upper_lineplot.pdf]

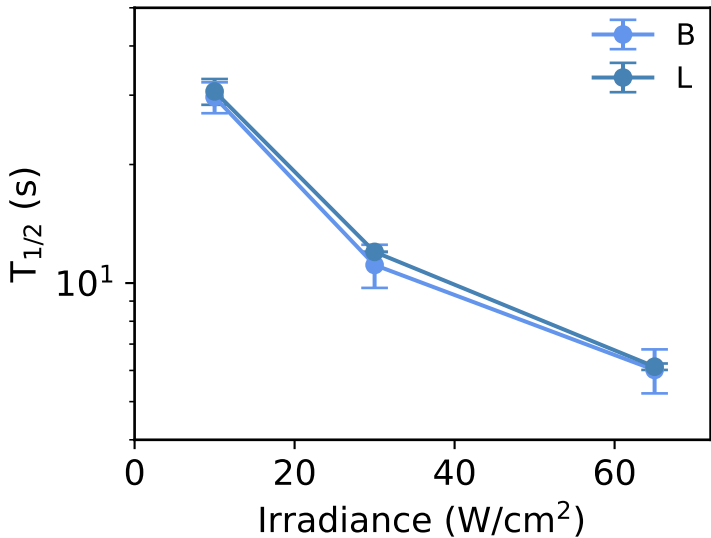

Supplement: Supplementary file 7 — Source Data [file 41467_2021_24223_MOESM7_ESM.zip › z.source-data/si/si_fig06_JS/a/plots/si_fig06a_SD.pdf]

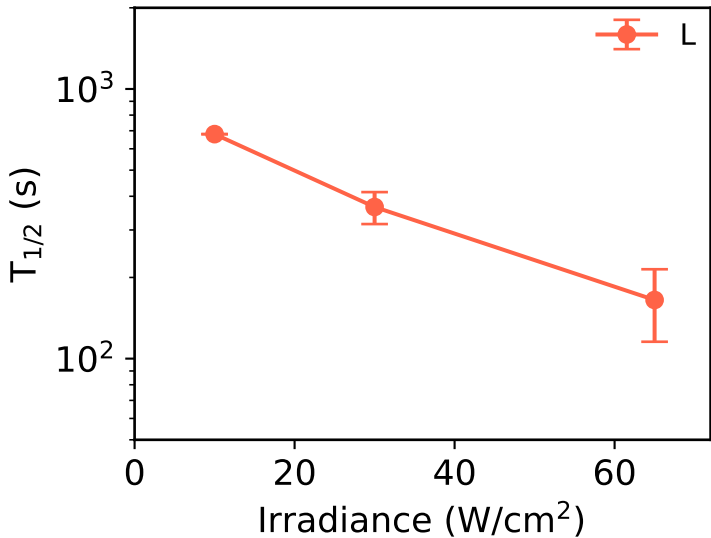

Supplement: Supplementary file 7 — Source Data [file 41467_2021_24223_MOESM7_ESM.zip › z.source-data/si/si_fig06_JS/b/plots/si_fig06b_TH.pdf]

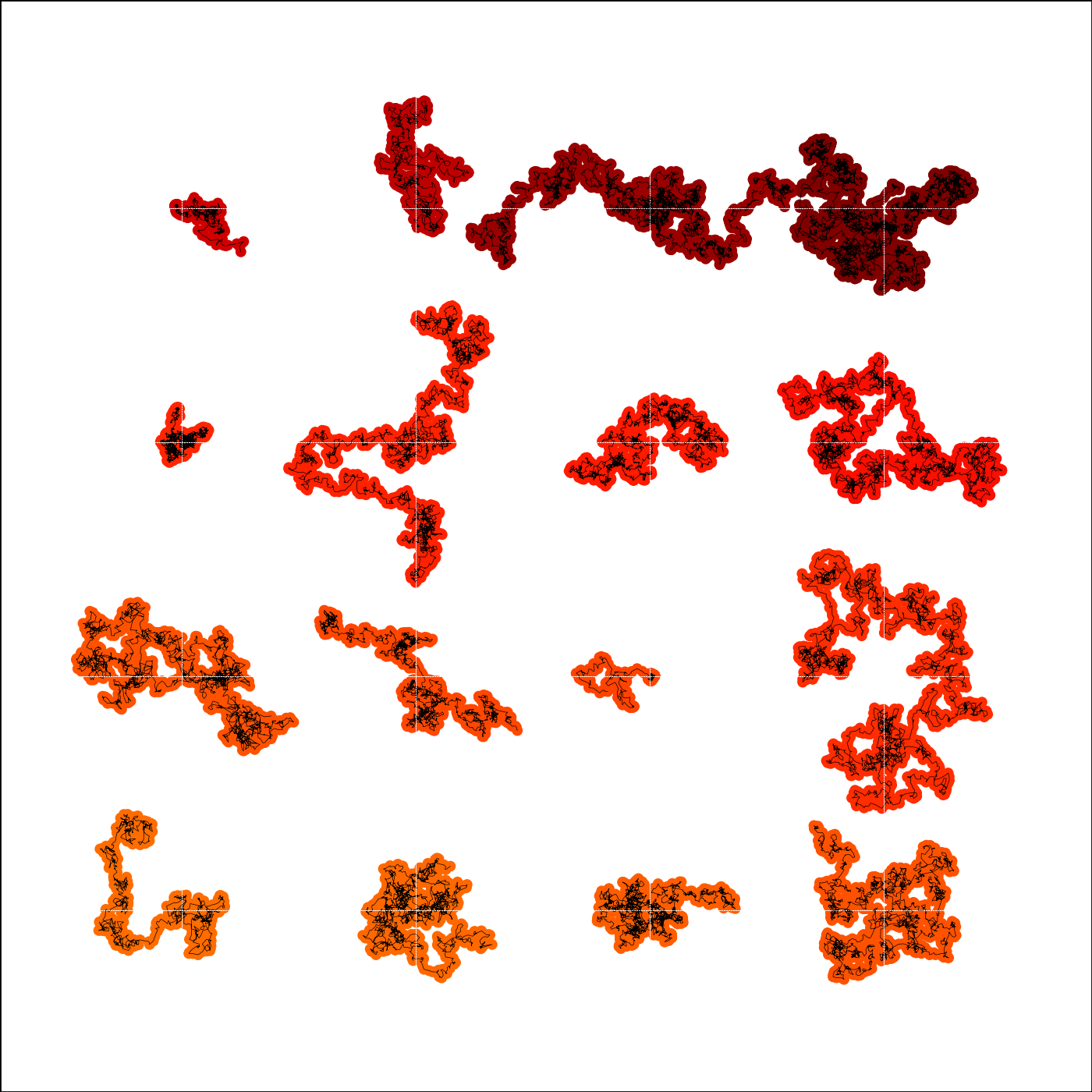

Supplement: Supplementary file 7 — Source Data [file 41467_2021_24223_MOESM7_ESM.zip › z.source-data/si/si_fig08_FS/a/plots/fig08a_lower.png]

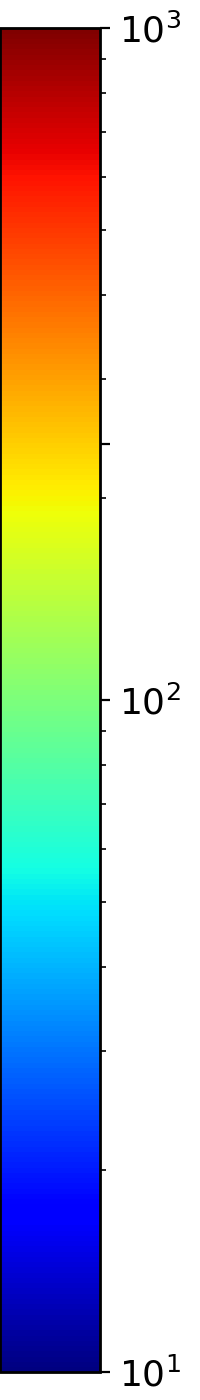

Supplement: Supplementary file 7 — Source Data [file 41467_2021_24223_MOESM7_ESM.zip › z.source-data/si/si_fig08_FS/a/plots/fig08a_lower_colorbar.png]

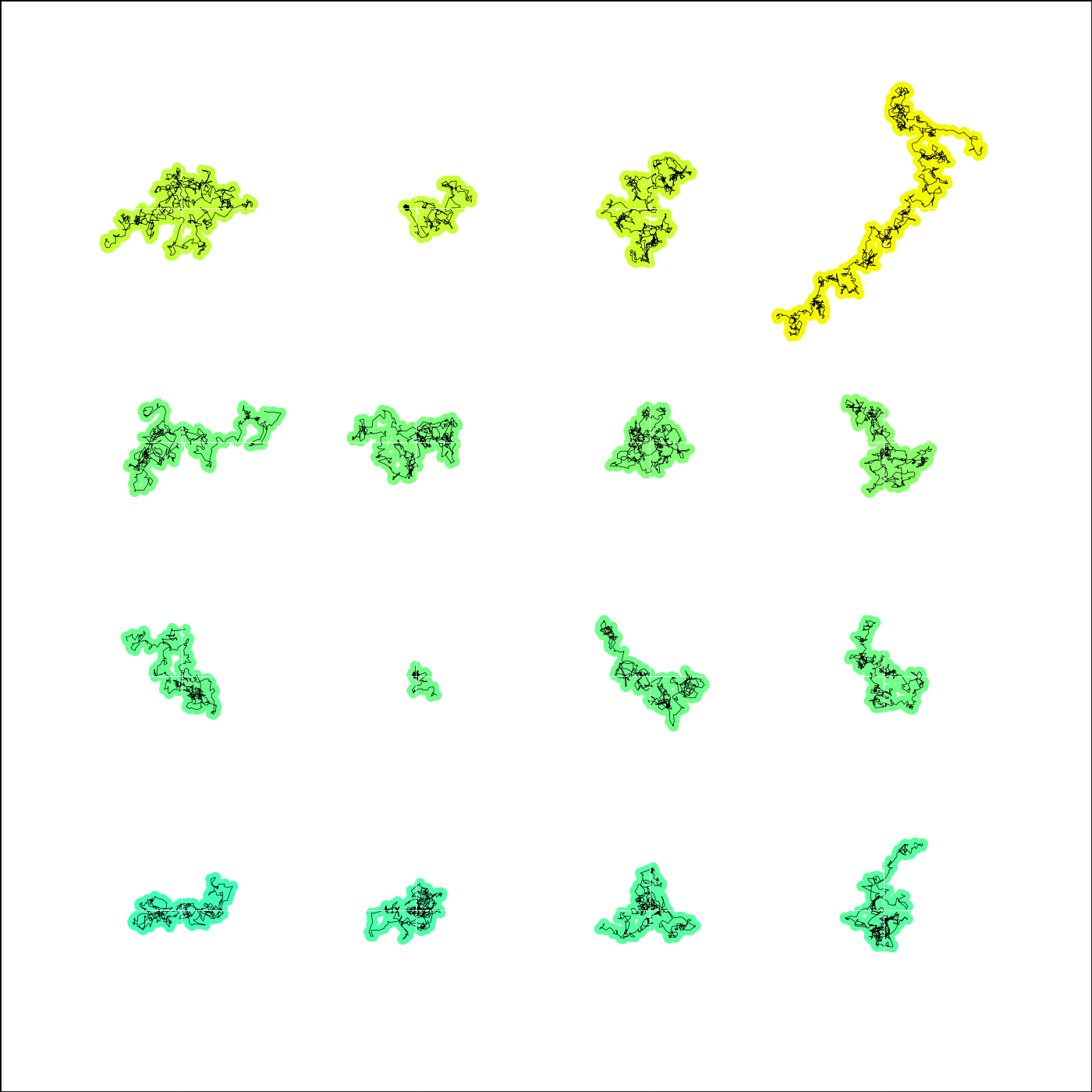

Supplement: Supplementary file 7 — Source Data [file 41467_2021_24223_MOESM7_ESM.zip › z.source-data/si/si_fig08_FS/a/plots/fig08a_upper.png]

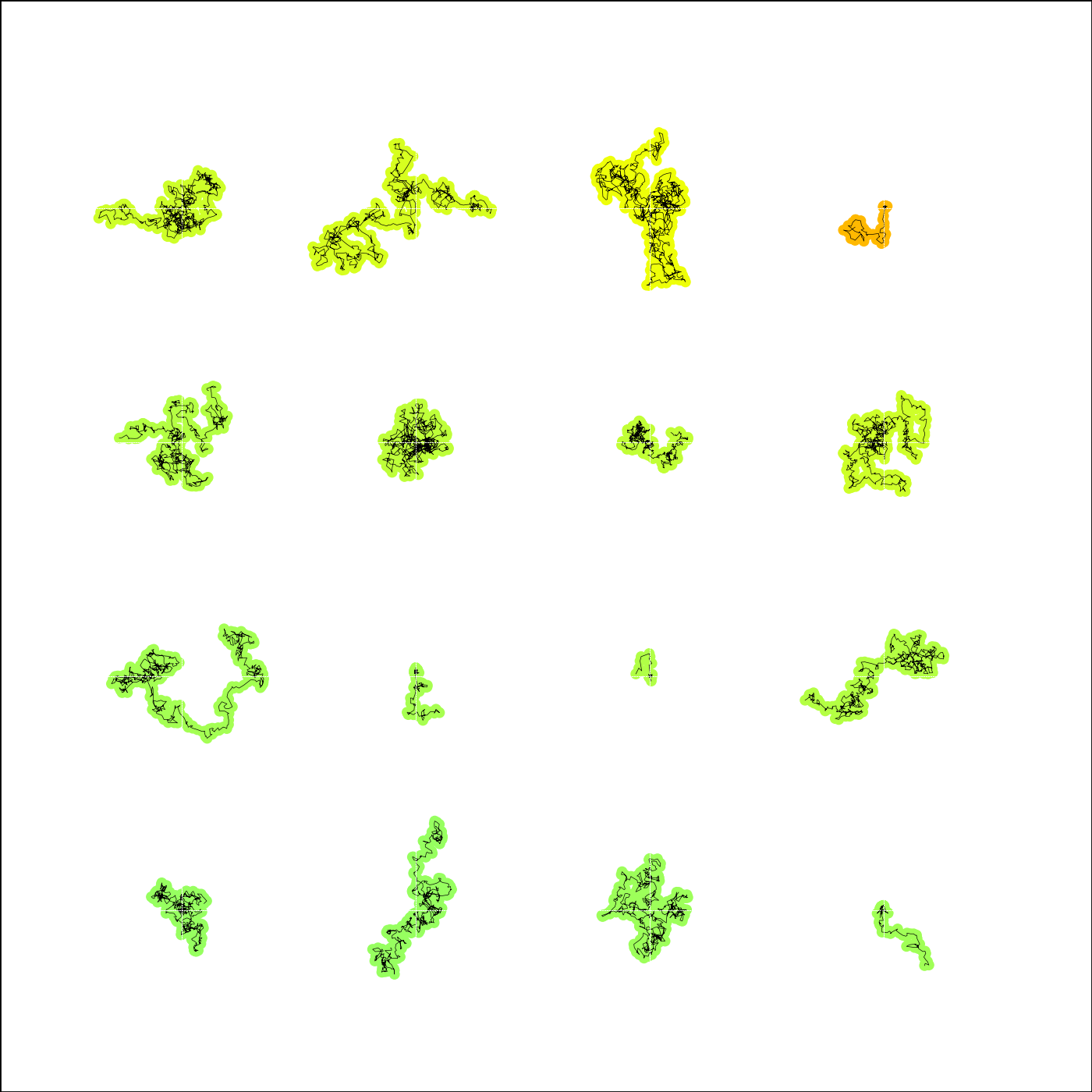

Supplement: Supplementary file 7 — Source Data [file 41467_2021_24223_MOESM7_ESM.zip › z.source-data/si/si_fig08_FS/b/plots/fig08b_lower.png]

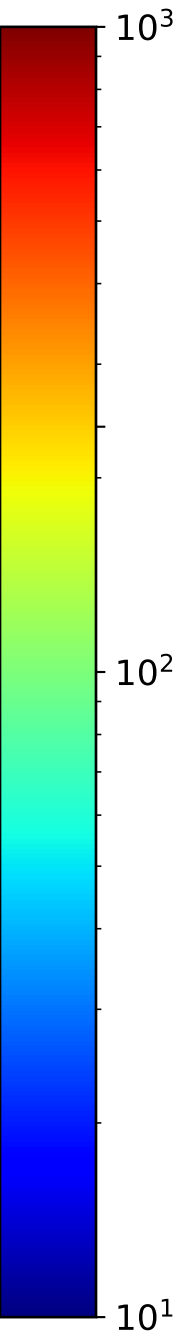

Supplement: Supplementary file 7 — Source Data [file 41467_2021_24223_MOESM7_ESM.zip › z.source-data/si/si_fig08_FS/b/plots/fig08b_lower_colorbar.pdf]

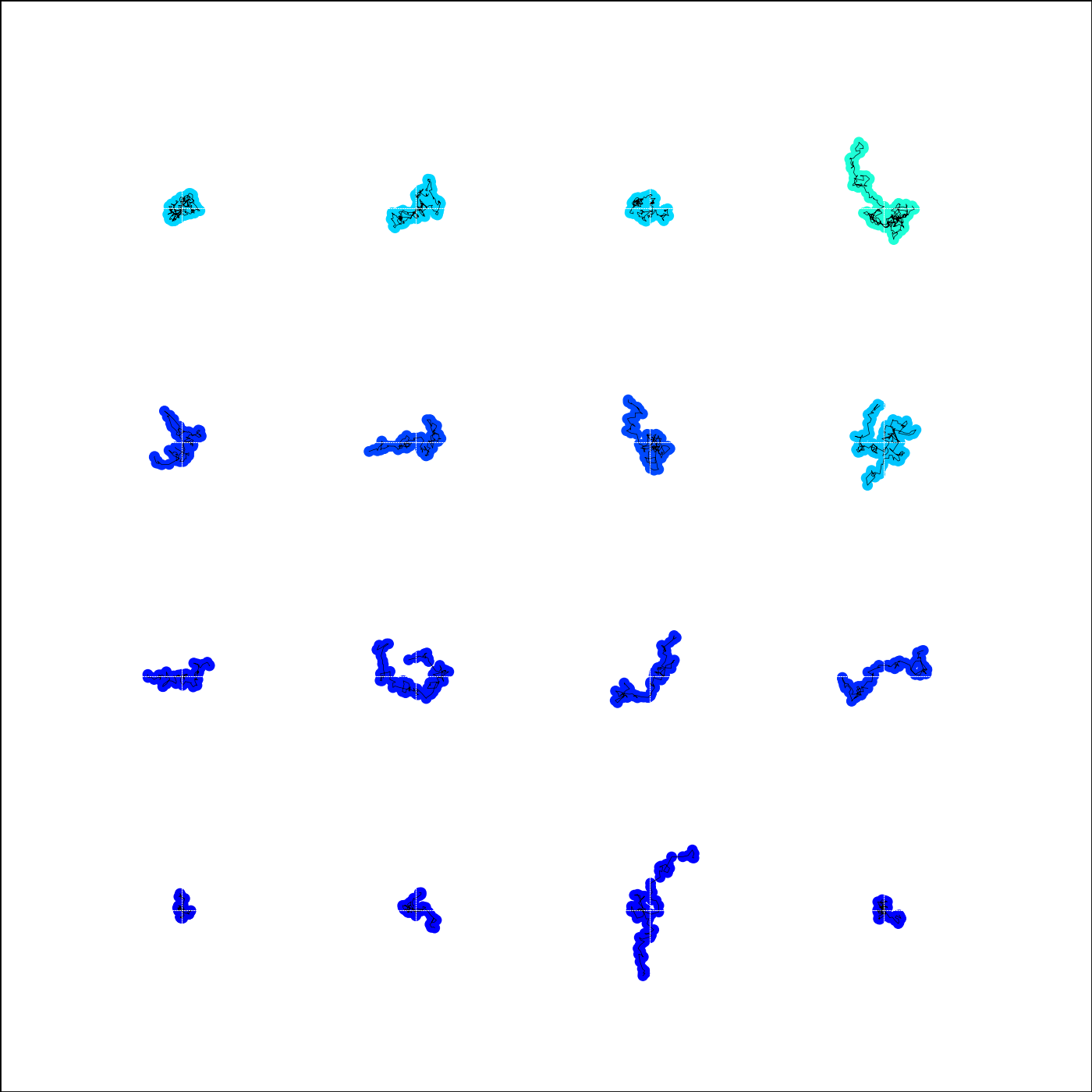

Supplement: Supplementary file 7 — Source Data [file 41467_2021_24223_MOESM7_ESM.zip › z.source-data/si/si_fig08_FS/b/plots/fig08b_upper.png]

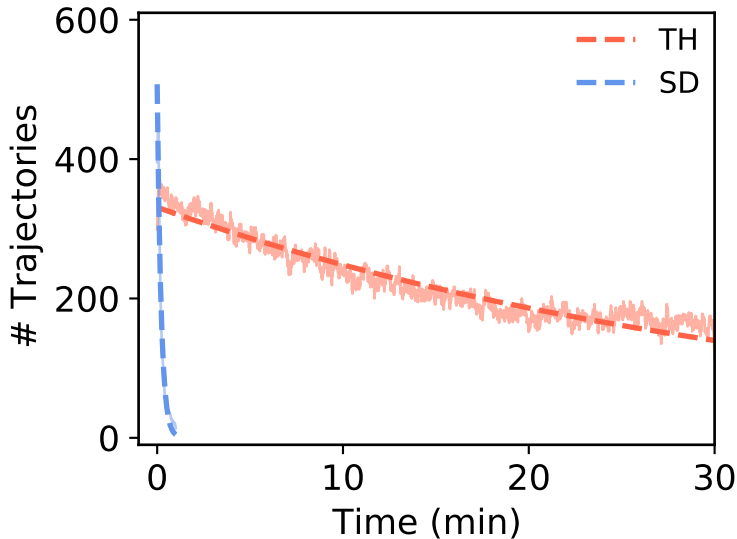

Supplement: Supplementary file 7 — Source Data [file 41467_2021_24223_MOESM7_ESM.zip › z.source-data/si/si_fig09_FS/a-c/plots/fig08a_lower.pdf]

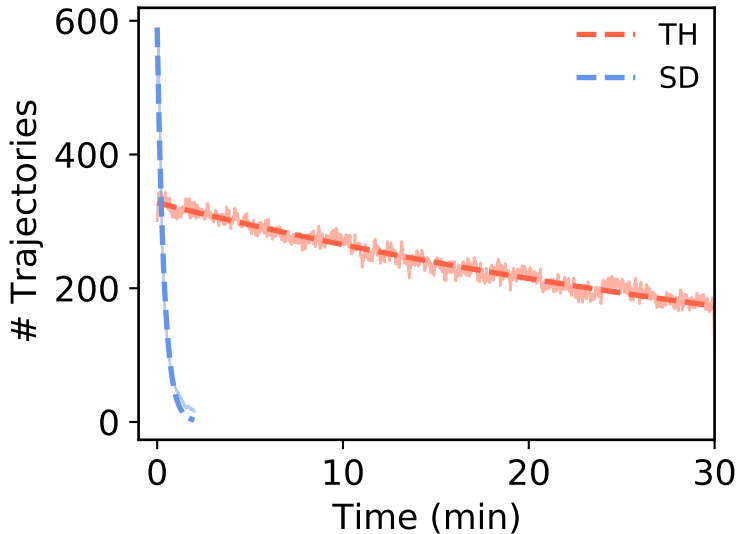

Supplement: Supplementary file 7 — Source Data [file 41467_2021_24223_MOESM7_ESM.zip › z.source-data/si/si_fig09_FS/a-c/plots/fig08a_middle.pdf]

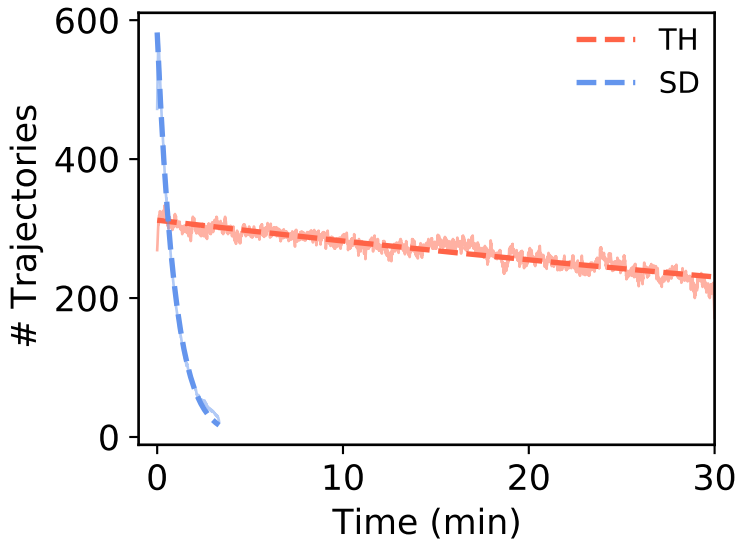

Supplement: Supplementary file 7 — Source Data [file 41467_2021_24223_MOESM7_ESM.zip › z.source-data/si/si_fig09_FS/a-c/plots/fig08a_upper.pdf]

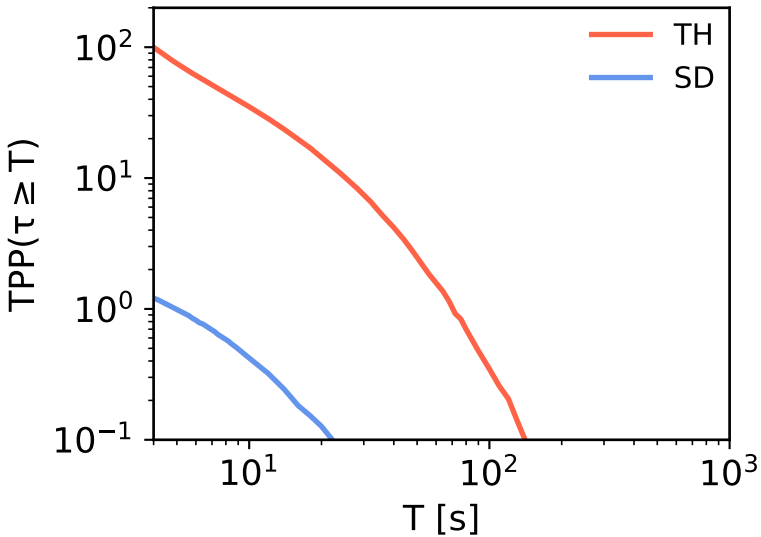

Supplement: Supplementary file 7 — Source Data [file 41467_2021_24223_MOESM7_ESM.zip › z.source-data/si/si_fig09_FS/a-c/plots/fig08b_lower.pdf]

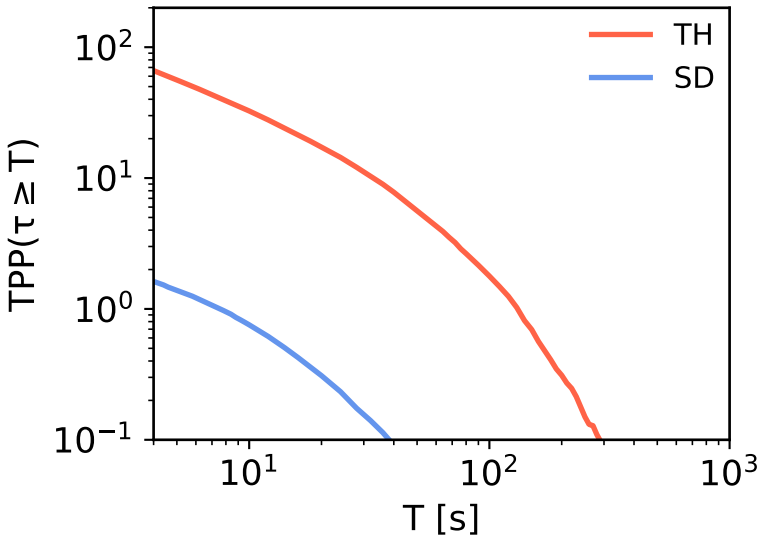

Supplement: Supplementary file 7 — Source Data [file 41467_2021_24223_MOESM7_ESM.zip › z.source-data/si/si_fig09_FS/a-c/plots/fig08b_middle.pdf]

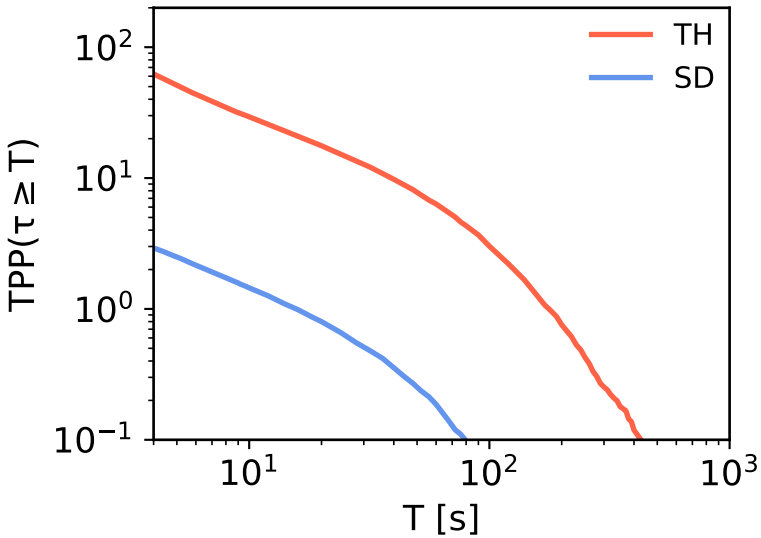

Supplement: Supplementary file 7 — Source Data [file 41467_2021_24223_MOESM7_ESM.zip › z.source-data/si/si_fig09_FS/a-c/plots/fig08b_upper.pdf]

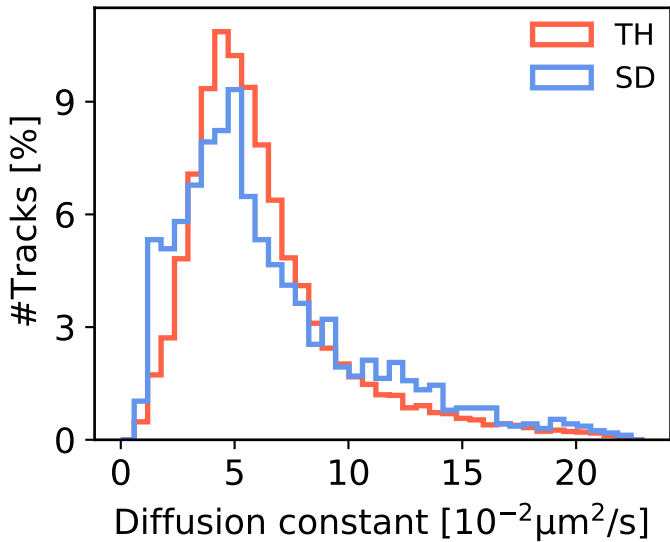

Supplement: Supplementary file 7 — Source Data [file 41467_2021_24223_MOESM7_ESM.zip › z.source-data/si/si_fig09_FS/a-c/plots/fig08c_lower.pdf]

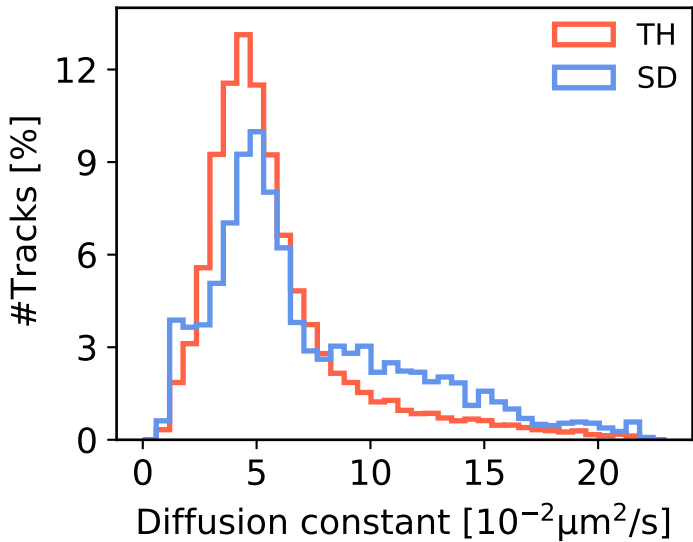

Supplement: Supplementary file 7 — Source Data [file 41467_2021_24223_MOESM7_ESM.zip › z.source-data/si/si_fig09_FS/a-c/plots/fig08c_middle.pdf]

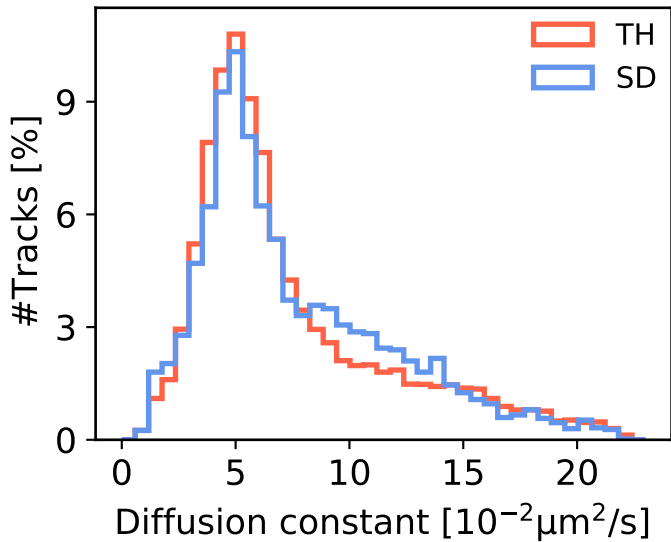

Supplement: Supplementary file 7 — Source Data [file 41467_2021_24223_MOESM7_ESM.zip › z.source-data/si/si_fig09_FS/a-c/plots/fig08c_upper.pdf]

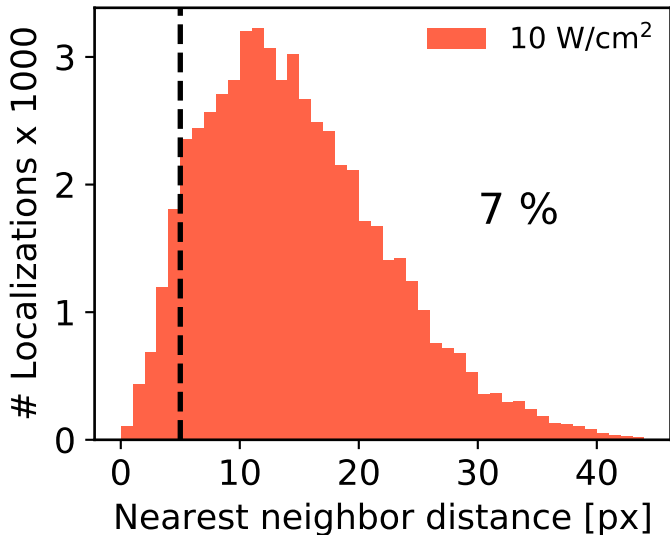

Supplement: Supplementary file 7 — Source Data [file 41467_2021_24223_MOESM7_ESM.zip › z.source-data/si/si_fig10_FS/a/plots/fig10a.pdf]

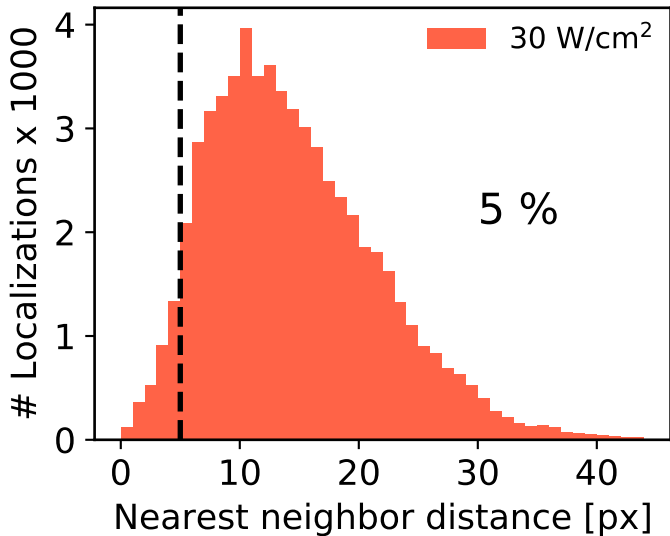

Supplement: Supplementary file 7 — Source Data [file 41467_2021_24223_MOESM7_ESM.zip › z.source-data/si/si_fig10_FS/b/plots/fig10b.pdf]

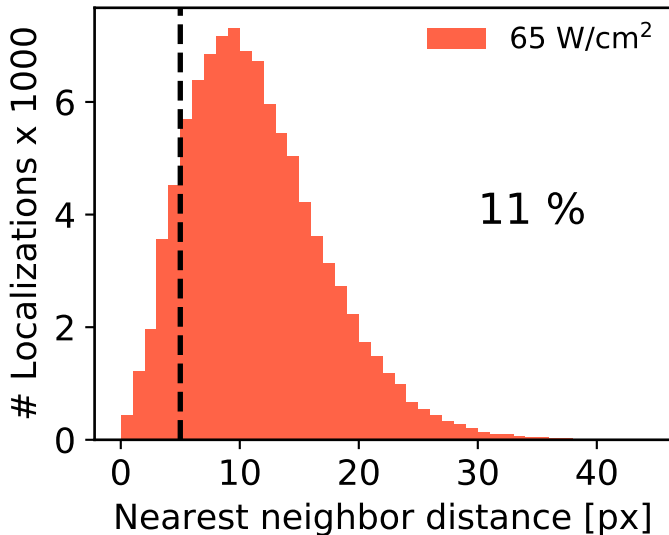

Supplement: Supplementary file 7 — Source Data [file 41467_2021_24223_MOESM7_ESM.zip › z.source-data/si/si_fig10_FS/c/plots/fig10c.pdf]

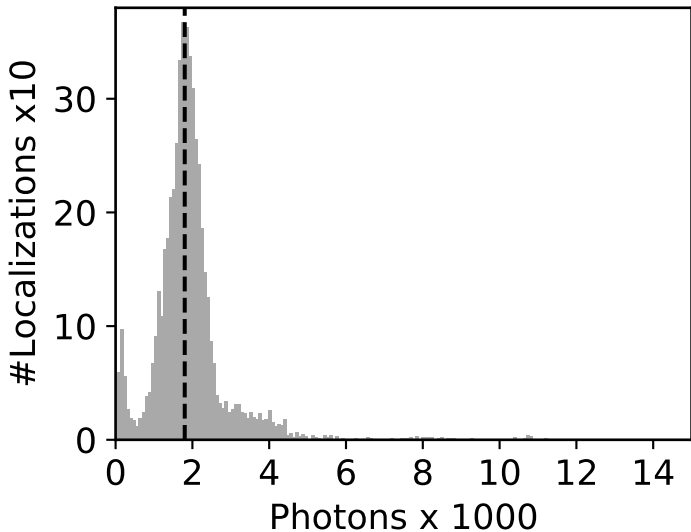

Supplement: Supplementary file 7 — Source Data [file 41467_2021_24223_MOESM7_ESM.zip › z.source-data/si/si_fig11_FS/a-c/plots/fig11a_lower.pdf]

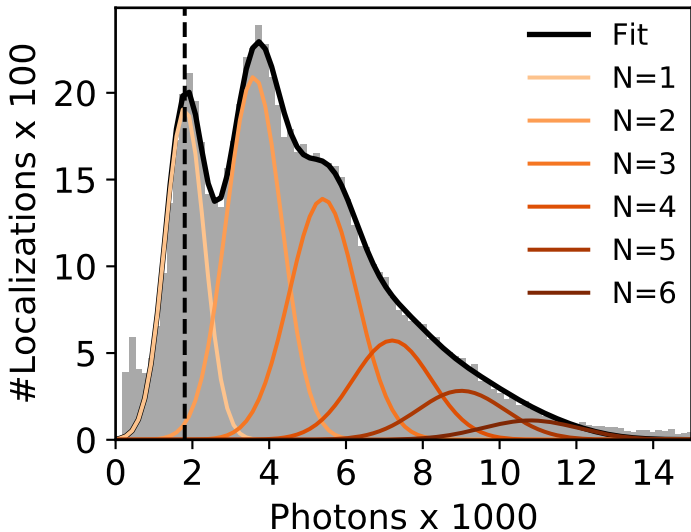

Supplement: Supplementary file 7 — Source Data [file 41467_2021_24223_MOESM7_ESM.zip › z.source-data/si/si_fig11_FS/a-c/plots/fig11a_upper.pdf]

#Photons x1000

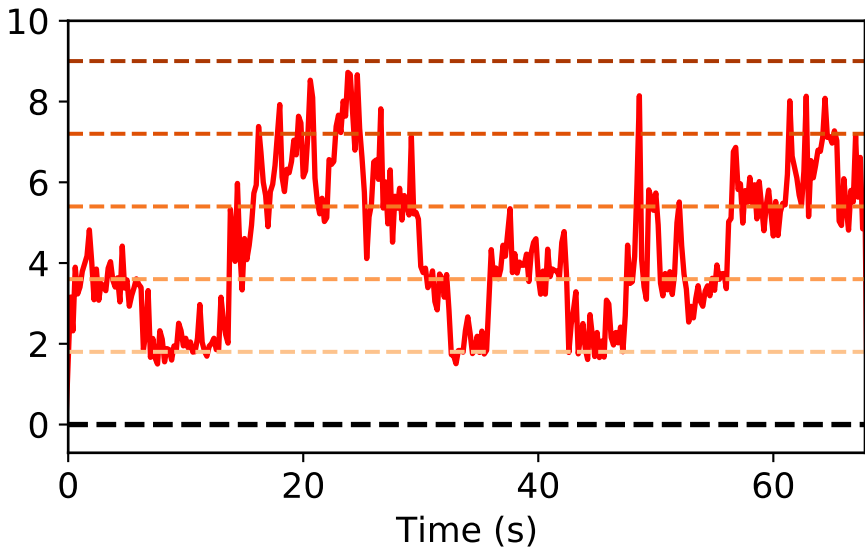

Supplement: Supplementary file 7 — Source Data [file 41467_2021_24223_MOESM7_ESM.zip › z.source-data/si/si_fig11_FS/a-c/plots/fig11b.pdf]

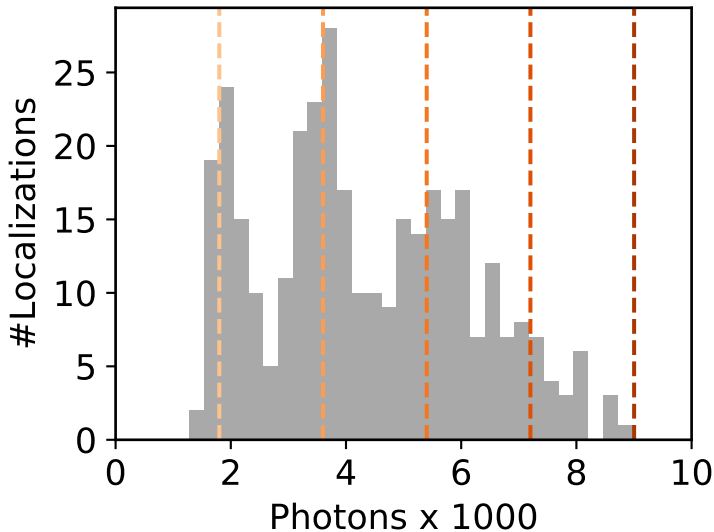

Supplement: Supplementary file 7 — Source Data [file 41467_2021_24223_MOESM7_ESM.zip › z.source-data/si/si_fig11_FS/a-c/plots/fig11c.pdf]

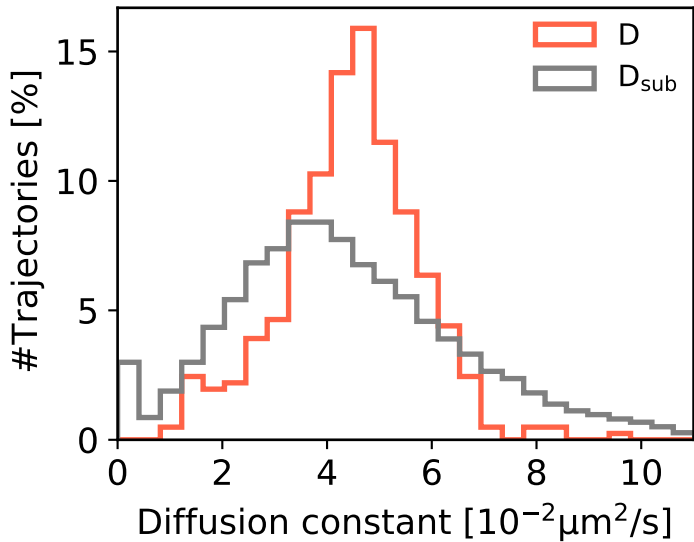

Supplement: Supplementary file 7 — Source Data [file 41467_2021_24223_MOESM7_ESM.zip › z.source-data/si/si_fig12_FS/a-b/plots/fig12a.pdf]

#Trajectories [%]

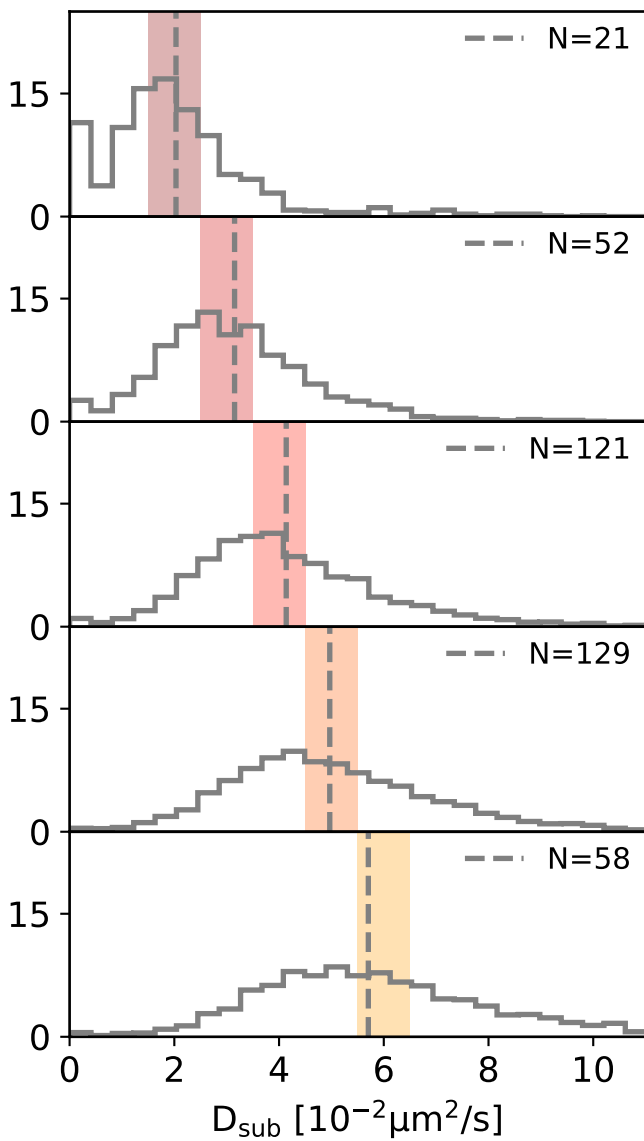

Supplement: Supplementary file 7 — Source Data [file 41467_2021_24223_MOESM7_ESM.zip › z.source-data/si/si_fig12_FS/a-b/plots/fig12b.pdf]

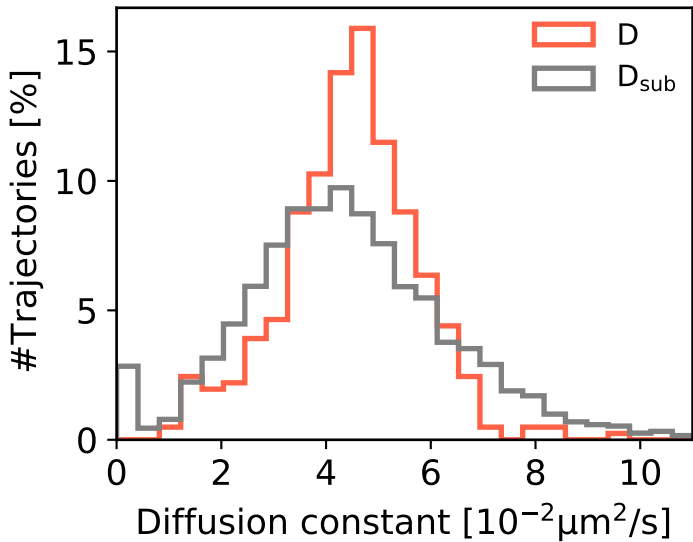

Supplement: Supplementary file 7 — Source Data [file 41467_2021_24223_MOESM7_ESM.zip › z.source-data/si/si_fig12_FS/c-d/plots/fig12c.pdf]

#Trajectories [%]

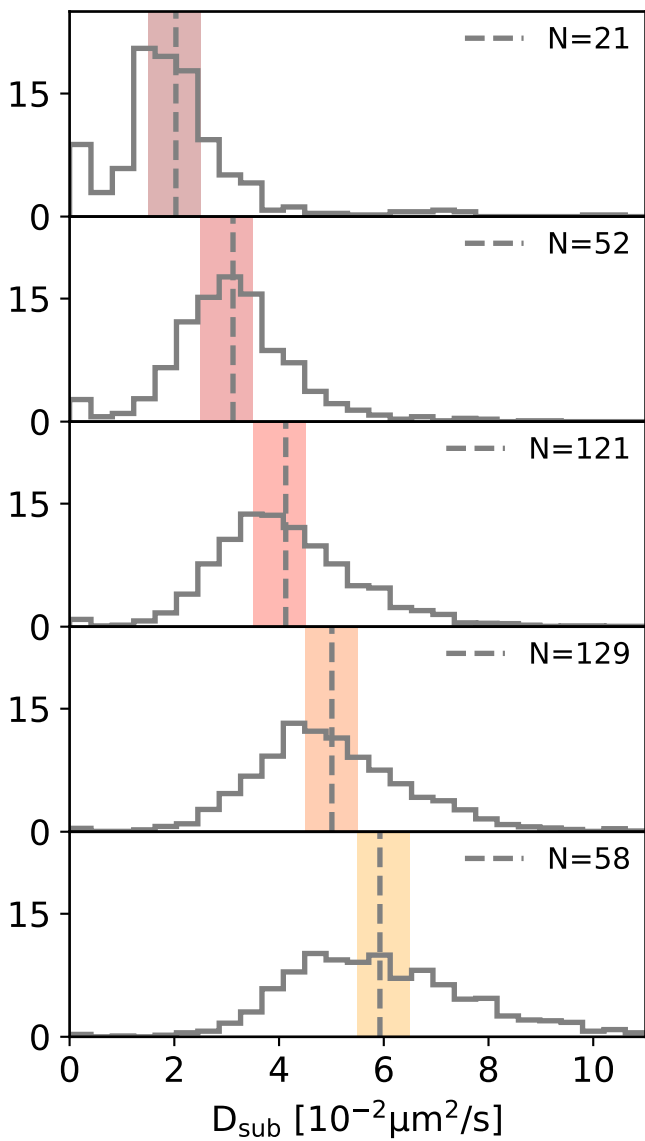

Supplement: Supplementary file 7 — Source Data [file 41467_2021_24223_MOESM7_ESM.zip › z.source-data/si/si_fig12_FS/c-d/plots/fig12d.pdf]

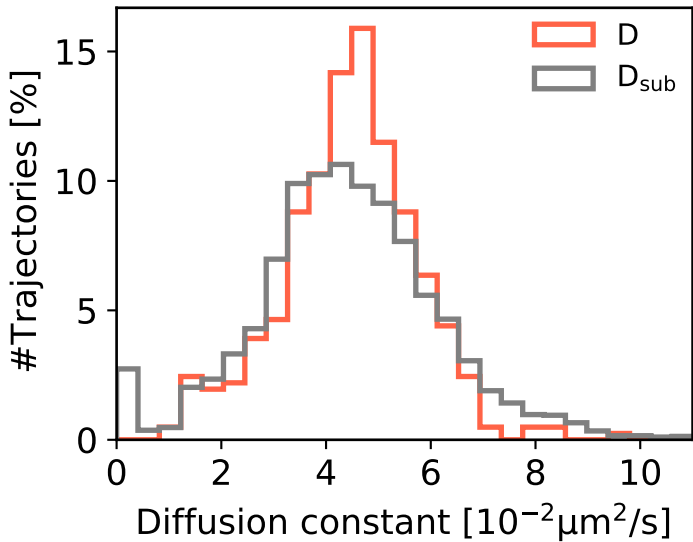

Supplement: Supplementary file 7 — Source Data [file 41467_2021_24223_MOESM7_ESM.zip › z.source-data/si/si_fig12_FS/e-f/plots/fig12e.pdf]

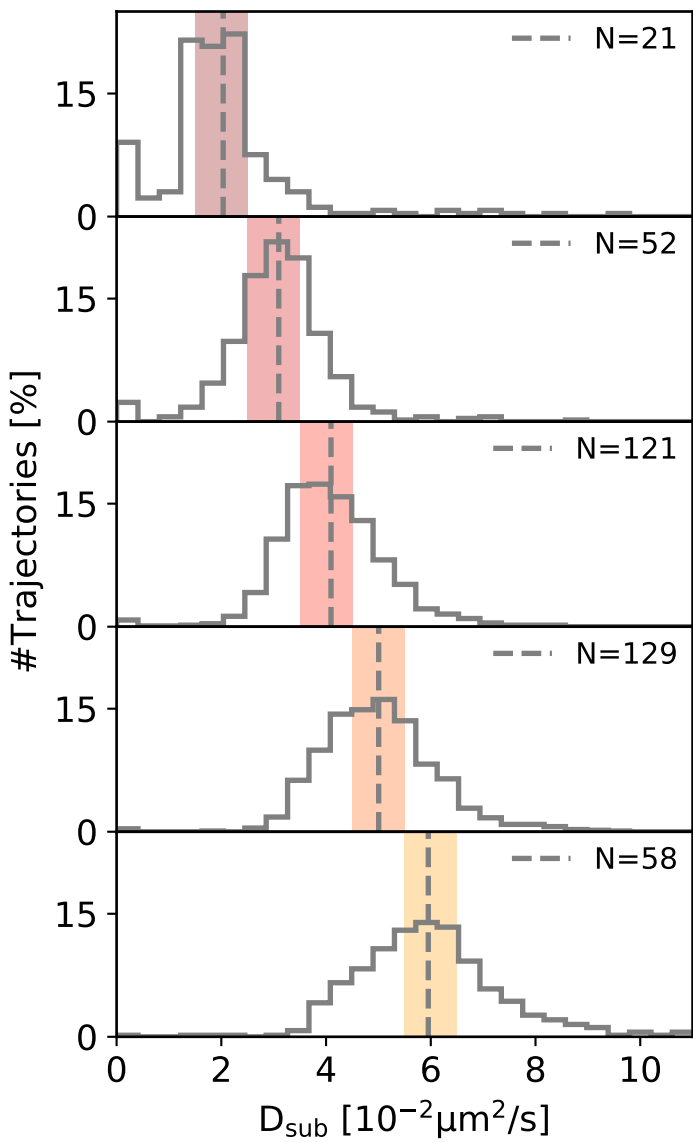

Supplement: Supplementary file 7 — Source Data [file 41467_2021_24223_MOESM7_ESM.zip › z.source-data/si/si_fig12_FS/e-f/plots/fig12f.pdf]

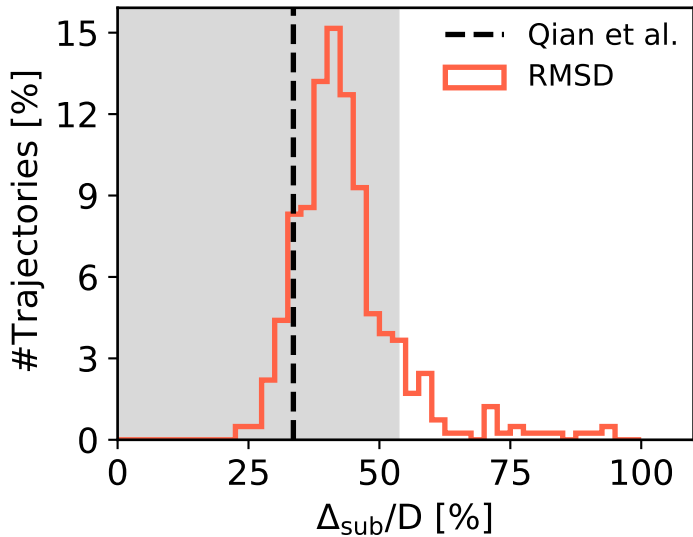

Supplement: Supplementary file 7 — Source Data [file 41467_2021_24223_MOESM7_ESM.zip › z.source-data/si/si_fig13_FS/a/plots/fig13a.pdf]

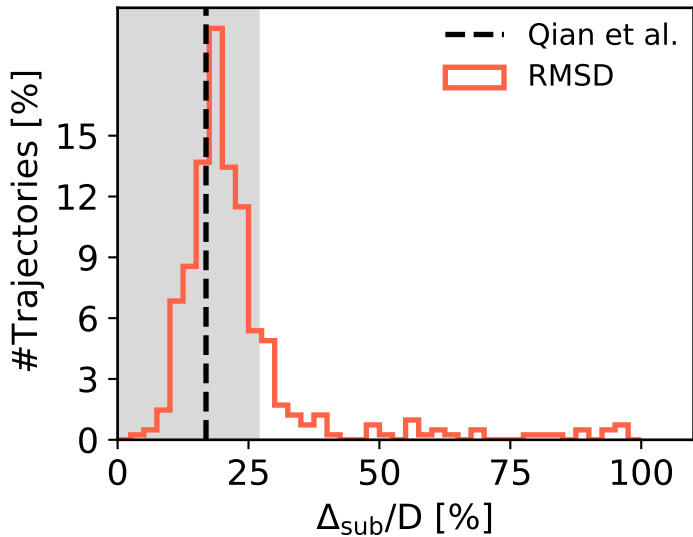

Supplement: Supplementary file 7 — Source Data [file 41467_2021_24223_MOESM7_ESM.zip › z.source-data/si/si_fig13_FS/b/plots/fig13b.pdf]

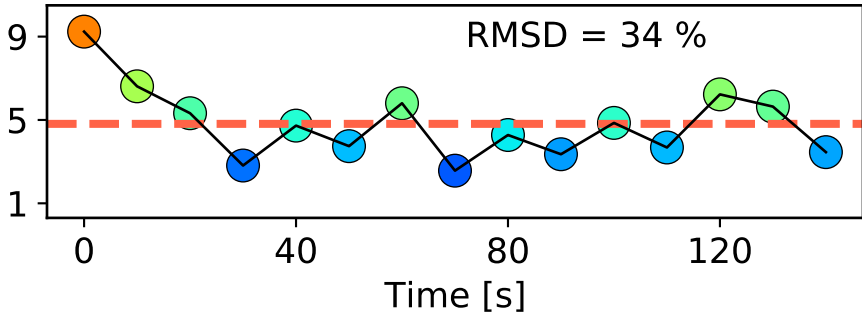

Supplement: Supplementary file 7 — Source Data [file 41467_2021_24223_MOESM7_ESM.zip › z.source-data/si/si_fig14_FS/a/plots/fig14a_lower.pdf]

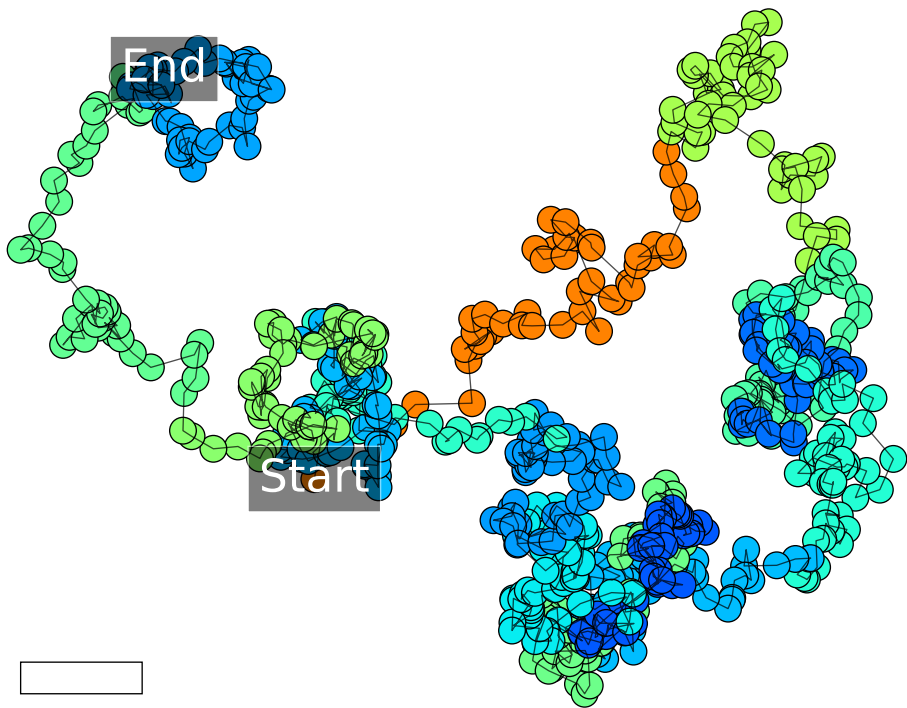

Supplement: Supplementary file 7 — Source Data [file 41467_2021_24223_MOESM7_ESM.zip › z.source-data/si/si_fig14_FS/a/plots/fig14a_upper.pdf]

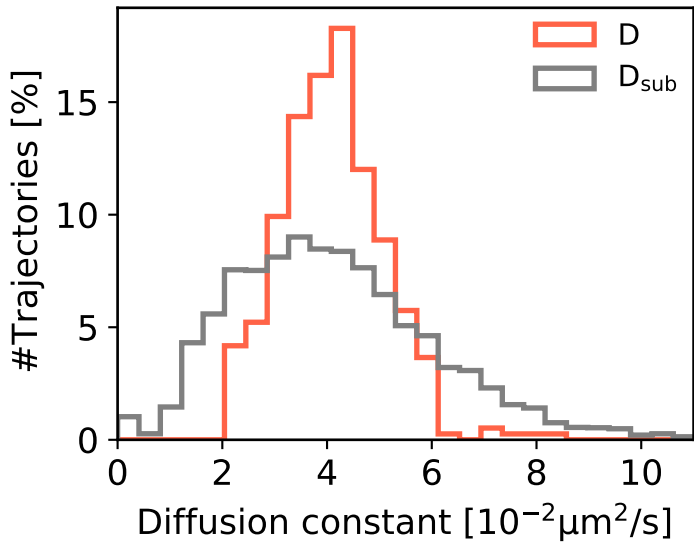

Supplement: Supplementary file 7 — Source Data [file 41467_2021_24223_MOESM7_ESM.zip › z.source-data/si/si_fig14_FS/b/plots/fig14b.pdf]

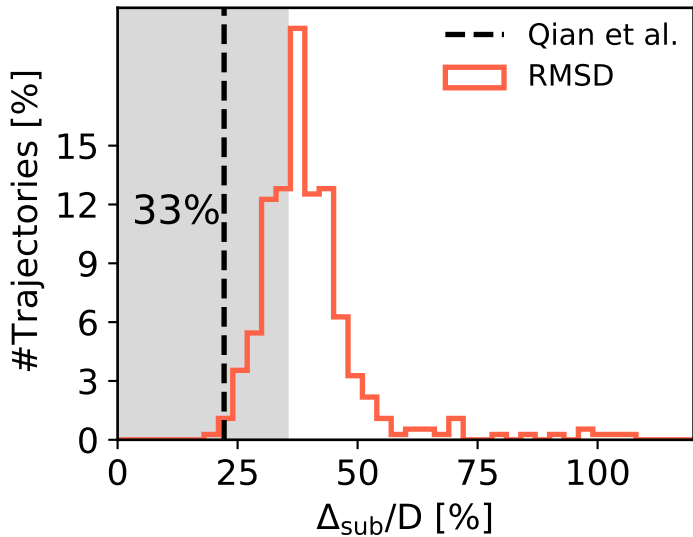

Supplement: Supplementary file 7 — Source Data [file 41467_2021_24223_MOESM7_ESM.zip › z.source-data/si/si_fig14_FS/c/plots/fig14c.pdf]

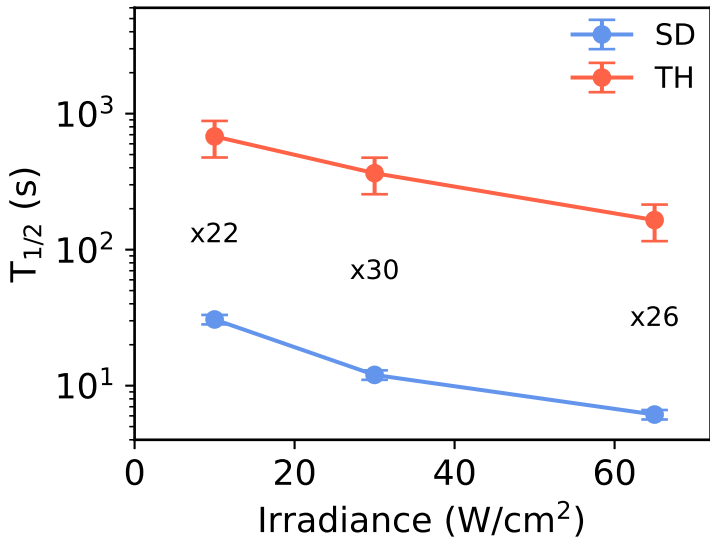

Supplement: Supplementary file 7 — Source Data [file 41467_2021_24223_MOESM7_ESM.zip › z.source-data/si/si_fig15_JS/b-left/plots/si_fig15b-Thalf.pdf]

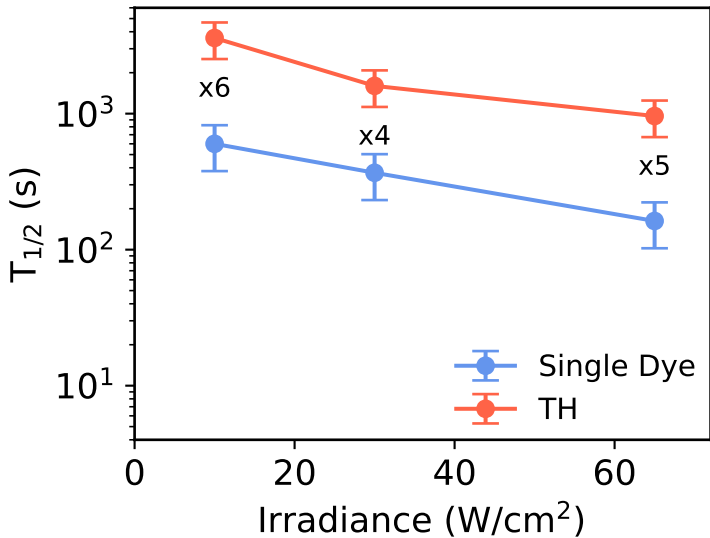

Supplement: Supplementary file 7 — Source Data [file 41467_2021_24223_MOESM7_ESM.zip › z.source-data/si/si_fig15_JS/b-right/plots/si_fig15b-Thalf-POC.pdf]

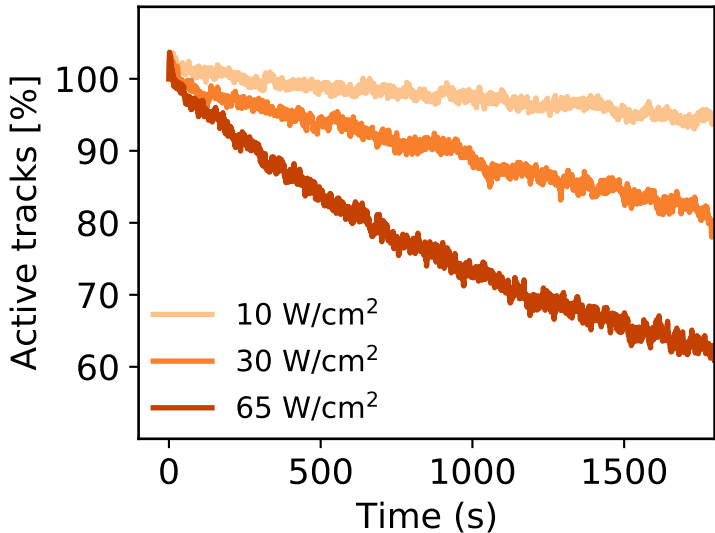

Supplement: Supplementary file 7 — Source Data [file 41467_2021_24223_MOESM7_ESM.zip › z.source-data/si/si_fig15_JS/c-left/plots/si_fig15c-left_tracks.pdf]

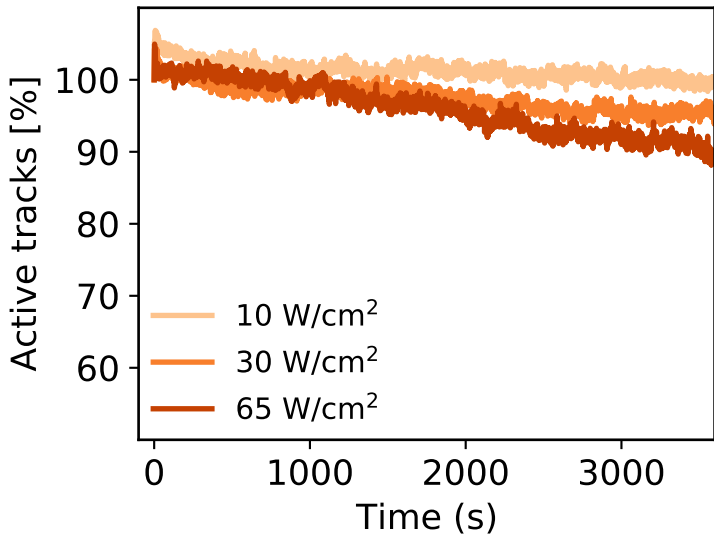

Supplement: Supplementary file 7 — Source Data [file 41467_2021_24223_MOESM7_ESM.zip › z.source-data/si/si_fig15_JS/c-right/plots/si_fig15c-righttracks.pdf]

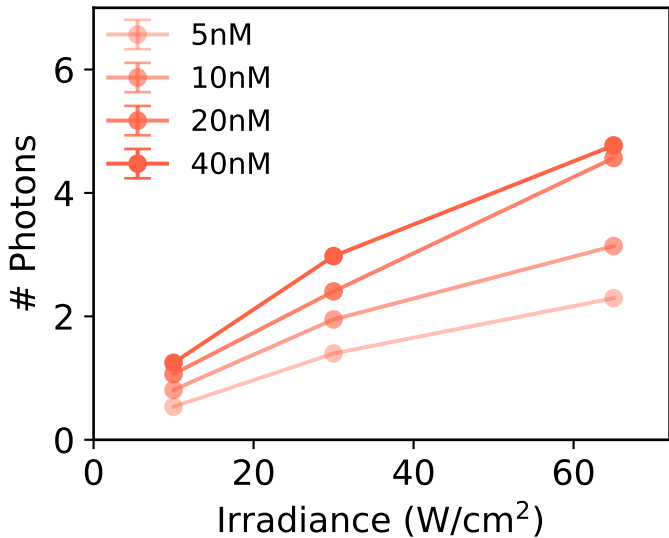

Supplement: Supplementary file 7 — Source Data [file 41467_2021_24223_MOESM7_ESM.zip › z.source-data/si/si_fig15_JS/d-left/plots/si_fig15d-left_photons.pdf]

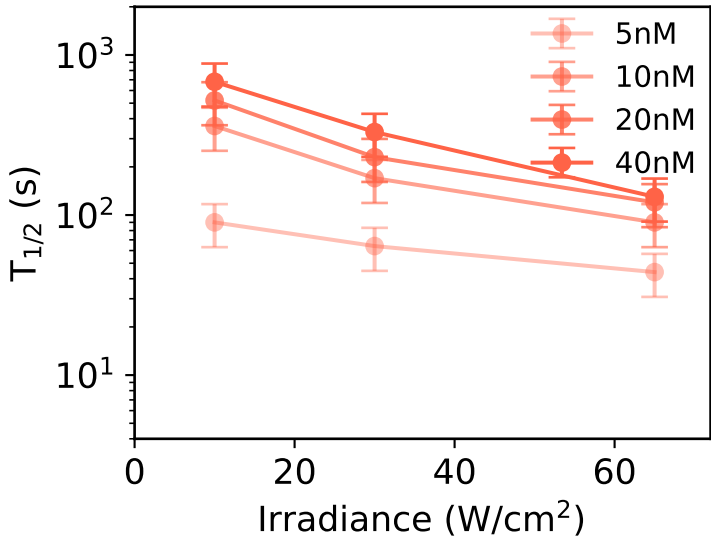

Supplement: Supplementary file 7 — Source Data [file 41467_2021_24223_MOESM7_ESM.zip › z.source-data/si/si_fig15_JS/d-left/plots/si_fig15d-left_Thalf.pdf]

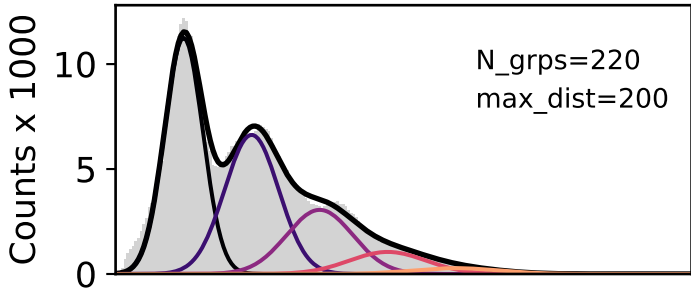

Supplement: Supplementary file 7 — Source Data [file 41467_2021_24223_MOESM7_ESM.zip › z.source-data/si/si_fig15_JS/d-right/plots/si_fig15d-right_10nM.pdf]

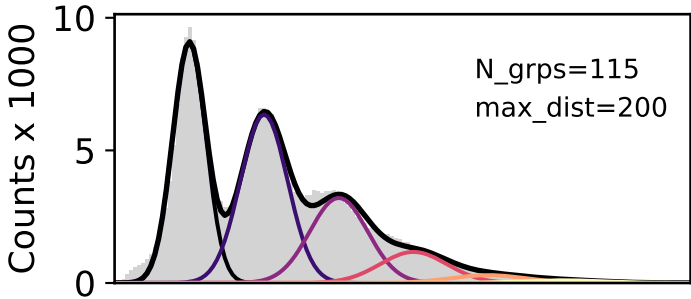

Supplement: Supplementary file 7 — Source Data [file 41467_2021_24223_MOESM7_ESM.zip › z.source-data/si/si_fig15_JS/d-right/plots/si_fig15d-right_20nM.pdf]

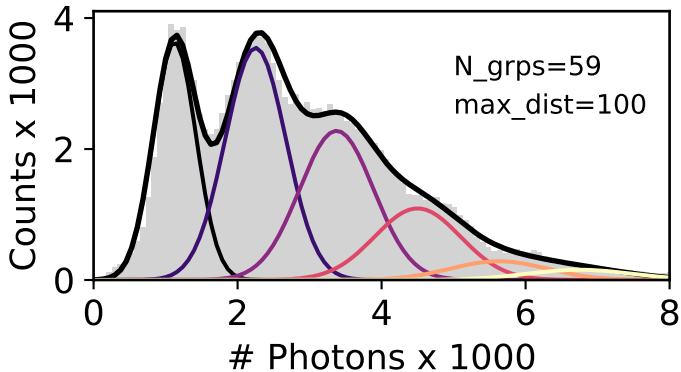

Supplement: Supplementary file 7 — Source Data [file 41467_2021_24223_MOESM7_ESM.zip › z.source-data/si/si_fig15_JS/d-right/plots/si_fig15d-right_40nM.pdf]

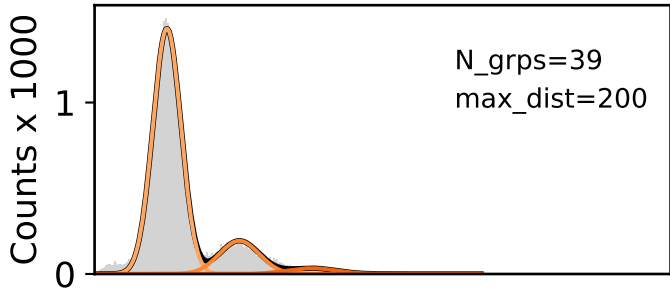

Supplement: Supplementary file 7 — Source Data [file 41467_2021_24223_MOESM7_ESM.zip › z.source-data/si/si_fig15_JS/d-right/plots/si_fig15d-right_5nM.pdf]

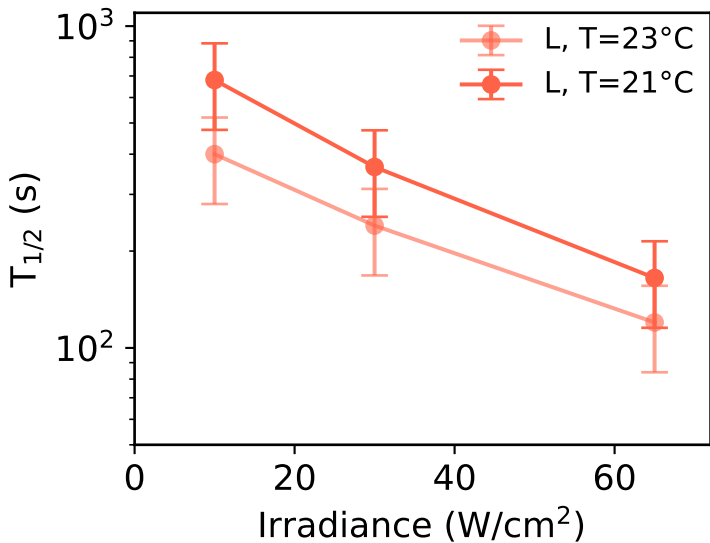

Supplement: Supplementary file 7 — Source Data [file 41467_2021_24223_MOESM7_ESM.zip › z.source-data/si/si_fig15_JS/e/plots/si_fig15e_Thalf.pdf]

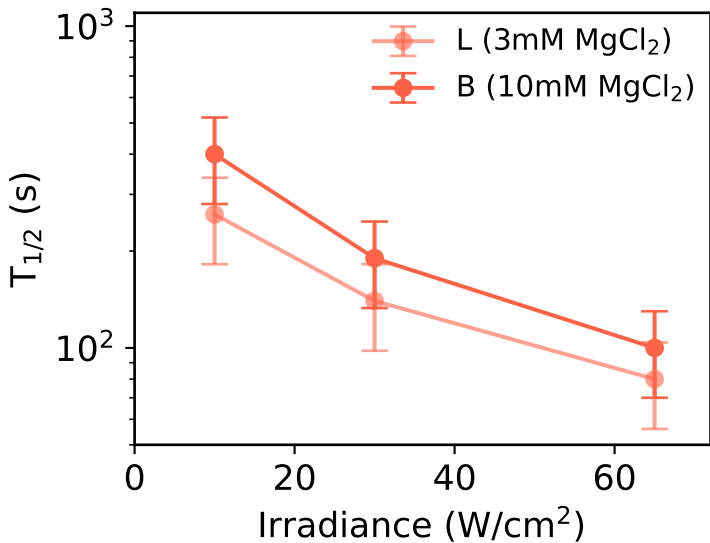

Supplement: Supplementary file 7 — Source Data [file 41467_2021_24223_MOESM7_ESM.zip › z.source-data/si/si_fig15_JS/e/plots/si_fig15f_Thalf.pdf]

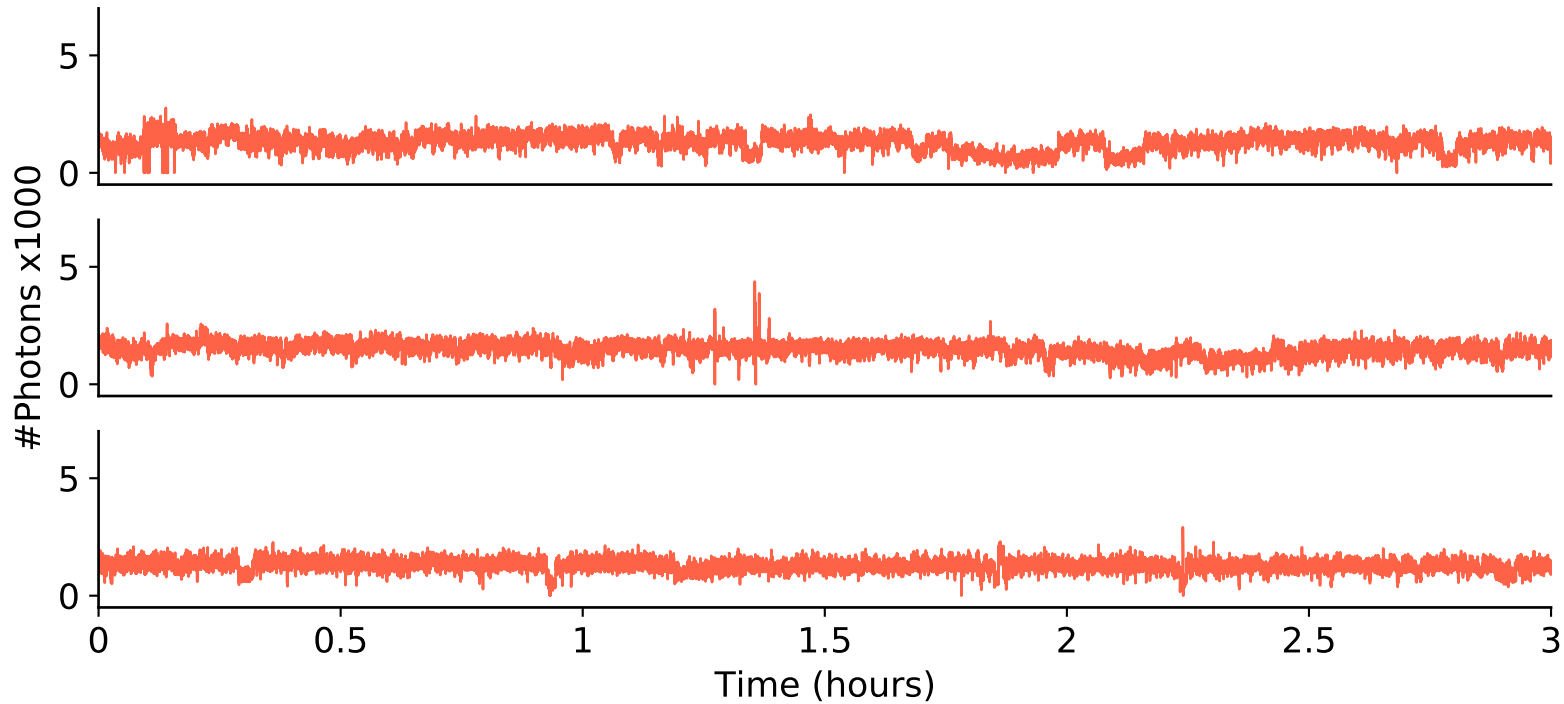

Supplement: Supplementary file 7 — Source Data [file 41467_2021_24223_MOESM7_ESM.zip › z.source-data/si/si_fig16_JS/b/plots/fig16b_th_trace.pdf]

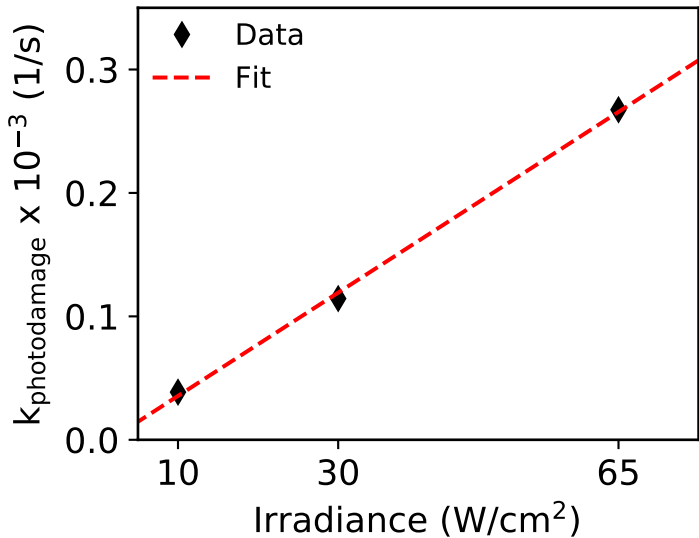

Supplement: Supplementary file 7 — Source Data [file 41467_2021_24223_MOESM7_ESM.zip › z.source-data/si/si_fig17_JS/plots/b_fit.pdf]

#Trajectories

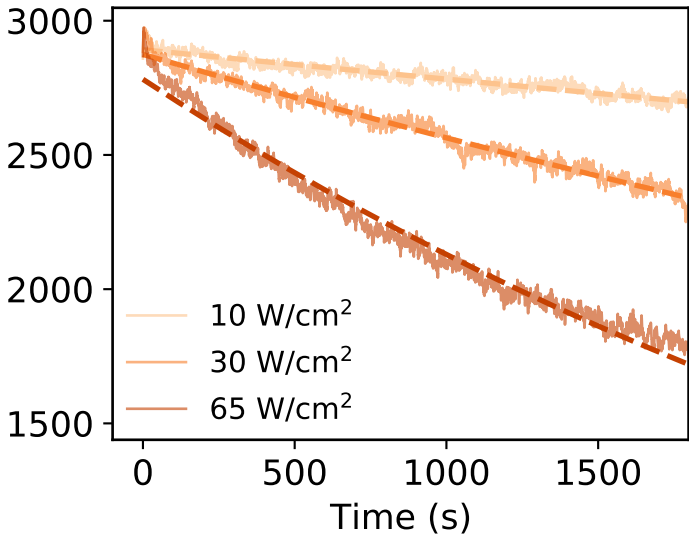

Supplement: Supplementary file 7 — Source Data [file 41467_2021_24223_MOESM7_ESM.zip › z.source-data/si/si_fig17_JS/plots/si_fig17a_tracks.pdf]

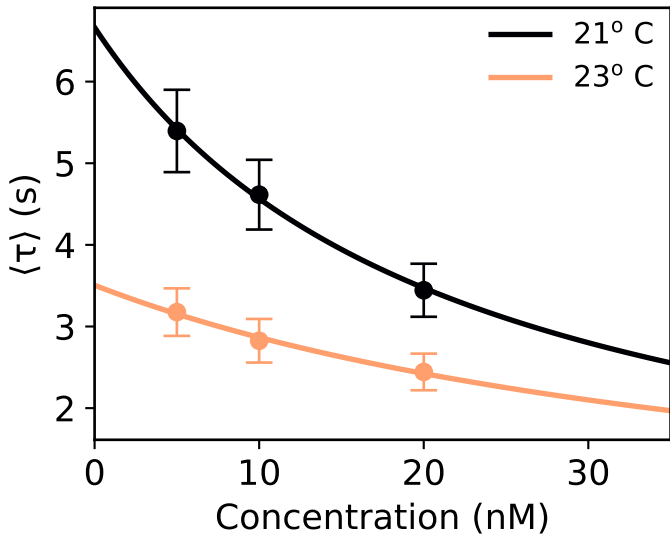

Supplement: Supplementary file 7 — Source Data [file 41467_2021_24223_MOESM7_ESM.zip › z.source-data/si/si_fig18_FS/a-c/plots/fig18a.pdf]

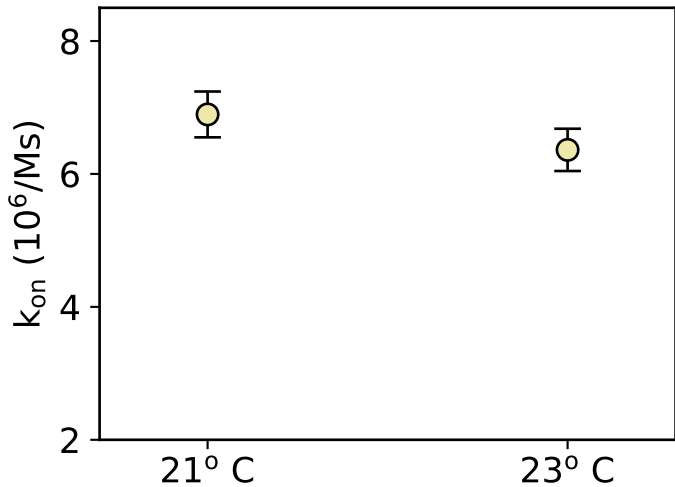

Supplement: Supplementary file 7 — Source Data [file 41467_2021_24223_MOESM7_ESM.zip › z.source-data/si/si_fig18_FS/a-c/plots/fig18b.pdf]

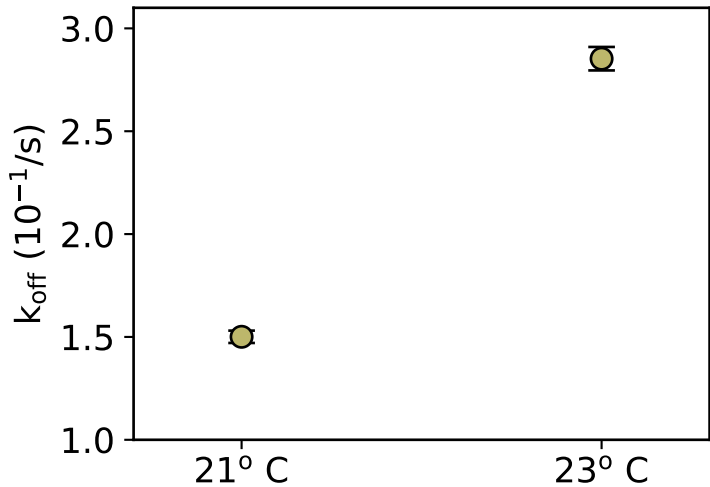

Supplement: Supplementary file 7 — Source Data [file 41467_2021_24223_MOESM7_ESM.zip › z.source-data/si/si_fig18_FS/a-c/plots/fig18c.pdf]

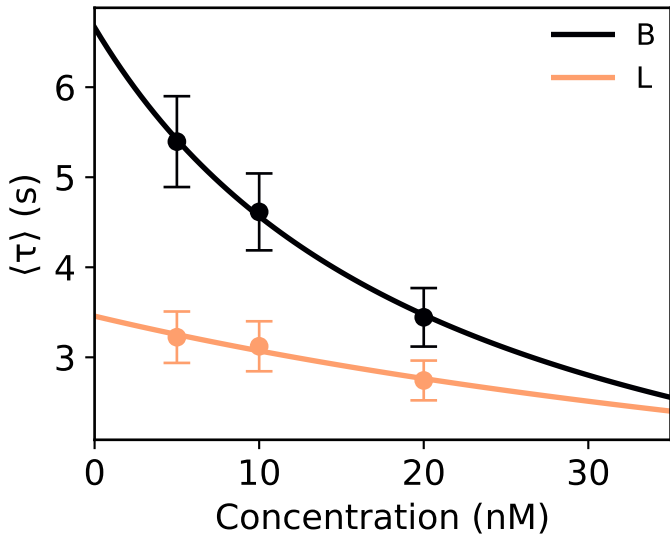

Supplement: Supplementary file 7 — Source Data [file 41467_2021_24223_MOESM7_ESM.zip › z.source-data/si/si_fig19_FS/a-c/plots/fig19a.pdf]

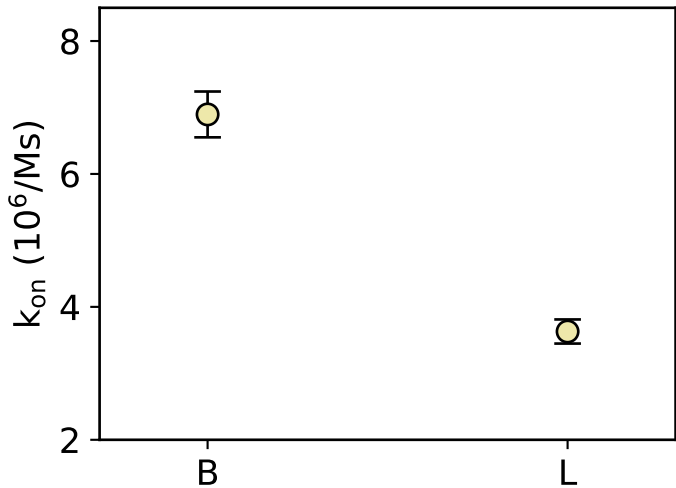

Supplement: Supplementary file 7 — Source Data [file 41467_2021_24223_MOESM7_ESM.zip › z.source-data/si/si_fig19_FS/a-c/plots/fig19b.pdf]

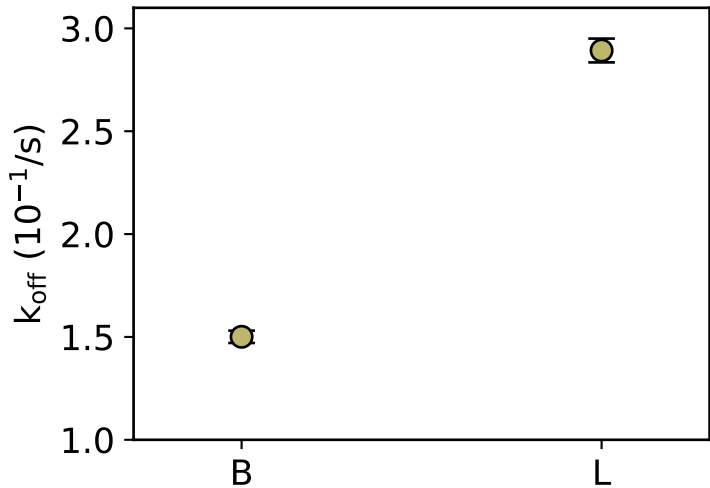

Supplement: Supplementary file 7 — Source Data [file 41467_2021_24223_MOESM7_ESM.zip › z.source-data/si/si_fig19_FS/a-c/plots/fig19c.pdf]

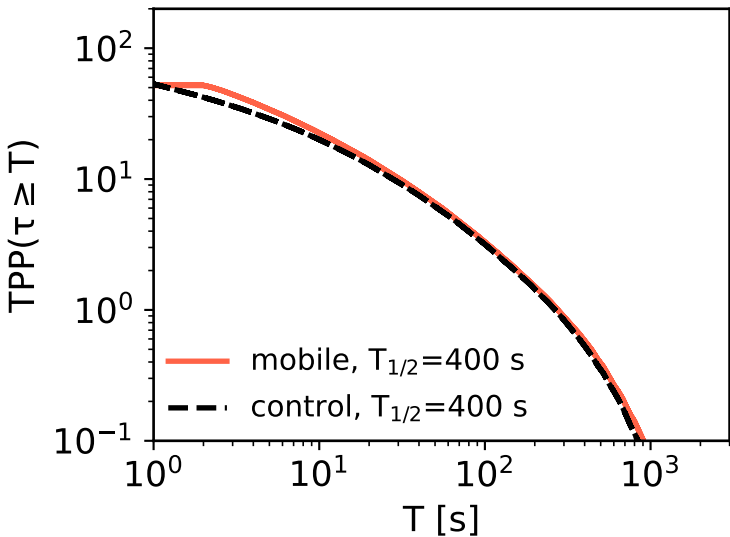

Supplement: Supplementary file 7 — Source Data [file 41467_2021_24223_MOESM7_ESM.zip › z.source-data/si/si_fig20_FS/plots/fig20.pdf]

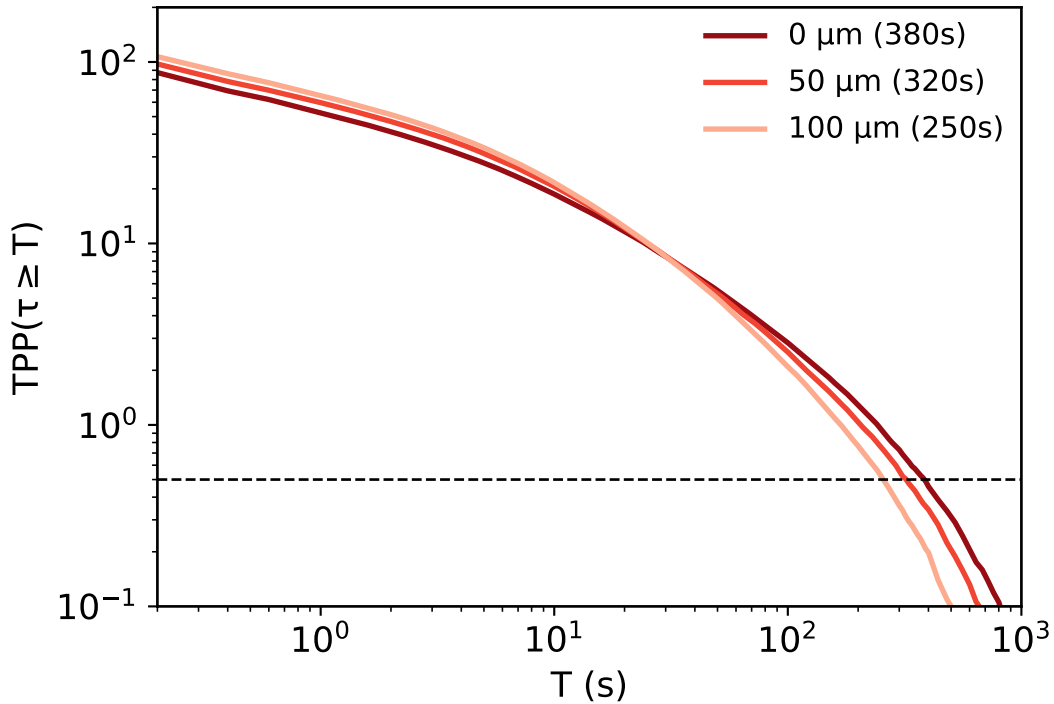

Supplement: Supplementary file 7 — Source Data [file 41467_2021_24223_MOESM7_ESM.zip › z.source-data/si/si_fig21_JS/b/plots/si_fig21b_TPP.pdf]

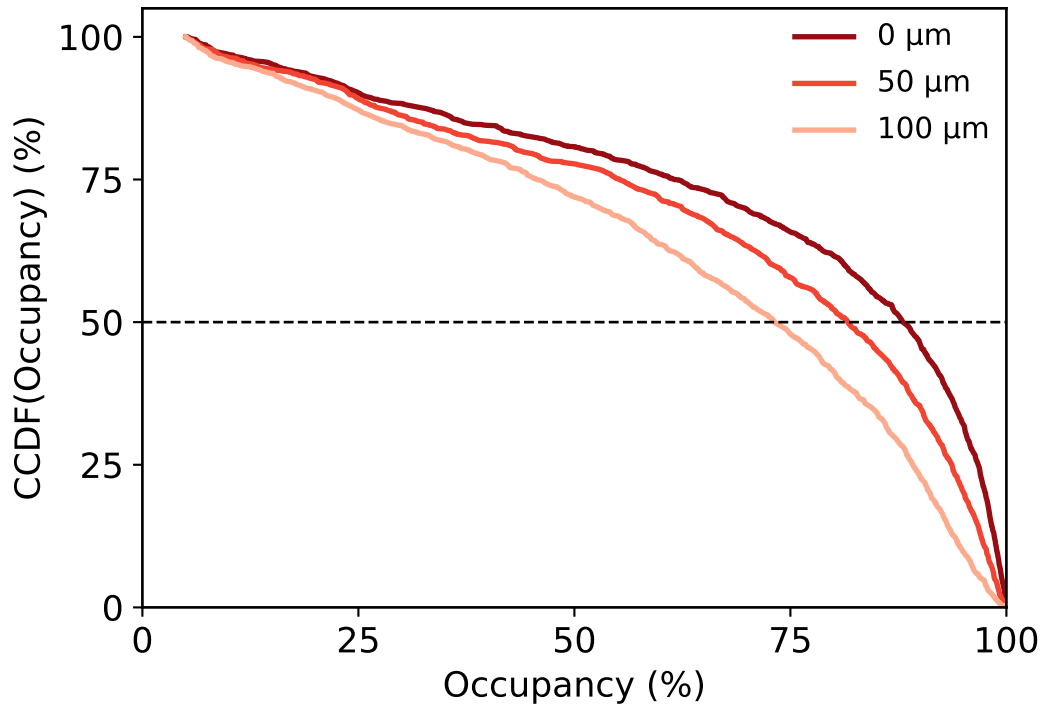

Supplement: Supplementary file 7 — Source Data [file 41467_2021_24223_MOESM7_ESM.zip › z.source-data/si/si_fig21_JS/c/plots/si_fig21b_occ.pdf]

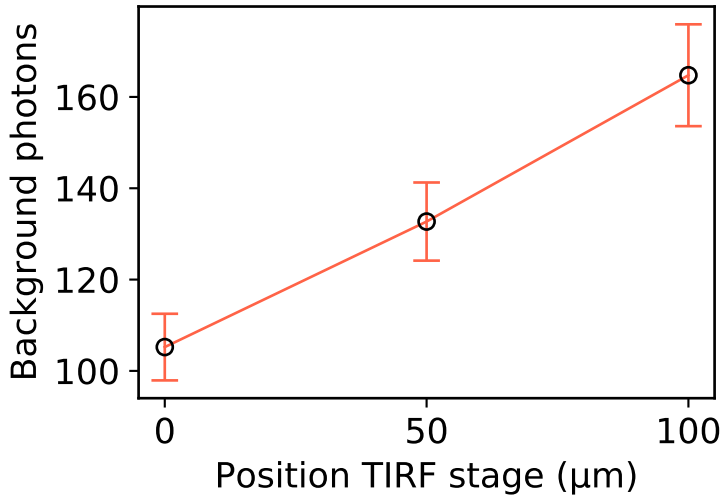

Supplement: Supplementary file 7 — Source Data [file 41467_2021_24223_MOESM7_ESM.zip › z.source-data/si/si_fig21_JS/d/plots/si_fig21d_bg.pdf]
